# Controlling the structure and photophysics of fluorophore dimers using multiple cucurbit[8]uril clampings†

**DOI:** 10.1039/c9sc04587b

**Published:** 2019-12-06

**Authors:** Guanglu Wu, Youn Jue Bae, Magdalena Olesińska, Daniel Antón-García, István Szabó, Edina Rosta, Michael R. Wasielewski, Oren A. Scherman

**Affiliations:** Melville Laboratory for Polymer Synthesis, Department of Chemistry, University of Cambridge Lensfield Road Cambridge CB2 1EW UK oas23@cam.ac.uk; Department of Chemistry, Institute for Sustainability and Energy at Northwestern, Northwestern University Evanston Illinois 60208-3113 USA; Department of Chemistry, University of Cambridge Lensfield Road Cambridge CB2 1EW UK; Department of Chemistry, King's College London 7 Trinity Street London SE1 1DB UK

## Abstract

A modular strategy has been employed to develop a new class of fluorescent molecules, which generates discrete, dimeric stacked fluorophores upon complexation with multiple cucurbit[8]uril macrocycles. The multiple constraints result in a “static” complex (remaining as a single entity for more than 30 ms) and facilitate fluorophore coupling in the ground state, showing a significant bathochromic shift in absorption and emission. This modular design is surprisingly applicable and flexible and has been validated through an investigation of nine different fluorophore cores ranging in size, shape, and geometric variation of their clamping modules. All fluorescent dimers evaluated can be photo-excited to atypical excimer-like states with elongated excited lifetimes (up to 37 ns) and substantially high quantum yields (up to 1). This strategy offers a straightforward preparation of discrete fluorophore dimers, providing promising model systems with explicitly stable dimeric structures and tunable photophysical features, which can be utilized to study various intermolecular processes.

## Abstract 2

Dimerisation of a wide range of fluorophores through multiple CB[8] clampings leads to constrained intracomplex motion and distinct photophysical properties.

## Graphical abstract

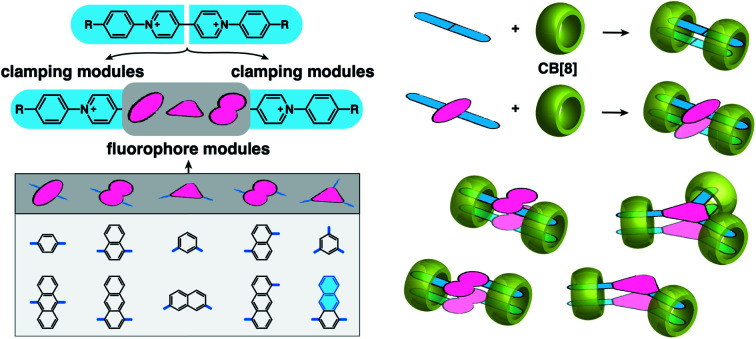

## 1 Introduction

Coupling two fluorophores within a sufficiently short distance for an extended period of time is crucial for both theoretical and experimental investigation of intermolecular processes such as charge transfer,^1^ excimer formation,^2,3^ long- or short-range exciton coupling,^4,5^ and singlet fission.^6–8^ Stacking together precisely two fluorophores in an aqueous solution, however, remains a substantial challenge as most aromatic hydrocarbons show a tendency to aggregate unpredictably (forming clusters of arbitrary numbers of molecules).^9–11^ To prevent fluorophores from aggregation in aqueous solution, a supramolecular approach has been established to “mechanically” separate fluorescent molecules through encapsulation by macrocycles.^12–16^ A popular class of macrocyclic hosts utilized for this purpose is cucurbit[*n*]uril (CB[*n*], *n* = 5–8, 10), which contains a cavity that enables the inclusion of various guest molecules and exhibits particularly high affinity towards positively-charged species.^17,18^ As an example, CB[7] is a promising host for the complexation of various fluorescent dyes,^19^ resulting in significant changes in photophysical properties such as anti-photobleaching^20^ and emission enhancement.^21–23^ This is attributed to the hydrophobic environment provided by the CB cavity as well as mechanical protection by the macrocycle against aggregation and quenching.^12^

Dimeric fluorophore stacking, however, is unlikely to be realized by CB[7]-mediated complexation as its relatively small cavity only allows the complexation with one single guest molecule or, more strictly speaking, one binding moiety on a guest molecule. On the other hand, CB[8], a larger cucurbituril homologue, is capable of simultaneously encapsulating two guest moieties yielding either a heteroternary^24^ or homoternary complex.^25^ Although CB[8]-mediated ternary complexation may achieve stacking of two fluorophores, several limitations exist as the fluorophores are required to have the right shape, size and charge distribution to undergo complexation with CB[8].^26^ Moreover, they must align along the principal symmetry axis of the CB cavity limiting the way in which they stack.^27^ In case of a stepwise complexation of two guests with CB[8], formation of a dynamic ternary complex is evident by the significant signal broadening in NMR spectra.^24,25^ This dynamic complex results in a short-lived coupling between the two stacked fluorophores that is insufficient to allow for the investigation of specific intermolecular processes.

**Fig. 1:**
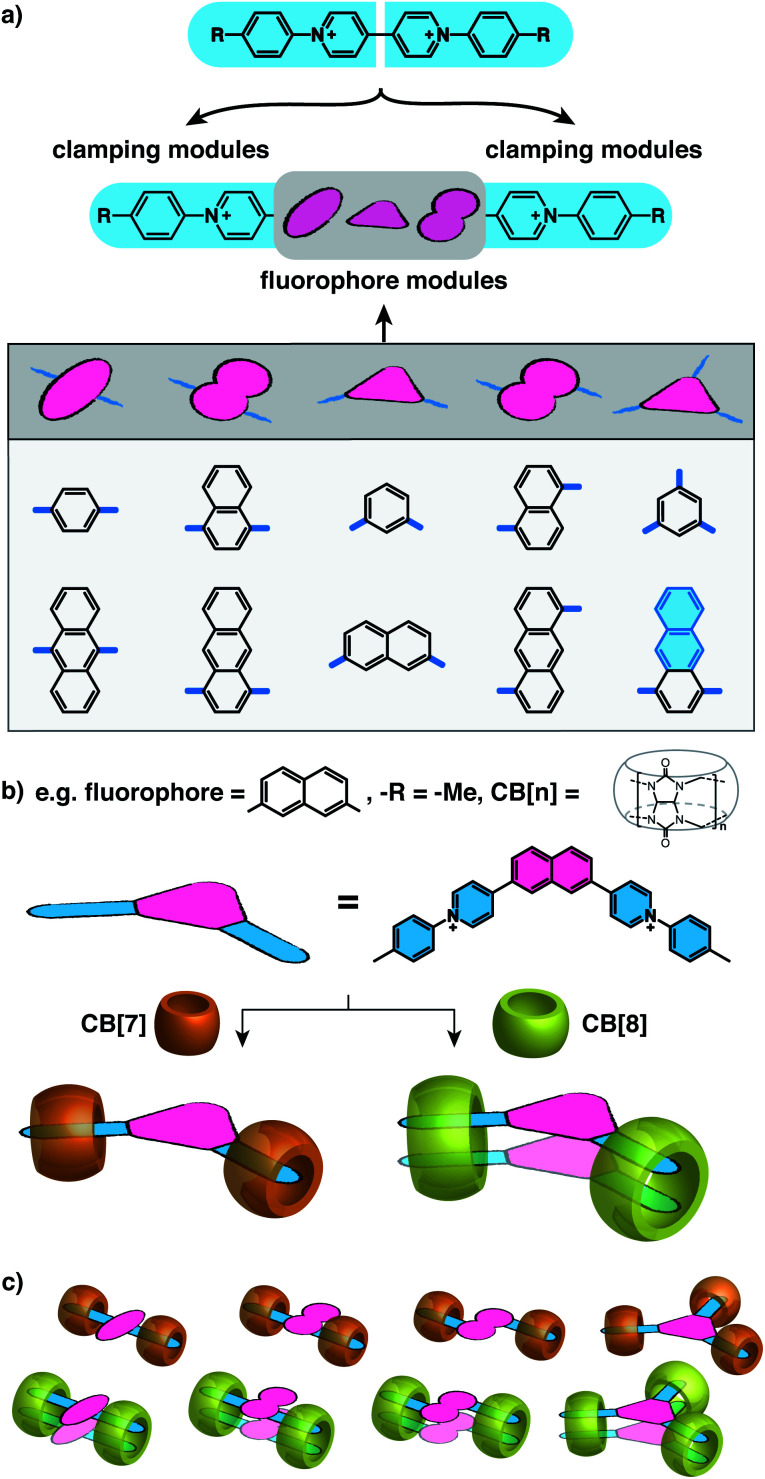
Modular strategy for designing fluorescent molecules (a) by plugging in a fluorophore module between two positively charged clamping modules, which herein are arylpyridinium moieties originated from diarylviologen derivatives. Following this strategy, (b) 2,7-naphthalene with non-parallel clamping modules, as an example, or (c) other fluorophores with various arrangement of clamping modules are expected to form a preorganized dimer constrained by CB[8]-mediated multiple clamping or a monomeric state of fluorophores protected by CB[7] from aggregation. The Cl^−^ counterions are omitted for clarity.

Recently, we have found that the dynamic exchange kinetics between the guests and CB[8] hosts are dramatically reduced through the formation of 2 : 2 quaternary complexes,^28^ in which two elongated guests such as diarylviologen derivatives are “clamped” in place by two CB[8] hosts into a multicomponent complex. The simultaneous formation of two ternary motifs within a discrete complex decreases the likelihood of dissociation compared to a typical ternary complex.^28^ The formation of 2 : 2 complexes opposed to elongated supramolecular polymers requires a small change in the conformational entropy during complexation, *i.e.* a molecule with significant rigidity.^28,29^ For instance, various rigid molecular moieties such as benzidine,^30^ benzothiazole,^31,32^ arylpyridinium,^28,33^ arylterpyridyl,^34^ bipyridinium,^35^ and benzimidazole^29^ have been employed to produce CB[8]-mediated 2 : 2 complexes. Herein, we present a general and modular strategy towards the dimerization of arbitrary functional components (fluorophores in this work) by connecting them to multiple rigid modules that can be “clamped” together by CB[8] complexation. We use arylpyridinium moieties as the rigid “clamping” module (Fig. 1a) and exploit the modular strategy for designing fluorescent complexes in water comprised of two fluorophores that are stacked in a specific configuration with a constraint applied by CB[8] macrocycles at multiple points.

As illustrated in Fig. 1a, water-soluble fluorescent molecules are designed to incorporate a fluorescent core between two positively charged clamping modules, which in this work are arylpyridinium motifs originating from previously studied diarylviologen derivatives.^28^ When one equivalent (equiv.) of CB[8] is added to the system with one equiv. of guest molecule, two clamping modules are expected to bring together two guest molecules yielding a 2 : 2 quaternary complex. The fluorophore cores from each guest molecule are brought to close proximity to each other as a consequence of the assembly, resulting in preorganized dimeric fluorophore stacking. The preorganized dimer complex is stabilized by multiple CB[8] clamps, which ensures interaction between fluorophores for a sufficiently long period of time, endowing the complex with emergent photophysical properties. As the fluorophore modules are not encapsulated by CB[8] (Fig. 1c), a variety of fluorophores, including those with sizes substantially larger than the CB[8] cavity, can be employed as functional cores in this modular strategy (Scheme S1†). Moreover, the photophysical properties of the resultant complexes can be readily customized through altering fluorophores as well as the clamping modules. As exemplified in Fig. 1b, dimeric stacking still occurs even when the two clamping modules are non-parallel to each other (separated by an angle < 180°). The flexibility offered by this modular design provides a molecular toolbox and platform in which a wide range of fluorophores can be readily studied in their discrete monomeric or dimeric states facilitating future investigations of quantum optical phenomena.

## 2 Results and discussion

Fluorescent molecules are designed by bridging two arylpyridinium motifs with a central fluorophore core. Nine phenyl, naphthyl, or anthracenyl homologues are investigated as the fluorophore cores in this study (Fig. 1 and Scheme S1†). The general synthesis (Scheme S2†) of the molecules starts with Suzuki–Miyaura cross-coupling^36^ of two pyridin-4-yl groups onto the fluorophore core, followed by transformation of the pyridin-4-yl groups into arylpyridinium salts through a Zincke reaction.^37–39^ A complete study was carried out on **Ant910Me**, which contains a 9,10-anthracenyl (“Ant910”) as the central core and *p*-tolyl pyridiniums (“Me”) as clamping modules (Fig. 1a), which is presented here as a typical case prior to a further general discussion.

Guest molecule (G) **Ant910Me** is found to form 1 : 2 complexes with CB[7], denoted G_1_–CB[7]_2_, and 2 : 2 complexes with CB[8], denoted G_2_–CB[8]_2_. The complex formations are verified by several NMR techniques including ^1^H NMR, 2D nuclear Overhauser spectroscopy (NOESY), and diffusion ordered spectroscopy (DOSY).

### 2.1 G_1_–CB[7]_2_: a discrete monomeric state

**Fig. 2:**
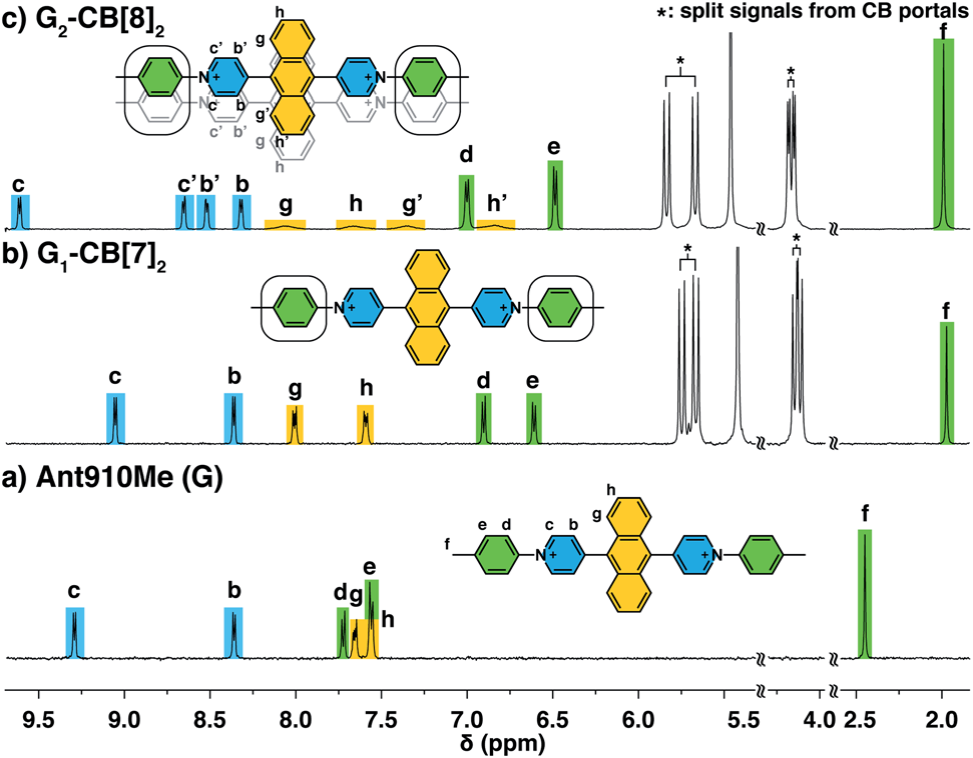
^1^H NMR spectra (500 MHz) of (a) **Ant910Me** (G), and (b) its complexation with 2 equiv. of CB[7] (G_1_–CB[7]_2_), or (c) with 1 equiv. of CB[8] (G_2_–CB[8]_2_) in D_2_O with a uniform guest concentration of 100 μM at 298 K. The Cl^−^ counterions are omitted for clarity.

The way in which CB[*n*] binds to guest molecules can be precisely probed by ^1^H NMR. Protons residing inside the CB cavity typically exhibit upfield chemical shifts of *ca.* 1 ppm; while protons located outside and proximate to the CB portals will display downfield shifts.^40^^1^H NMR spectra of **Ant910Me** (Fig. 2a) and its CB[7] complex (Fig. 2b) demonstrate significant upfield shift for the H^c,d,e,f^ upon complexation, which indicates that the entire tolyl moiety resides inside the CB[7] cavity along with a part of the pyridinium group. Meanwhile, a slight downfield shift of H^g,h^ confirms that the anthracenyl core is located outside the CB[7] portals.

CB proton signals ranging from 4 to 6 ppm split into two sets of equivalent doublets (Fig. 2b). The signal splitting suggests that the rate of CB[7] flipping around the tolyl moieties falls in the slow exchange limit with respect to the NMR time scale (500 MHz, 298 K). This slow flipping rate enables the direct observation of the two CB portals existing in distinctly different chemical environments.^22,28,39,41^ Signal splitting is observed throughout the titration of the guest into a CB[7] solution, leading to quantitative splitting at a ratio of 1 : 2, which confirms the stoichiometric formula of this CB[7] complex as G_1_–CB[7]_2_. Thus, each **Ant910Me** molecule is readily isolated in a monomeric state in aqueous solution when complexed by two CB[7] macrocycles.

### 2.2 G_2_–CB[8]_2_: preorganized π-stacked dimers

Upon titration of **Ant910Me** into a solution of CB[8] (Fig. 2c), splitting of the CB[8] protons are observed as well as the upfield shift of H^d,e,f^. Both observations suggest that the CB[8] molecules remain at the tolyl moieties, with a slow flipping rate and asymmetric portal environment. Careful analysis of the signal splitting and proton integration confirms a binding stoichiometry of “1 : 1”, therefore, this CB[8]-mediated complex contains an equal number of hosts and guests. As elaborated in a previous work,^28^ this complex cannot be a 1 : 1 binary complex as CB[8]-mediated binary complexes exhibit much faster dynamics. An elongated polymeric G_*n*_–CB[8]_*n*_ complex (*n* = 1,2,3…), fabricated from the sequential stacking of tolyl groups,^23^ is also not possible as the head-to-tail alignment of two tolyl groups would result in a symmetric portal environment contrary to the observed splitting. Therefore, the most probable binding mode is a 2 : 2 complex (G_2_–CB[8]_2_), as illustrated in Fig. 2c, where two fluorescent molecules are constrained to overlap with each other. In this binding mode, the tolyl groups are head-to-head, thus resulting in an asymmetric portal environment for each CB[8]. The observed slow flipping rate of CB[8] and the signal splitting is explained by the tightly filled CB[8] cavities as well as the electrostatic interactions between multiple positive charges on one side of the CB portals.

**Fig. 3:**
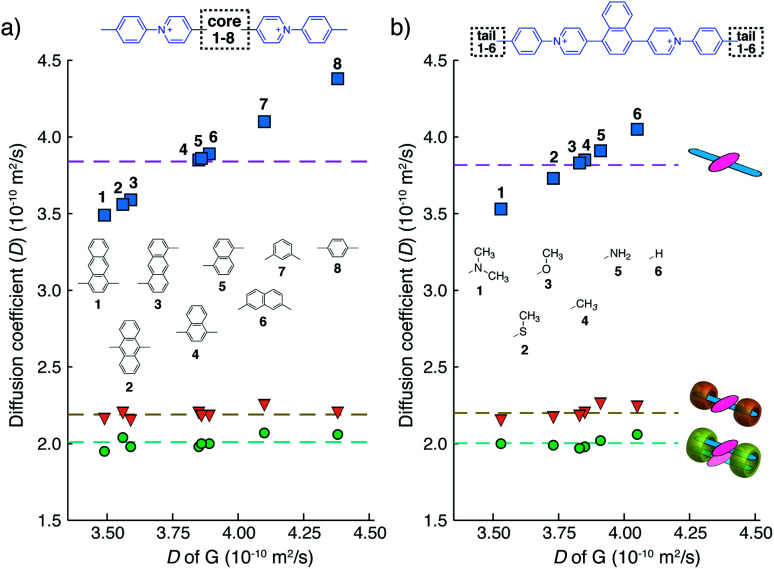
Diffusion coefficients obtained from DOSY NMR for G (rectangles), G_1_–CB[7]_2_ (triangles), and G_2_–CB[8]_2_ (circles) with variation in (a) fluorophore cores or (b) substituted tails. (D_2_O solution with a uniform guest concentration of 100 μM at 298 K.) The Cl^−^ counterions are omitted for clarity.

We have learned from previous works^28,30,39,41,42^ that the diffusion coefficient (*D*) of a CB[*n*]-mediated complex is primarily determined by the number of CB macrocycles existing in the complex. Therefore, the formation of G_2_–CB[8]_2_ is further confirmed through a semi-quantitative analysis of *D via* DOSY experiments. As shown in Fig. 3a and Table S1,†*D* values of unbound guests (G) in aqueous solution range from 3.49 to 4.38, showing a standard deviation (SD) of 0.3. A much narrower distribution is observed for CB[8]-mediated complexes, ranging from 1.95 to 2.07 with a SD of 0.04 (Fig. 3a). These *D* values are much smaller than that of free CB[8] (*D* = 3.11) and typical binary complexes such as dzpy_1_–CB[8]_1_ (*D* = 3.04).^41^ However, the *D* values measured here are almost the same as for the 2 : 2 complexes produced by diarylviologen derivatives^28,41^ such as (VNMe_2_)_2_–CB[8]_2_ (*D* = 2.01). Therefore, the DOSY data fully supports the formation of a G_2_–CB[8]_2_ complex involving the stacking of two fluorescent molecules held together by two CB[8] hosts.

The relative orientation of the two fluorophores with respect to each other is probed by proton and NOESY NMR in this dimeric system. As the proton spectrum recorded for G_2_–CB[8]_2_ (G = **Ant910Me**) exhibits more complicated signal splittings than that of G_1_–CB[7]_2_, COSY NMR (Fig. S2†) is used to identify each proton. Both the pyridinium and anthracenyl protons in this 2 : 2 complex split into two sets of equivalent peaks corresponding to H^b,b′,c,c′^ and H^g,g′,h,h′^ in Fig. 2c. The observation of two sets of signals suggests (i) a slow dynamic process and (ii) a certain asymmetry existing for the most probable configuration of the G_2_–CB[8]_2_ complex, which is consistent with a cofacial stacking and partial overlap of the two aromatic fluorophores as illustrated in Fig. 2c. Partial overlap of the two fluorophores with a slippage along their extended axis will result in one set of equivalent protons lying on top of or below an aromatic ring of the other molecule, while the other set of equivalent protons does not. The first set of equivalent protons are expected to display signals in a higher-field region on account of shielding by aromatic ring currents, compared to the latter set of equivalent protons, which is consistent with the observation of significantly lowered chemical shifts for H^g′,h′^ compared to H^g,h^. Similarly, the difference observed between H^c,b^ and H^c′,b′^ is interpreted as two sets of protons that reside in different shielding and deshielding environments arising from the CB[8] portal.

The partial overlap of two aromatic fluorophores is also supported by the cross-correlation signals observed in NOESY NMR, which reveals the relative position of protons located in space. Proton H^b′,c′^ (Fig. S3†), for instance, exhibits an intense cross-correlation with all anthracenyl signals (H^g,g′,h,h′^), whereas H^b,c^ can only “feel” protons that are closer to the pyridinium protons, *i.e.* H^g,g′^. This observation is consistent with the partial-overlap and stacking of the fluorophores where H^b′,c′^ rather than H^b,c^ are closer to H^h,h′^ in space (Fig. 2c and S3†).

### 2.3 Photophysics of the dimeric and monomeric **Ant910Me**

The well-resolved NMR spectrum of G_2_–CB[8]_2_ suggests that these complexes exist as discrete preorganized fluorescent dimers in aqueous solution without forming any larger aggregates. This is because the two CB[8] macrocycles mechanically block the interaction between multiple dimers. It also leads to a substantial change in the photophysical properties of **Ant910Me** upon complexation with CB[8] to G_2_–CB[8]_2_.

**Fig. 4:**
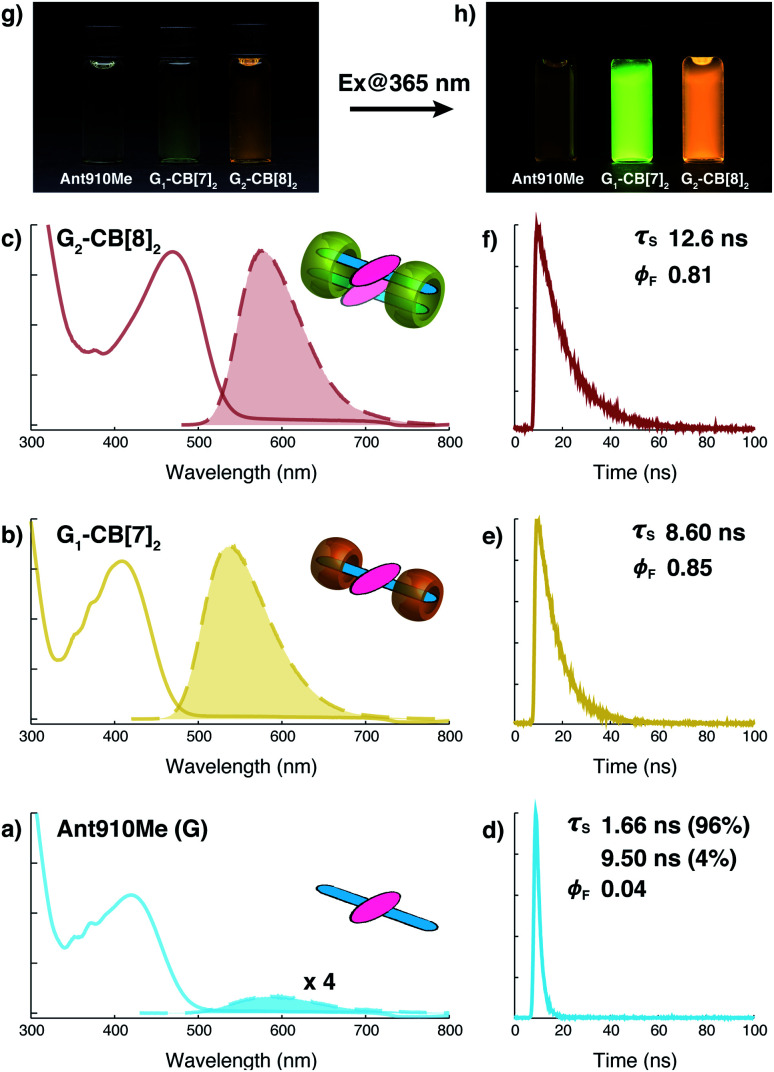
Steady-state absorption (solid line) and emission (dash line with filling color) spectra of (a) **Ant910Me** (G), and (b) its G_1_–CB[7]_2_ complex, and (c) G_2_–CB[8]_2_ complex, whose time-dependent fluorescence decay is displayed in (d), (e), (f), respectively, along with corresponding lifetime (*τ*_s_) and fluorescence quantum yield (*ϕ*_F_) results. Aqueous solution of each species with a uniform guest concentration of 15 μM is tested at 298 K. The intensity is not normalized but scaled up by the same factor except the emission of G which is enlarged by an additional 4 times for a clear vision. Quantified data can be found in Table 1. Photographs of each species with a guest concentration of 20 μM before (g) and after (h) photoexcitation at 365 nm.

As shown in Fig. 4a–c and Table 1, the anthracenyl moiety in G_2_–CB[8]_2_ exhibits a bathochromic shift of its absorption maximum (*λ*_abs_ = 469 nm) by over 50 nm compared to monomeric **Ant910Me** (*λ*_abs_ = 409 nm) in G_1_–CB[7]_2_ and unbound **Ant910Me** in pristine solution (*λ*_abs_ = 419 nm) (all compared at a concentration of 15 μM). The emission maximum of G_2_–CB[8]_2_ (*λ*_em_ = 578 nm) is also red-shifted relative to that of G_1_–CB[7]_2_ (*λ*_em_ = 537 nm), although **Ant910Me** in pristine solution exhibits the most bathochromic shift in emission (*λ*_em_ = 595 nm).

**Table 1:** Photophysical properties of studied fluorescent molecules and their CB[7]- or CB[8]-mediated complexes in aqueous solution at 298 K^a^

G	Species	*λ*_abs_/nm	*λ*_em_/nm	Δ*ν*/cm^−1^ (Δ*λ*/nm)	*τ*_s_/ns	*ϕ*_F_	*k*_nr_/μs^−1^	*k*_r_/μs^−1^	*ε*(*λ*_abs_)/10^3^ M^−1^ cm^−1^	*ε*(*λ*_abs_) × *ϕ*_F_/10^3^ M^−1^ cm^−1^
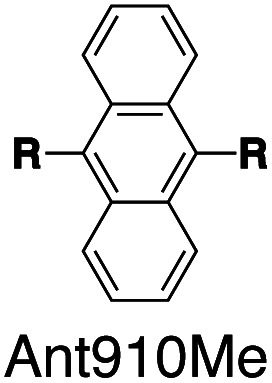	G	419	595	7060 (176)	1.66 (96%) 9.50 (4%)	0.04	486.4	20.3	9.1	0.4
	G_1_–CB[7]_2_	409	537	5828 (128)	8.60	0.85	17.4	98.8	12.1	10.3
	G_2_–CB[8]_2_	469	578	4021 (109)	12.6	0.81	15.1	64.3	13.3	10.8
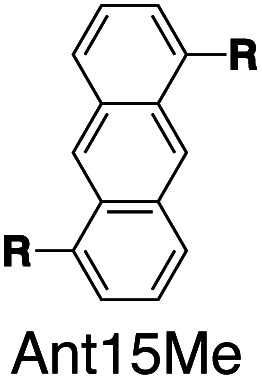	G	422	590	6748 (168)	2.13 (97%) 8.35 (3%)	0.12	379.9	51.8	14.8	1.8
	G_1_–CB[7]_2_	416	546	5723 (130)	7.97	0.82	22.6	102.9	17.5	14.4
	G_2_–CB[8]_2_	443	570	5030 (127)	12.3	0.82	14.6	66.7	17.3	14.2
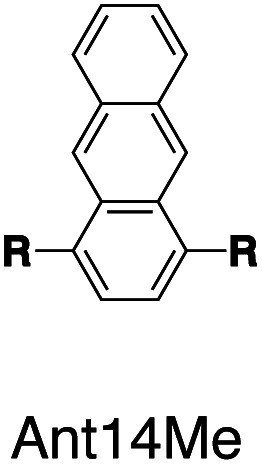	G	435	618	6807 (183)	4.46	0.21	177.1	47.1	11.3	2.4
	G_1_–CB[7]_2_	426	585	6380 (159)	8.74	0.60	45.8	68.6	13.8	8.3
	G_2_–CB[8]_2_	478	650	5536 (172)	1.60 (63%) 7.32 (37%)	0.02	263.7	5.4	13.0	0.3
	G_2_–CB[8]_3_	509	665	4609 (156)	1.73 (41%) 7.20 (59%)	0.01	199.7	2.0	11.1	0.1
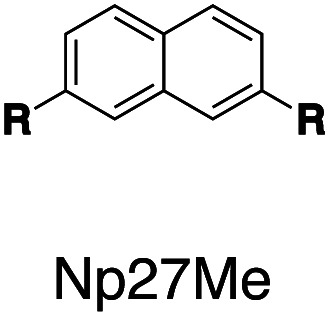	G	335	477	8886 (142)	8.85	0.96	4.5	108.5	52.1	50.0
	G_1_–CB[7]_2_	330	465	8798 (135)	2.99 (36%) 10.3 (64%)	0.94	7.8	122.6	57.5	54.0
	G_2_–CB[8]_2_	351	531	9658 (180)	36.8	0.55	12.2	14.9	62.0	34.1
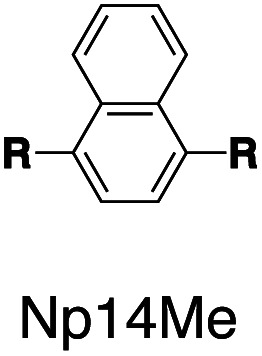	G	371	484	6293 (113)	4.34	1.00	0.0	230.4	20.0	20.0
	G_1_–CB[7]_2_	365	471	6166 (106)	3.25	0.96	12.3	295.4	23.9	23.0
	G_2_–CB[8]_2_	395	518	6011 (123)	7.09 (39%) 17.3 (61%)	0.95	3.8	71.3	27.0	25.6
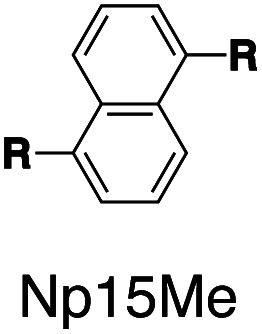	G	358	482	7186 (124)	5.66	0.94	10.6	166.1	23.6	22.2
	G_1_–CB[7]_2_	354	460	6509 (106)	3.20	0.92	25.0	287.5	26.4	24.3
	G_2_–CB[8]_2_	380	474	5219 (94)	5.22 (57%) 9.76 (43%)	0.92	11.2	128.3	30.4	28.0
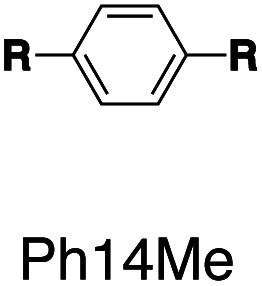	G	341	454	7300 (113)	1.97	0.77	116.8	390.9	54.4	41.9
	G_1_–CB[7]_2_	338	419	5719 (81)	1.36	0.88	88.2	647.1	60.5	53.2
	G_2_–CB[8]_2_	358	472	6747 (114)	2.68 (28%) 10.7 (72%)	0.82	21.3	97.0	59.0	48.4
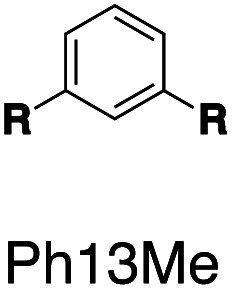	G	314	431	8645 (117)	2.36	0.63	156.8	266.9	35.9	22.6
	G_1_–CB[7]_2_	311	415	8058 (104)	1.25 (21%) 2.66 (79%)	1.00	0.0	423.0	37.2	37.2
	G_2_–CB[8]_2_	330	446	7882 (116)	13.3	0.84	12.0	63.2	37.7	31.6
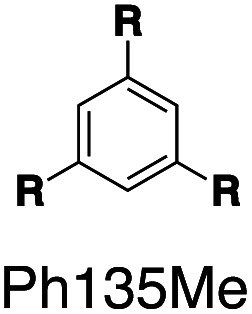	G	314	441	9171 (127)	2.20	0.71	131.8	322.7	40.6	28.8
	G_1_–CB[7]_3_	311	417	8174 (106)	2.37	0.96	16.9	405.1	40.7	39.0
	G_2_–CB[8]_3_	331	469	8890 (138)	14.6	0.27	50.0	18.5	40.5	10.9

a Δ*ν*: wavenumber difference between *λ*_abs_ and *λ*_em_ (“Stokes shift”); *ε*(*λ*_abs_): molar absorption coefficient; *ϕ*_F_: fluorescence quantum yield; *ε*(*λ*_abs_) × *ϕ*_F_: emission brightness.

After photoexcitation, fluorescence decay as well as the corresponding lifetime are recorded from time-correlated single photon counting (TCSPC) experiments, Fig. 4d–f. Excited anthracenyl dimers in the G_2_–CB[8]_2_ complexes display an excimer-like state that exhibits a lifetime (*τ*_s_) of 12.6 ns, which is much longer than *τ*_s_ of 8.6 ns for its monomeric counterpart in G_1_–CB[7]_2_. A biexponential decay is observed for **Ant910Me** in pristine solution measured at the same concentration (15 μM) as that in G_1_–CB[7]_2_ and G_2_–CB[8]_2_, showing 96% of the intensity is due to a short-lived component of 1.66 ns and 4% arising from a long-lived component of 9.50 ns.

Fluorescence quantum yields (*ϕ*_F_) for each species are measured by an absolute method using an integrating sphere. The emission of **Ant910Me** is significantly quenched in a pristine solution with a *ϕ*_F_ of 0.04, whereas its fluorescence intensity is dramatically enhanced upon complexation with either CB[7] or CB[8], showing a *ϕ*_F_ of 0.85 for G_1_–CB[7]_2_ and a *ϕ*_F_ of 0.81 for G_2_–CB[8]_2_ (Table 1).

The negligible quantum yield, bimodal decay, and red-shifted emission in a pristine solution of **Ant910Me** all suggest a certain extent of aggregation in aqueous solution, whose photophysical properties are highly concentration-dependent. On the other hand, complexation with CB[7] or CB[8] ensures a dispersion of discrete fluorophores in solution in either monomeric or dimeric fashion, respectively. It is known that the polarity of CB cavities is lower than that of water, which will also affect photophysical properties of dye molecules.^19,43^ Therefore, in the following discussion, a comparison is made between CB[7]- and CB[8]-mediated complexes on account of their similar cavity polarities. A comparison between G_1_–CB[7]_2_ and G_2_–CB[8]_2_ of **Ant910Me** shows that the stacking of anthracenyl moieties as a dimer, relative to monomer, exhibits (1) a significant bathochromic shift in absorption and emission, (2) an elongated excited-state lifetime, and (3) comparably high fluorescence efficiency.

### 2.4 Applicability and flexibility of the modular strategy

Although individual cases have demonstrated photophysical changes upon complexation with CB[8],^31,35,39,44–47^ the beauty and power of this work stems from the simple modular design. As illustrated in Fig. 1a, any selected fluorophore can be readily inserted between clamping modules, resulting in its monomeric or dimeric species through complexation with CB[7] or CB[8], respectively.

Following this design strategy, a further eight fluorescent molecules were successfully synthesized, with similar topology to **Ant910Me** but with systematic variation in their structures. For example, the fluorophore cores are augmented between phenyl, naphthyl, and anthracenyl. Alternatively, the alignment between the two clamping modules is altered. While several derivatives exhibit both clamping modules in-line with one another (**Ph14Me**, **Np14Me**, **Ant910Me**, and **Ant14Me**) others have clamping modules that are not in-line but remain parallel to each other such as **Np15Me** and **Ant15Me**, or are no longer aligned in a parallel manner but with an angle < 180° (**Ph13Me** and **Np27Me**). Finally, one can readily add additional clamping motifs around the fluorophore core moiety, as demonstrated in the triply clamped systems **Ph135Me** and **Ant14Me**.

Results from ^1^H NMR and DOSY, as shown in Fig. 3a and in the ESI (Table S1 and Fig. S1–S17†), demonstrates that these fluorescent molecules all perform in a manner similar to **Ant910Me**. Despite their structural variation, they all generate a monomeric fluorophore in the presence of CB[7] and dimeric stacking of fluorophores with CB[8]. As CB[7] and CB[8] only bind the clamping modules (*i.e.* tolyl pyridinium moieties), choice of the fluorophores is no longer limited by the size and shape of the macrocycle cavities. Large fluorophores such as anthracenyl derivatives, which to date have only been shown to complex CB[7] or CB[8] along their principal symmetry axis, are easily incorporated using this strategy regardless of their substitution pattern. Moreover, small aromatic rings like phenyl moieties, whose binding is extremely dynamic inside a single CB[8] cavity, are now readily immobilized and constrained within a 2 : 2 complex.

### 2.5 Photophysical properties

In terms of photophysical properties, most fluorescent molecules also behave similarly to **Ant910Me**, with the exception of a few outliers that are discussed later in detail.

**Fig. 5:**
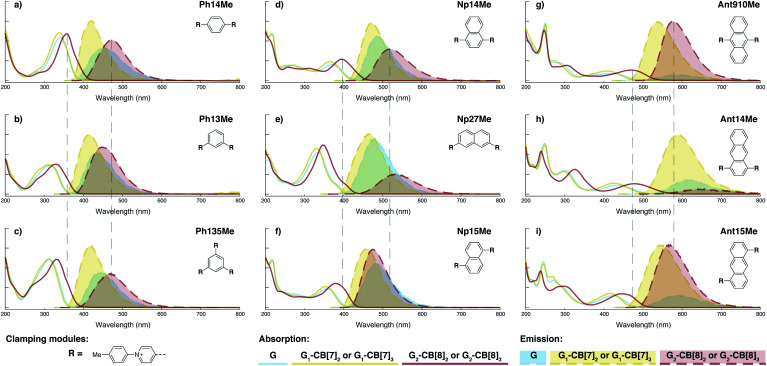
Steady-state absorption (solid line) and emission (dash line with filling color) spectra of non-associated fluorescent molecules (G in blue), and their CB[7]-mediated complexes (G_1_–CB[7]_2_ or G_1_–CB[7]_3_ in yellow) as well as CB[8] mediated complexes (G_2_–CB[8]_2_ or G_2_–CB[8]_3_ in red). The study covers the derivatives of three types of fluorophores including phenyl such as (a) **Ph14Me**, (b) **Ph13Me**, (c) **Ph135Me**, naphthyl such as (d) **Np14Me**, (e) **Np27Me**, (f) **Np15Me**, and anthracenyl such as (g) **Ant910Me**, (h) **Ant14Me**, (i) **Ant15Me**. Aqueous solutions of each species are tested under a uniform guest concentration at 298 K. The intensity is not normalized but scaled up by the same factor in most cases. Quantified data can be found in Table 1.

Preorganized ground-state dimers are readily produced by the formation of G_2_–CB[8]_2_ in aqueous solution, corresponding to a considerable bathochromic shift in the absorption band (Fig. 5 and Table 1). An excimer-like emission with a broadened and structureless profile is observed for all fluorophores in their G_2_–CB[8]_2_ systems, exhibiting a red-shift in their emission maximum relative to their monomeric form in G_1_–CB[7]_2_ systems. Solutions of G_2_–CB[8]_2_ compared to their G_1_–CB[7]_2_ counterparts exhibit a smaller rate constant for non-radiative deactivation (*k*_nr_), which corresponds to their observed elongated excited-state lifetime as well as comparably high quantum yields. Molar absorption coefficients for all fluorophores are slightly increased upon complexation with either CB[7] or CB[8] (Table 1) along with their high quantum yield, leading to reasonably high brightness (*ε* × *ϕ*_F_) in aqueous solution.^48^ Considering their long fluorescence lifetimes, G_2_–CB[8]_2_ complexes in general should be promising candidates for time-gated imaging for biological systems.

Fluorescent molecules that contain anthracenyl cores all exhibit a low quantum yield in solution with a lifetime much shorter than that in their CB[7]- or CB[8]-mediated complexes. The discrete monomeric species of **Ant910Me**, **Ant15Me**, and **Ant14Me** in their corresponding G_1_–CB[7]_2_ complexes all display a lifetime around 8 ns (Table 1), similar to the lifetime (*τ*_s_) of 9,10-diphenylanthracene (DPA), a typical anthracenyl standard.^49^ This recovery of lifetime to a value similar to DPA implies that the fluorescence of these CB[7] complexes is mainly contributed by their anthracenyl cores. In a solution of only free molecules (without the presence of CB), the excited anthracenyl cores are deactivated through certain pathways as evidence from the observed quenching of fluorescence. In particular, a typical deactivation pathway would be the photoinduced electron transfer (PET) from the anthracenyl core to π-deficient pyridinium moieties.^50,51^ However, the significant recovery of emission after complexation suggests that these deactivation pathways are forbidden or are at least largely restricted in both the G_1_–CB[7]_2_ and G_2_–CB[8]_2_ complexes. Quantum yields of the naphthyl and phenyl species are generally large (0.9–1.0 for Np, 0.6–1.0 for Ph) contrary to anthracenyl analogues, regardless of complexation, implying that PET from these two fluorophore cores to pyridinium moieties is not efficient.

Systematic variation in the alignment between the clamping modules also affects their photophysical properties. **Np15Me** and **Ant15Me**, with two parallel clamping modules that are not aligned, exhibit a red-shift in emission, which is not as large as for other species (Fig. 5) upon forming G_2_–CB[8]_2_ complexes. This non-aligned connectivity may force the two fluorophores to stack in a less *J* aggregate-like fashion.^52^ When the clamping modules are non-parallel, the G_2_–CB[8]_2_ of **Np27Me** displays a quantum yield of 0.55, which is almost half the value of the other naphthyl homologues (Table 1). However, this species exhibits a distinctively long-lived excited state with a *τ*_s_ up to 37 ns. Similar results are observed in CB[8]-mediated complexes of **Ph13Me**, which also possesses non-parallel clamping modules. The reduced fluorescence efficiency along with the elongated lifetime suggests that dimeric stacking in species with non-parallel clamping units may significantly suppress radiative pathways (*i.e.* see reduced *k*_r_ values in Table 1).

### 2.6 Triple clamping

**Ph135Me** is a more complex version of non-parallel clamping, which forms dimeric stacks through triple clamping, denoted G_2_–CB[8]_3_ (Fig. S16†). Triple non-parallel clamping leads to a further decrease in *k*_r_ (Table 1) compared to G_2_–CB[8]_2_ of **Ph13Me**, which results in a reduced quantum yield (0.27) of G_2_–CB[8]_3_ that is about one third of that of its CB[7]-mediated complex, G_1_–CB[7]_3_. Besides suppressing the radiative pathway, triple clamping exhibits a concerted feature of multivalency^53^ further stabilizing the dimeric stacking of two phenyl moieties. In a mixture consisting of 4 equiv. of **Ph135Me** and 3 equiv. of CB[8], excess guest molecule does not result in statistical complexes such as G_2_–CB[8]_2_ and G_2_–CB[8]_1_ (Fig. S17†). Instead, **Ph135Me** molecules exist either as G_2_–CB[8]_3_ complexes or as a free guest in aqueous solution.

In addition to **Ph135Me**, which has three uniform clamping modules, **Ant14Me** with a protruding fluorophore core is also able to form a G_2_–CB[8]_3_ complex. Isothermal titration calorimetry and UV-Vis titration both confirm a binding stoichiometry of 2 : 3 (Fig. S12†). Its diffusion coefficient from DOSY NMR gives a *D* value similar to that of Ph135Me_2_–CB[8]_3_ (Table S1, Fig. S11 and S16†). Considering its T-shape topology, a third CB[8] in the **Ant14Me** G_2_–CB[8]_3_ complex binds with the two protruding, stacked anthracenyl cores. However, in contrast to **Ph135Me**, CB[8] complexation of **Ant14Me** (in excess) does not exhibit a self-sorting behavior. Addition of extra **Ant14Me** guest molecules gradually transforms the solution of G_2_–CB[8]_3_ into 2 : 2 complexes, in which two CB[8] macrocycles are bound with the two tolyl pyridinium moieties rather than the protruding anthracenyl cores (Fig. S10–S12†). This suggests that the affinity of CB[8] around the protruded binding site is substantially weaker than its binding with the clamping modules, which is confirmed by the ITC result in Fig. S12.†

### 2.7 Restricted intracomplex motion

Dimeric fluorophore stacking in G_2_–CB[8]_2_ complexes generally exhibit an enhanced fluorescence efficiency, particularly in the case of employing anthracenyl motifs as cores. This observation implies that motion within the complex (intracomplex motion) of G_2_–CB[8]_2_ is extremely retarded and restricted, thus effectively suppressing deactivation pathways.

#### 2.7.1 Interconversion dynamics quantified by VT-NMR

The NOESY spectrum of G_2_–CB[8]_2_ (Fig. S3†) shows that cross-correlations between H^g^ and H^g′^ as well as those between H^h^ and H^h′^ are much more intense than correlations caused by ^3^*J*_H–H_ coupling for H^g^–H^h^ and H^g′^–H^h′^. As chemical exchange also contributes to NOESY signals, this observation implies the presence of a dynamic interconversion between two discrete states within the CB[8] complex. This is also the reason why anthracenyl and pyridinium proton signals in G_2_–CB[8]_2_ split into two sets of equally intense peaks (Fig. 2c) that are not observed in G_1_–CB[7]_2_ (Fig. 2b).

**Fig. 6:**
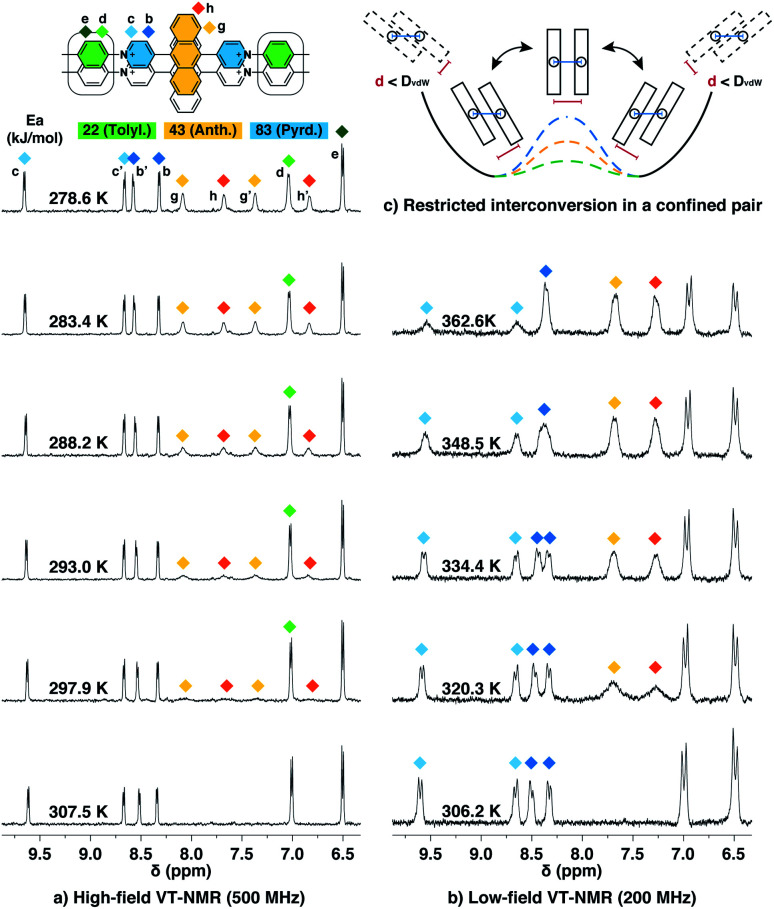
Variable-temperature ^1^H NMR spectra of the Ant910Me_2_–CB[8]_2_ complex in D_2_O solution (a) with a temperature increased from 278.6 K to 307.5 K (top to bottom) recorded by high-field spectrometer (500 MHz), and (b) with a temperature increased from 306.2 K to 362.6 K (bottom to top) recorded by low-field spectrometer (200 MHz), showing (c) restricted intracomplex rotations of tolyl, anthracenyl, and pyridinium pairs with non-uniform activation energies (*E*_a_) of 22 kJ mol^−1^, 43 kJ mol^−1^, and 83 kJ mol^−1^, respectively, which are analysed from the temperature-dependent line-broadening (Fig. S19†). The temperature of 500 MHz and 200 MHz spectrometers is calibrated by MeOD (D, 99.8%) and ethylene glycol (80% in DMSO-d_6_), respectively. The Cl^−^ counterions are omitted for clarity.

Interconversion between the two states is further confirmed and quantified by variable-temperature nuclear magnetic resonance spectroscopy (VT-NMR). As shown in Fig. 6a, four signals of H^g,g′,h,h′^ that correspond to two stacked anthracenyl cores broaden equally until coalescence is observed as the temperature rises from 278.6 K to 307.5 K on a high-field NMR spectrometer (500 MHz). A subsequent increase of temperature from 306.2 K to 362.6 K on a low-field NMR spectrometer (200 MHz) (Fig. 6b) leads to a gradual merging of the four signals into two broad peaks, which later become sharper as the temperature increases. The transition of the H^g,g′,h,h′^ signals from the slow exchange limit to the fast exchange limit confirms the existence of a dynamic interconversion between two discrete states for the anthracenyl pair. By analysing the temperature-dependent line-broadening in the slow exchange limit^54–56^ (Fig. S19†), an activation energy of 43 kJ mol^−1^ is obtained for this interconversion.

As lowering the magnetic field is equivalent to severely heating the sample, the switch of VT-NMR from high-field to low-field enables us to witness and quantify a very slow exchange process such as that for the pyridinium pair in this study. In the spectra recorded by the high-field spectrometer (Fig. 6a), no significant line broadening is observed for pyridinium signals (H^b,b′,c,c′^), whereas in the low-field VT-NMR, the signal broadening corresponding to an exchange in the slow limit is readily observed upon increase in temperature (Fig. 6b). The temperature-dependent signal broadening suggests an activation energy as large as 83 kJ mol^−1^ (Fig. S19†), implying a relatively slow interconversion of the pyridinium pair within the complex. The interconversion of protons in the stacked tolyl pair is already displayed in the fast exchange limit as demonstrated by the line sharpening of the H^d^ signal as the temperature is increased in the high-field spectrometer, exhibiting a relatively small exchange barrier of 22 kJ mol^−1^ (Fig. 6a and S19†).

#### 2.7.2 Intracomplex motion restricted in constrained dimers

Despite the covalent bonds between the anthracenyl, pyridinium, and tolyl moieties, different interconversion barriers are observed from VT-NMR indicating three separate dynamic processes. Therefore, these three distinct processes cannot be attributed to either the back and forth shuffling of the two fluorophores along the long axis of the complex or to the complexation/decomplexation process with CB[8] because these two processes require a simultaneous movement of all components at the same rate yielding uniform activation energies. Moreover, complexation/decomplexation must be slower than all three dynamic processes observed. This indicates that the G_2_–CB[8]_2_ complex is fairly “static” in aqueous solution and must remain complexed longer than the dynamics for pyridinium interconversion, which is around 30 ms at room temperature.

Molecular dynamic (MD) simulations of the Ant910Me_2_–CB[8]_2_ complex in a cubic water box with 4000 H_2_O molecules was carried out in order to evaluate the stability of this complex under ambient conditions (298 K, 1 atm). The simulations indicate that the Ant910Me_2_–CB[8]_2_ complex (ESI† video media) remains as a single entity during the whole simulation period (>200 ns) without decomplexation or significantly altering its structure. This result is consistent with the analysis by NMR, which shows that the dimeric stack of fluorophores is constrained and stabilized by the CB[8]-mediated dual clamping. Moreover, the two stacked aromatic moieties, such as anthracenyl units, partially overlap one another and simultaneously rotate in a slow but coherent fashion during the MD simulation. For example, the two anthracenyl units (yellow) of the Ant910Me_2_–CB[8]_2_ complex in Fig. 6 must rotate or swing around the central axis of the complex in a coupled manner, which we refer to here as intracomplex motion. Thus, the activation energy obtained represents the energy barrier for each intracomplex rotation.

**Fig. 7:**
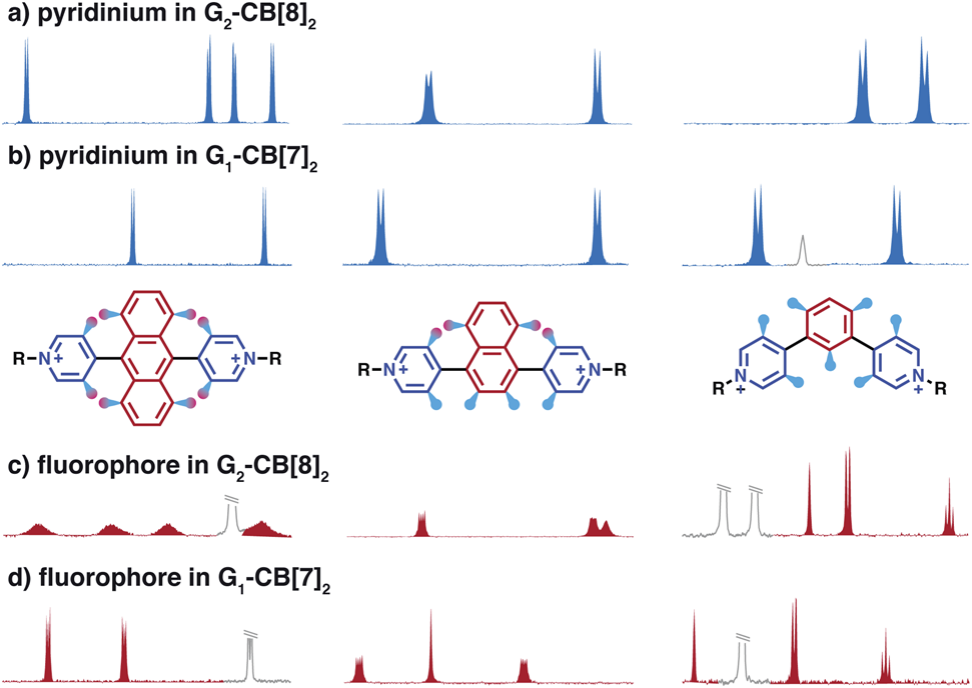
Linewidth comparison between G_2_–CB[8]_2_ and G_1_–CB[7]_2_ complexes for fluorescent molecules (**Ant910Me**, **Np14Me**, **Ph13Me**) with a variation in the extent of proton exclusion. The difference in steric hindrance of each fluorescent molecule is readily reflected by the signal broadening or splitting observed from both fluorophore and pyridinium moieties in G_2_–CB[8]_2_, but cannot be distinguished by those in G_1_–CB[7]_2_, indicating that the steric hindrance is largely amplified in a dimeric stacked complex.

The height of the energy barrier reflects the steric hindrance present around the “rotor”. As exemplified by **Ant910Me** in Fig. 7, the rotation of the anthracenyl group is hindered by the presence of several pairs of adjacent protons between the anthracenyl core and pyridinium units. The rotation of the pyridinium moieties, in addition to steric hindrance from the anthracenyl core, are also impeded by the CB[8] portals, thus showing the highest rotational barrier. On the other hand, rotation of the tolyl groups is not significantly influenced by CB portals as they mainly reside within the CB[8] cavities. Their motion is also not significantly retarded by neighboring pyridinium protons, which present less steric clash than those between the anthracenyl and pyridinium units. Therefore, the tolyl groups exhibit the lowest rotational barrier and their proton signals always fall within the fast exchange limit (Fig. 6). It is worth noting that “rotation” does not necessarily refer to a full rotation. In the case of **Ant910Me**, it is more likely that the two anthracenyl groups swing coherently within a limited angle on account of van der Waals repulsion (Fig. 6c), where the activation energy represents the steric hindrance for swinging between two degenerate states.

#### 2.7.3 Steric hindrance amplified in G_2_–CB[8]_2_ complexes

It is worth highlighting that steric hindrance between adjacent aromatic moieties is significantly amplified in the stacked dimers (G_2_–CB[8]_2_), which exhibit substantially slower dynamics than their monomeric counterparts (G_1_–CB[7]_2_). As shown in Fig. 7, the fluorophore and pyridinium moieties within the three molecules **Ant910Me**, **Np14Me**, and **Ph13Me** should experience a different degree of steric hindrance consistent with the number of clashing, neighboring protons. However, this difference is not observed in their monomeric forms (G_1_–CB[7]_2_) where proton signals attributed to pyridinium (Fig. 7b) and fluorophore units (Fig. 7d) are all sharp and are not split indicative of dynamics within the fast exchange limit.

In contrast, the dimeric complexes of these molecules (G_2_–CB[8]_2_) display significant differences in both their fluorophore and pyridinium components in their ^1^H NMR spectra. As shown in Fig. 7a and c, proton signals of **Ph13Me** exhibit a narrow linewidth in the fast exchange limit for both the pyridinium and 1,3-phenyl groups, which is consistent with the fact that no severe steric clash exists in this molecule. However, signal broadening occurs with an increase of steric repulsion in the dimeric complex of **Np14Me**. Furthermore, proton signals from the anthracenyl and pyridinium groups in the dimeric complex of **Ant910Me** both fall into a slow exchange limit and split into two sets of peaks, which corresponds to much slower intracomplex motions. This observation stems from a further increase in steric hindrance and is amplified for the G_2_–CB[8]_2_ complexes as rotation of one moiety is not only retarded by covalently linked “neighbours” but also hindered by adjacent groups on the other stacked molecule. Careful comparison between the monomeric and dimeric systems verifies that formation of a constrained system largely restricts and slows down intracomplex motions in these dimers.

### 2.8 Ground and excited states of π-stacked dimers

#### 2.8.1 Preorganized π-stacked ground-state dimer

The characteristic red-shift in emission and elongated excited-state lifetime (Fig. 4c and Table 1) suggest the formation of an excimer-like state for G_2_–CB[8]_2_ upon photoexcitation. However, the formation of the excimer-like state in G_2_–CB[8]_2_ complexes is quite different from those formed by pyrene derivatives or covalently linked pseudo-dimers.^2,48,57^ In such cases, the generation and decay of an excimer or excimer-like state involves the excitation of one single fluorophore followed by a diffusion-controlled interaction with a second ground-state fluorophore and ends up with relaxation towards the ground state.^58^ Therefore, the absorption band is often similar to that of a monomeric fluorophore as the excitation is firstly applied to a single molecule.^2^

In the case of G_2_–CB[8]_2_ (Fig. 5), however, a considerable bathochromic shift is generally observed in its steady-state absorption spectrum. Particularly, the vibronic progression is absent in the absorption of Ant910Me_2_–CB[8]_2_ (Fig. 4c) indicating a strong coupling and effective delocalization of π-electrons between the dimeric anthracenyl moieties at their ground states. This preorganized π-stacked ground-state dimer is excited as a precoupled entity to an excimer-like state, which is different from an excited monomer and, more importantly, does not require an additional diffusion-controlled process after photoexcitation. On the other hand, the excited G_2_–CB[8]_2_ complex will not exhibit an energy dissipation as significantly as during the formation of conventional excimers. This explains why **Ant910Me** in the G_2_–CB[8]_2_ complex exhibits a Stokes shift (wavenumber difference between *λ*_abs_ and *λ*_em_) of 4012 cm^−1^ (109 nm) smaller than the value of 5828 cm^−1^ (128 nm) in its G_1_–CB[7]_2_ complex (Table 1). Due to the absence of diffusion-controlled steps in their excited state, one expects a mono-exponential fluorescence decay at pico- and nano-second timescale for G_2_–CB[8]_2_ complexes after photoexcitation, contrary to the bimodal decay of conventional excimers.^58^

#### 2.8.2 Mono-exponential decay of excited G_2_–CB[8]_2_

A mono-exponential fluorescence decay is indeed observed for the Ant910Me_2_–CB[8]_2_ complex in TCSPC measurements (Fig. 4 and Table 1) and is further validated by time-resolved spectroscopies.

**Fig. 8:**
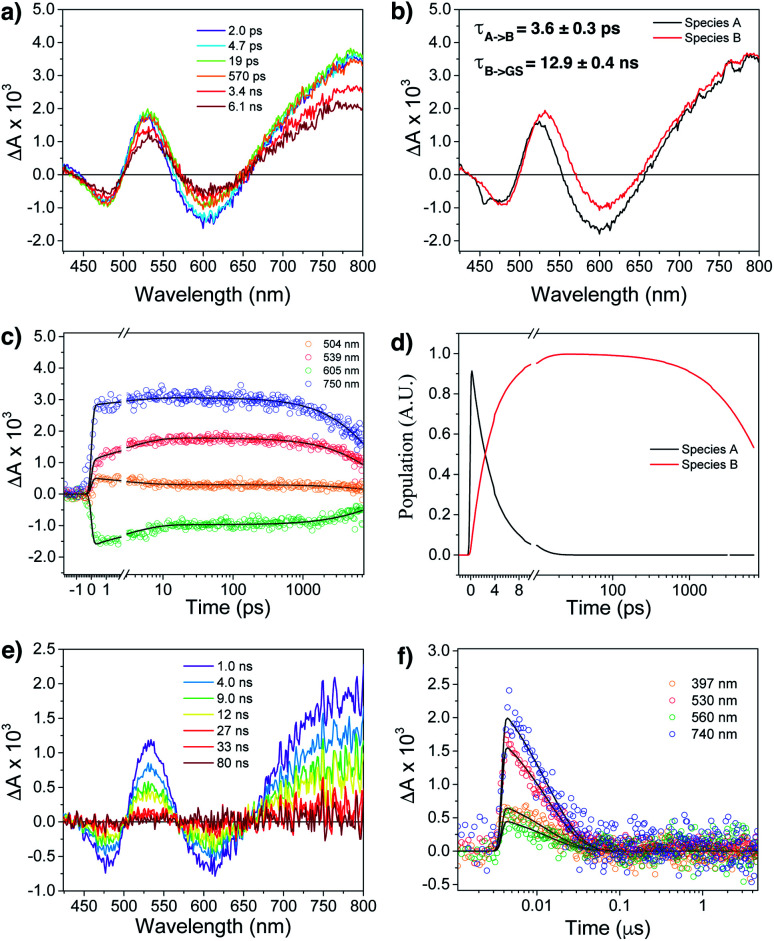
Femtosecond transient absorption spectra (fsTA) of (a) Ant910Me_2_–CB[8]_2_ complexes in D_2_O at 298 K following a laser pulse of 414 nm (1 μJ per pulse). Fitting from the raw data using a kinetic model A → B → GS gives (b) species associated spectra, (c) wavelength fitting, and (d) model population kinetics. (e) Nanosecond transient absorption spectra (nsTA) of species B and (f) its kinetic fitting at selected wavelength using mono-exponential model.

Femtosecond (fsTA) and nanosecond (nsTA) transient absorption were employed to monitor the dynamic relaxation of both G_2_–CB[8]_2_ and G_1_–CB[7]_2_ complexes of **Ant910Me** after photoexcitation (Fig. S20–S25, Table S2†). As shown in Fig. 8a and b, two species are clearly detected in the excited state from fsTA for Ant910Me_2_–CB[8]_2_. Both exhibit a spectral feature of ground state bleaching (GSB) from 431 nm to 498 nm overlapping with an excited state absorption (ESA) from 431 nm to 800 nm and a stimulated emission (SE) from 573 nm to 654 nm. Upon photoexcitation, the first species A relaxes to species B with a fairly short lifetime of 3.6 ± 0.3 ps (Fig. 8c, d and S21†), and then back to its ground state with a lifetime of 12.9 ± 0.4 ns (Fig. 8e and f), consistent with the value of 12.6 ns measured from TCSPC (Table 1). Species B exhibits a similar ESA profile as species A except a slight red-shift in its absorption maximum (Fig. 8b), which suggests that the evolution from A to B with a picosecond time constant probably corresponds to excited state solvation.^59^ In addition to solvation, the excited complex relaxes back to its ground state in a mono-exponential manner without observing other competitive pathways. It is worth mentioning that the excited state absorption spectra of Ant910Me_2_–CB[8]_2_ (Fig. 8a) are quite broad suggesting a strong coupling also existing in the excited states.

Ant910Me_1_–CB[7]_2_ after photoexcitation (Fig. S23†) exhibits an absorption maximum at around 475 nm in its ESA profile, which is much smaller than that of Ant910Me_2_–CB[8]_2_ at around 530 nm (Fig. 8b). This observation confirms that G_2_–CB[8]_2_ complexes are directly pumped up to the excited state of precoupled dimers rather than the excited state of monomers. It is worth noticing that several species are detected (Fig. S23 and S24†) for Ant910Me_1_–CB[7]_2_ upon photoexcitation, which also includes a small amount of long-lived species with a time constant of 6 ± 1 μs. This long-lived species should come from a triplet state, as its lifetime increases significantly (>340 μs, Fig. S25†) after removal of oxygen from the solvent. The observed rich dynamic processes implies that after photoexcitation the fluorescent molecule within the G_1_–CB[7]_2_ complex has sufficient structural freedom to relax to various low-energy excited states. On the other hand, the singular excited state dynamic observed for G_2_–CB[8]_2_ complex suggests a restricted or retarded structural change even in its excited state, which may affect both the radiative and non-radiative pathways.

#### 2.8.3 Enhanced fluorescence efficiencies from constrained and discrete excited dimers

The rate constants corresponding to non-radiative (*k*_nr_) and radiative (*k*_r_) pathways are readily calculated from excited-state lifetime (*τ*_s_) and quantum yield (*ϕ*_F_) values. As shown in Table 1, the formation of preorganized G_2_–CB[8]_2_ dimers always results in a reduced radiative rate constant *k*_r_ corresponding to a long-lived excimer-like excited state, which is smaller than the *k*_r_ values for the corresponding G_1_–CB[7]_2_ complexes. Contrary to typical excimers that lead to quenched emission,^58^ G_2_–CB[8]_2_ complexes maintain high fluorescence efficiencies on account of their substantial reduction in non-radiative rate constants *k*_nr_. This unique feature is attributed to a significant suppression of non-radiative deactivation through the formation of a preorganized dimer in G_2_–CB[8]_2_, which strongly restricts intracomplex motions as demonstrated above.

In addition to constrained complexation, the discrete nature of fluorophore dimers is also crucial to ensure high-efficiency fluorescence.^60,61^ The two CB[8] macrocycles that hold the fluorophore dimer together will mechanically block interactions from other dimers in aqueous solution, which effectively avoids the generation of dark excited states caused by arbitrary aggregation.

#### 2.8.4 Comparison with other dimeric systems

An advantage of forming preorganized π-stacked ground-state dimers is that the excitation wavelength for the system is shifted towards the visible region (*e.g.* 469 nm for the G_2_–CB[8]_2_ complex of **Ant910Me**), which is crucial for non-destructive imaging of biological systems. Importantly, the formation of a preorganized π-stacked ground-state dimer is not necessarily the same as bringing together two fluorophores into spatial proximity. For instance, a red-shift in the absorption band was not observed in previous reports where two fluorophores have been covalently linked together in close proximity.^2,48,57^ The preorganization of π-stacked dimers of anthracene and its derivatives through non-covalent methods have been previously realized in rigid media containing small discrete cavities, such as crystalline lattices^60–63^ and supramolecular capsules,^64^ suggesting that the formation of a preformed π-stacked dimer requires strict spatial confinement in order to: (1) isolate each dimer as a discrete entity, (2) maintain a specific π-stacked configuration, and (3) restrict interplanar spacing between the two fluorophores.

The spontaneously assembled G_2_–CB[8]_2_ complex satisfies all three requirements and facilitates the formation of preorganized π-stacked dimers. The two fluorophores inside a G_2_–CB[8]_2_ complex form a discrete dimeric stack with a significant overlap of π electrons and a restricted interplanar spacing defined by the CB[8] cavities. Steric hindrance from both CB[8] macrocycles facilitates “mechanical” separation between all dimers in aqueous solution ensuring pairwise fluorophores perform as a discrete entity. More importantly, the dimers are stabilized by CB[8] clamping and remain as such for a sufficiently long period of time. Finally, discrete preorganized dimers can be readily obtained through our strategy in aqueous solution at ambient temperatures, and therefore do not require formation of a specific crystal^60,61^ or crystalline solvent at extremely low temperature.^62,63^ Moreover, owing our modular design, a variety of fluorophores are incorporated to give the corresponding π-stacked dimers without any limitation on fluorophore size in direct contrast to other methods.^64^

### 2.9 Controlling photophysics by clamping modules

#### 2.9.1 Suppression of radiative deactivation through non-parallel clamping

Complexation enhanced fluorescence is trivial for naphthyl-based guest molecules on account of their intrinsically high fluorescence efficiencies, whose quantum yield is almost unity even without complexation. An exception is **Np27Me** whose G_2_–CB[8]_2_ complex exhibits a quantum yield of 0.55, which is about 40% less than its G_1_–CB[7]_2_ complex or in a non-complexed solution, and much smaller than the G_2_–CB[8]_2_ complexes of other naphthyl homologues (Table 1). A reduction in quantum yield is accompanied by a dramatic decrease in the radiative rate constant, which, in turn, results in the longest fluorescence lifetime observed for any species in this study of up to 36.8 ns. A similar suppression in the radiative pathway is also observed for the G_2_–CB[8]_2_ complex of **Ph13Me** and the G_2_–CB[8]_3_ complex of **Ph135Me**, both of which exhibit a decreased quantum yield and an elongated lifetime. All three of these fluorescent molecules employ a non-parallel arrangement between their clamping modules, which suggests that non-parallel arrangements suppress the decay through radiative pathways. Clamping the dimer together in a non-parallel manner prevents any slippage of the two fluorophores along their extended axis and strongly restricts any intracomplex motions. As a consequence, the structural relaxation of the complex after photo-excitation towards a low-energy excited state is further retarded due to conformational rigidity amplified by non-parallel clamping. This restriction of motion is even more significant in triple-clamping cases, such as **Ph135Me** whose *k*_r_ and *ϕ*_F_ values are reduced compared to **Ph13Me**.

Radiative decay is practically prohibited in the case of **Ant14Me** when it is complexed with CB[8], either by dual clamping or by triple clamping, exhibiting negligible quantum yield in either case (Table 1). Fluorescence quenching in the G_2_–CB[8]_3_ complex of **Ant14Me** may be readily explained by triple clamping, however, it does not explain why complete quenching is also observed for its G_2_–CB[8]_2_ counterpart. One hypothesis is that the protruding anthracenyl moieties in one dimer may be long enough to interact with other protruding anthracenyl pairs located in another dimer, leading to some radiationless decay pathways that quickly deactivate the excited state. Interactions between protruding anthracenyl moieties is also supported by the diminished quantum yield observed for its G_1_–CB[7]_2_ complex compared to that of other anthracenyl homologues. Another possibility leading to radiationless decay may be a transition from a singlet to a triplet state through intersystem crossing, however, this requires substantial further study of the dynamics of **Ant14Me** complexes in their excited states.

#### 2.9.2 H–H and H–T stacking of the fluorophore dimer

When the G_2_–CB[8]_2_ complex contains non-parallel clamping modules (*e.g.***Np27Me**, **Ph13Me**, and **Ph135Me**), the way in which the two fluorophores are stacked with respect to one another is fixed. However, co-facial stacking of the two fluorophores may adopt either a head-to-head (H–H) or head-to-tail (H–T) configuration when the clamping modules are parallel. This is not an issue for symmetric fluorophores such as **Ant910Me** and **Ph14Me**, as the H–H and H–T orientations are indistinguishable.

Interestingly, the G_2_–CB[8]_2_ complex of **Ant15Me** also adopts a single H–H stacking configuration, as the spacing between its two off-line clamping modules is too large to allow for a feasible H–T configuration (Scheme S3†). This specific stacking configuration is also revealed in the ^1^H NMR spectrum of its G_2_–CB[8]_2_ complex, in which the protons of the 1,5-anthracenyl moieties exhibit sharp and well-resolved signals (Fig. S9†). In contrast, **Np15Me** with a smaller gap between its off-line clamping modules may allow for both H–H and H–T stacking configurations of the two fluorophores, which leads to a significant broadening of proton signals in the NMR of its G_2_–CB[8]_2_ complex. Moreover, all the proton signals are equally broadened suggesting a dynamic process that involves the entire complex, which very likely correlates to an interconversion between H–H and H–T stacking configurations with an exchanging rate on the intermediate NMR timescale (Fig. S7†). As a result, the aromatic fluorophores in the G_2_–CB[8]_2_ complexes **Ant15Me** and **Np15Me** both exhibit a substantial overlap of π-electrons in a less *J* aggregate-like fashion, leading to smaller bathochromic shifts in their emission maxima (Fig. 5) compared to other fluorescent molecules.^65^ Considering their red-shift in absorption, the smaller bathochromic shifts in emission maxima may also correlate to an anti-Kasha behavior as mentioned above, which requires further investigation.

Although both H–H and H–T stacking should be feasible by **Np14Me** and **Ant14Me**, the NMR spectra of their G_2_–CB[8]_2_ complexes suggest a preference towards head-to-head stacking. The protons residing on the protruding ring exhibit an upfield shift due to shielding of the aromatic ring current, which is best explained by a head-to-head overlapping of the fluorophores. This further suggests that the π–π interactions play a role in determining energy-favorable stacking configurations.

#### 2.9.3 Substituents on the clamping modules

In addition to methyl (Me) groups in the *para*-position of the aryl clamping modules, other substituents including amino- (NH_2_), methoxy- (OMe), dimethylamino- (NMe_2_), isopropyl- (CMe_2_), and methylthio- (SMe) readily form monomeric and dimeric complexes with CB[7] and CB[8], respectively, in the same manner as the parent methyl compounds. As the aryl clamping modules are both bound inside the CB cavity for the G_1_–CB[7]_2_ and G_2_–CB[8]_2_ complexes, they exhibit similar diffusion coefficients regardless of the variation in *para*-substituents (Fig. 3b).

**Fig. 9:**
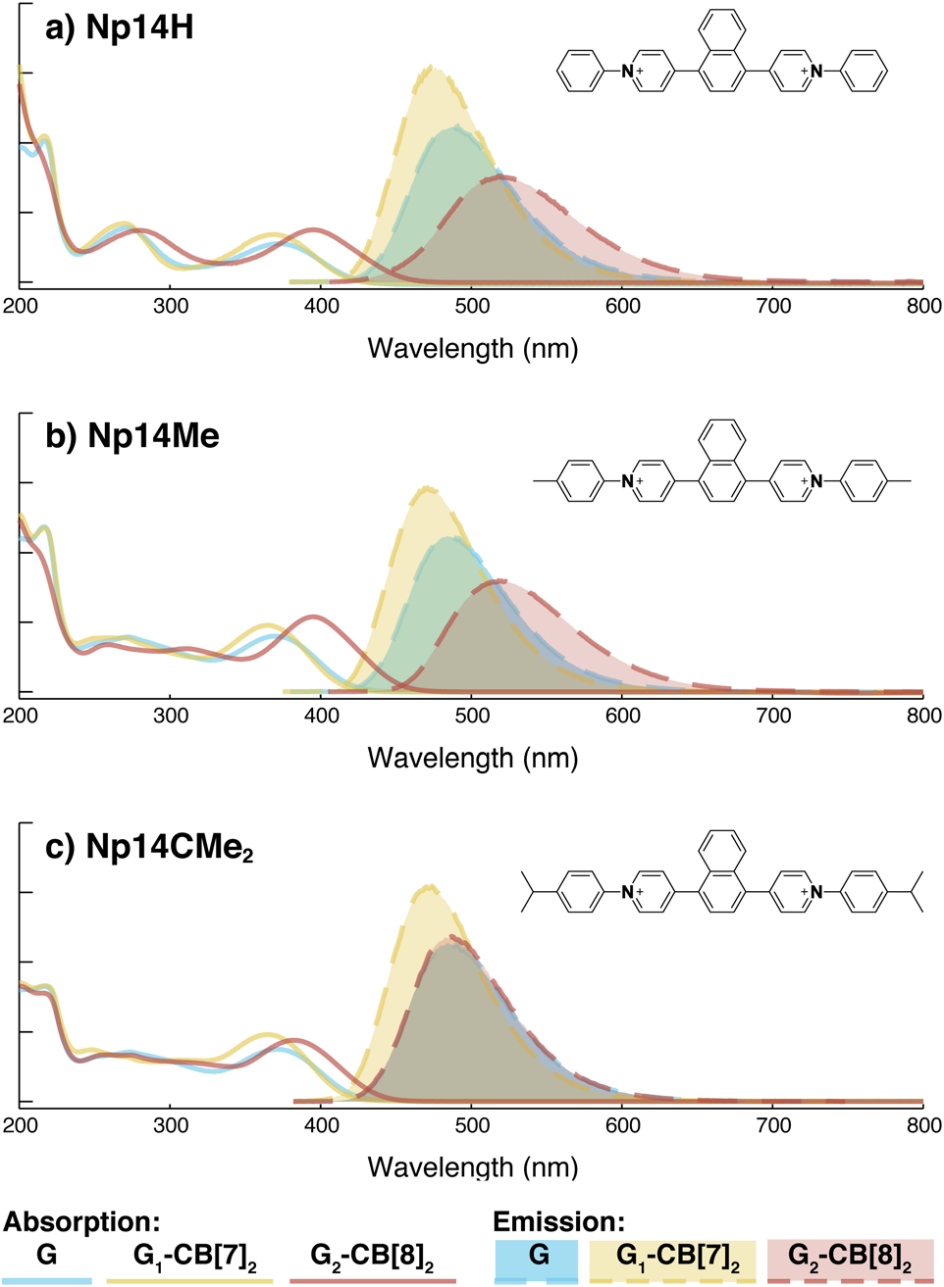
Absorption (solid line) and emission (dash line with filling color) spectra of 1,4-naphthyl based fluorescent molecules (blue) with variation in substituents including (a) **Np14H**, (b) **Np14Me**, (c) **Np14CMe2**, along with their G_1_–CB[7]_2_ complexes (yellow) and G_2_–CB[8]_2_ complexes (red). Aqueous solution of each species are tested under a uniform guest concentration at 298 K. The intensity is not normalized but scaled up by the same factor. The Cl^−^ counterions are omitted for clarity.

On the other hand, the photophysical properties of the dimeric stacked fluorophores are indeed affected by the size of the aryl substituents. As shown in Fig. 9, **Np14H**, a naphthyl fluorescent molecule without any substituent on its clamping module displays the same absorption and emission spectra as those of **Np14Me**. However, a significant difference of the emission maximum is observed for the G_2_–CB[8]_2_ complex of **Np14CMe2**. As both the absorption and emission spectra of G and G_1_–CB[7]_2_ of **Np14CMe2** are similar to those of **Np14H** and **Np14Me**, this difference observed for the G_2_–CB[8]_2_ complex must stem from a certain variation in the stacking of the naphthyl pair, which is very likely caused by a significant volume exclusion between neighboring isopropyl substituents. The resultant stacking configuration in G_2_–CB[8]_2_ of **Np14CMe2** still leads to a red-shifted absorption band corresponding to π-electron delocalization in the preorganized dimer. It seems that the preorganized dimer (in this case) does not result in an effective formation of an excimer-like state, as the emission maximum is very similar to that in pristine solution without an obvious bathochromic shift. This observation thus offers an additional opportunity to tune the photophysical properties of stacked fluorophores by choosing appropriate substituents.

## 3 Conclusions

In summary, we have demonstrated a modular strategy to design a new class of fluorescent molecules that (i) generate discrete, dimeric stacked fluorophores in aqueous solution and (ii) are constrained by CB[8]-mediated multiple clamping. This modular design is surprisingly applicable and flexible and has been validated by testing nine different fluorophore cores ranging in size, shape, and geometric variation of their clamping modules. When complexed with CB[7], all fluorescent molecules are dispersed in aqueous solution as discrete monomers, exhibiting an impressively high fluorescence efficiency. On the other hand, complexation with CB[8] as 2 : 2 or 2 : 3 complexes leads to the immediate formation of discrete dimeric stacked fluorophores. Multiple CB[8] clamping results in stable, preorganized ground-state dimers, which can be readily photoexcited to excimer-like states, displaying significant bathochromic shifts in absorption and emission with elongated fluorescence lifetimes. Bathochromic shifts in the emission spectra can be readily tuned by controlling the stacking of fluorophores through specific variations in the clamping modules (through off-line alignment or altering substituents).

We demonstrate that intracomplex motion in the preorganized dimers is significantly restricted, which suppresses both radiative and non-radiative deactivation, resulting in a substantially high quantum yield (up to 1) despite formation of excimer-like states. Some complexes are further restricted through non-parallel or triple clamping, which slows down radiative relaxation to an even greater extent, leading to elongated excited-state lifetimes up to 37 ns in aqueous solution. Moreover, complexes stabilized by multiple non-parallel clamping exhibit self-sorting in the presence of excess CB[8], which facilitates the design and fabrication of hierarchical functional structures.

While only arylpyridinium moieties have been employed as the clamping module in this study, current investigations suggest other chemical motifs with rigid structure exhibit the same clamping feature. The high rigidity ensures intrinsically low conformational entropy change during complexation, thus facilitating the formation of a long-lived, multicomponent complex in aqueous solution.

From a fundamental point of view, this study offers a model system with explicitly stable dimeric structures and tuneable features that can be utilized as a platform to study various intermolecular processes including excimer formation, charge transfer, exciton coupling, and singlet fission. Moreover, such a modular molecular design towards quadrupolar fluorescent molecules may provide a feasible toolbox in pursuit of distinct features such as large two-photon cross-sections^66,67^ and non-Kasha behavior.^68^ On the practical side, CB[7]- and CB[8]-mediated fluorescent complexes developed here are promising candidates for various (biological) imaging applications on account of their emergent photophysical properties such as long lifetimes, high emission brightness, and red-shifted excitation bands.

## Conflicts of interest

There are no conflicts to declare.

## Supplementary Material

SC-011-C9SC04587B-s001

SC-011-C9SC04587B-s002
