# Supplementary material for: Controlling the structure and photophysics of fluorophore dimers using multiple cucurbit[8]uril clampings
Source: Chem Sci. 2019 Dec 6;11(3):812–25. doi: 10.1039/c9sc04587b (PMC8146025; doi:10.1039/c9sc04587b)
Supplement: SC-011-C9SC04587B-s001 [file SC-011-C9SC04587B-s001.pdf]

Electronic Supplementary Material (ESI) for Chemical Science.
This journal is © The Royal Society of Chemistry 2019

Supplemental Information

Controlling the Structure and Photophysics of Fluorophore Dimers using Multiple Cucurbit[8]uril Clampings

Guanglu Wu,[†] Youn Jue Bae,[‡] Magdalena Olesińska,[†] Daniel Antón-García,[¶] István Szabó,[§] Edina Rosta,[§] Michael R. Wasielewski,[‡] and Oren A. Scherman[*,†]

[†] Melville Laboratory for Polymer Synthesis, Department of Chemistry, University of Cambridge, Lensfield Road, Cambridge CB2 1EW, UK.

[‡] Department of Chemistry and Institute for Sustainability and Energy at Northwestern, Northwestern University, Evanston, Illinois 60208-3113, United States

¶Department of Chemistry, University of Cambridge, Lensfield Road, Cambridge, CB2 1EW, UK.

§Department of Chemistry, King's College London, 7 Trinity Street, London, SE1 1DB, UK.

[*] E-mail: oas23@cam.ac.uk

Table of Contents

SI-1 Materials and methods 1
SI-2 Synthesis and characterization 3
SI-3 Diffusion coefficients determined by DOSY 10
SI-4 Complexation of Ant910Me with CB[7] and CB[8] 11
SI-5 Complexation of Np27Me with CB[7] and CB[8] 14
SI-6 Complexation of Np14Me with CB[7] and CB[8] 15
SI-7 Complexation of Np15Me with CB[7] and CB[8] 15
SI-8 Complexation of Ant15Me with CB[7] and CB[8] 16
SI-9 Complexation of Ant14Me with CB[7] and CB[8] 17
SI-10 Complexation of Ph14Me with CB[7] and CB[8] 19
SI-11 Complexation of Ph13Me with CB[7] and CB[8] 20
SI-12 Complexation of Ph135Me with CB[7] and CB[8] 20
SI-13 VT-NMR study of the Ant910Me$_2$-CB[8]$_2$ complex 22
SI-14 Transient absorption of G$_1$-CB[7]$_2$ and G$_2$-CB[8]$_2$ complex of Ant910Me 24
SI-15 MD simulation of Ant910Me$_2$-CB[8]$_2$ 29
SI-16 Head-to-head and head-to-tail stacking 30
Reference 31

SI-1 Materials and methods

Materials. All the materials as following were purchased from commercial suppliers and used without further purification: 9,10-dibromoanthracene (98%, Sigma-Aldrich), 1,5-dibromoanthracene (98%, TCI), 1,4-diaminoanthraquinone (90%, Sigma-Aldrich), tert-butyl nitrite (90%, Sigma-Aldrich), copper(II) bromide (99%, Sigma-Aldrich), tin(II) chloride dihydrate (extra pure, Fisher Scientific), 1,5-dibromonaphthalene (98%, TCI), 1,4-dibromonaphthalene (98%, Acros Organics), 2,7-dibromonaphthalene (99%, Sigma-Aldrich), 1,4-dibromobenzene (98%, Alfa Aesar), 1,3-dibromobenzene (97%, Sigma-Aldrich), 1,3,5-tribromobenzene (98%, Sigma-Aldrich), 4-pyridinylboronic acid (90%, Sigma-Aldrich), tetrakis(triphenylphosphine) palladium(0) (99%, Alfa Aesar), 1-chloro-2,4-dinitrobenzene (98%, Alfa Aesar), *p*-toluidine (99%, Sigma-Aldrich), aniline (99.5%, Sigma-Aldrich), 4-isopropylaniline (99%, Sigma-Aldrich), *p*-anisidine (99%, Sigma-Aldrich), 4-(methylthio)aniline (98%, Alfa Aesar), *p*-phenylenediamine (97 %, Alfa Aesar), N,N-dimethyl-*p*-phenylenediamine (97%, Sigma-Aldrich), 1-adamantanamine hydrochloride (97%, Sigma-Aldrich), 2,2-Dimethyl-2-silapentane-5-sulfonate sodium salt (97%, Sigma-Aldrich). All solvents and reagents were purchased from Sigma-Aldrich (UK) or Fisher Scientific and used as received. Cucurbit[8]uril (CB[8]) and CB[7] were synthesized and purified according to a published procedure [1]. Milli-Q water (18.2 MΩ·cm) was used for preparation of all non-deuterated aqueous solutions.

High Pressure Liquid Chromatography (HPLC). HPLC was employed to purify and collect dicationic species by using a Phenomenex C18 Kinetic-Evo column with a 5 micron pore size, a 110 Å particle size and with the dimensions 150 x 21.2 mm. A gradient from 5% acetonitrile 95% water to 100% acetonitrile was run with 0.1% TFA.

Nuclear Magnetic Resonance Spectroscopy (NMR). ^{1}H NMR, ^{13}C NMR, COSY, DOSY, and NOESY spectra were acquired in heavy water (D_2O) or DMSO-D6 at 298 K and recorded on a Bruker AVANCE 500 with TCI Cryoprobe system (500 MHz) being controlled by TopSpin2. NOESY experiments were carried out using a standard pulse sequence 'noesygpphpp' with 2 s relaxation delay and 1000 ms mixing time. The concentration of CB[8] or CB[7] deuterated aqueous solution was calibrated by the internal standard, DSS sodium salt.

Variable-Temperature ^{1}H NMR (VT-NMR). ^{1}H NMR spectra with variable temperature were recorded by a Bruker AVANCE 500 with TCI Cryoprobe system (500 MHz) as well as a Bruker DPX-200 (200 MHz) spectrometers. The temperature of 500 MHz and 200 MHz spectrometers is calibrated by MeOD (D, 99.8 %) and ethylene glycol (80 % in DMSO-D_6) standards, respectively. VT-NMR in a low-field spectrometer is equivalent to heating up the samples so that it enables the study of very slow dynamics. [2]

UV/Vis Spectroscopy and Fluorescence Spectroscopy. UV/Vis and fluorescence spectra were recorded on a Varian Cary 400 UV/Vis spectrophotometer and Varian Cary Eclipse fluorescence spectrophotometer, respectively, using a Hellma 114F-QS cuvette with 10x4 mm path length at 298 K.

Time-Correlated Single Photon Counting (TCSPC). TCSPC was recorded at FS5 Spectrofluorometer, Edinburgh Instruments with a laser of 360 nm for the fluorescence lifetime measurements. A Hellma 114F-

QS cuvette with 10x4 mm path length was used for all measurements.

Fluorescence Quantum Yield Measurements. Absolute fluorescence quantum yield was also recorded at FS5 Spectrofluorometer, Edinburgh Instruments with an integrating sphere module (SC-30). A Hellma 114F-QS cuvette with 10x4 mm path length was used for all measurements at 298 K. To avoid the scattering of incident light at liquid-air interface, testing solutions with a volume larger than 1.2 mL were used to obtain reliable quantum yield results. The concentration of the testing solution was adjusted in order to yield an optical density in the range of 0.1-0.06.

Femtosecond and nanosecond transient absorption (fsTA and nsTA) spectroscopy. Femtosecond and nanosecond transient absorption (TA) spectroscopy experiments were conducted using a regeneratively-amplified Ti:Sapphire laser system operating at 828 nm and a 1 kHz repetition rate as previously described.[3] Solution samples were prepared in a 2 mm path length glass cuvette. All the samples are in D_2O excited at 414 nm (1μJ/pulse) at 298K ambient condition. The degassed samples were treated with three freeze-pump-thaw cycles.

Isothermal Titration Calorimetry (ITC). All ITC experiments were carried out on a Microcal ITC200 at 298.15 K in pure water or 10 mM sodium phosphate buffer (pH = 7.0). In a typical ITC, the host molecule (CB[8]) was in the sample cell, and the guest molecule was in the injection syringe with a concentration of about ten times the concentration of the host. The concentration of CB[8] was calibrated by the titration with a standard solution of 1-adamantanamine. In order to avoid bias or potentially arbitrary offsets caused by manual adjustment of baseline, all raw data (thermograms) of ITC were integrated by NITPIC (v.1.2.2), fitted in Sedphat (v.15.2b), and visualized through GUSSI (v.1.2.1).[4]

SI-2 Synthesis and characterization

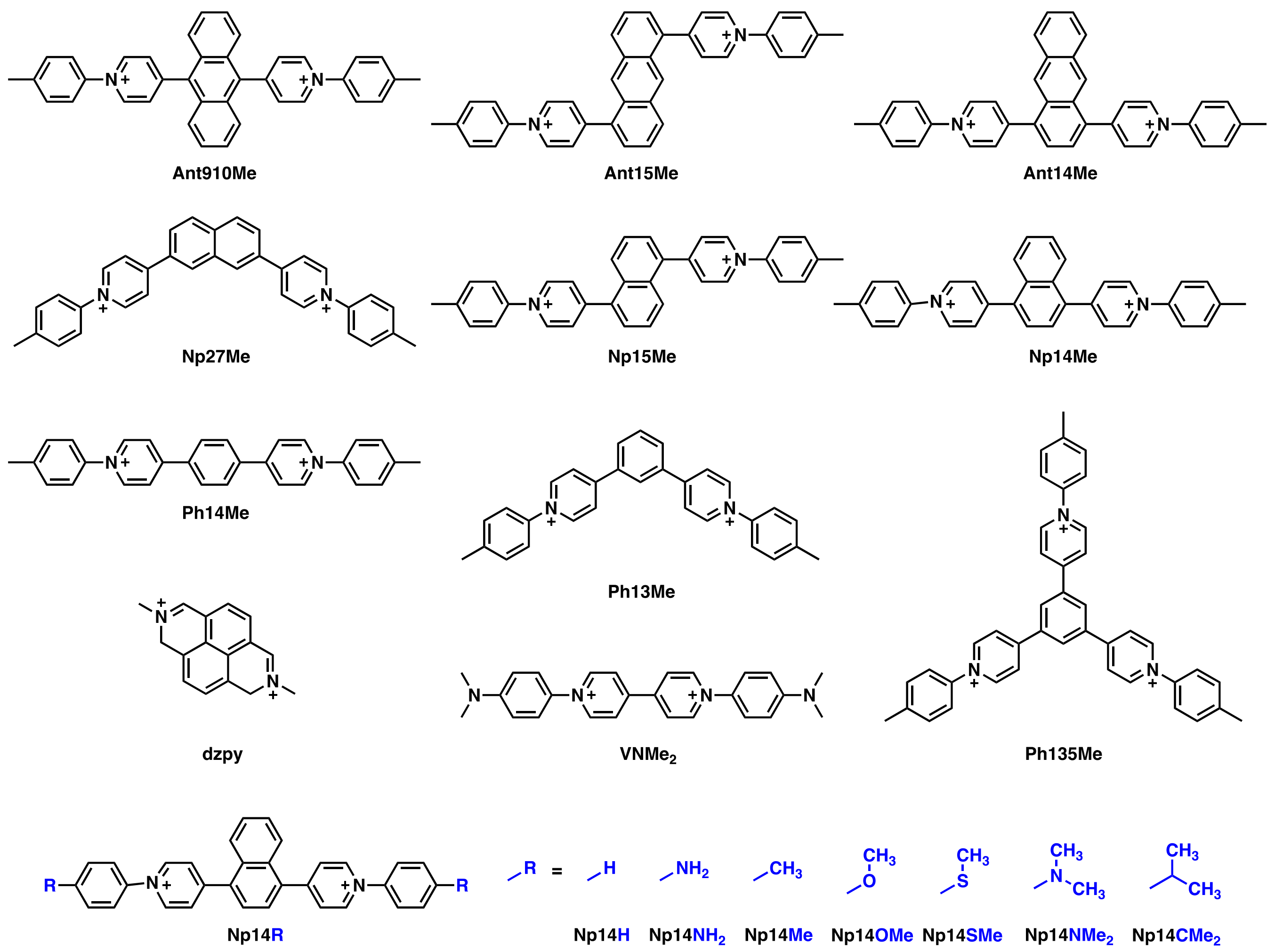

Scheme S1. Molecules related to this work (Counter anion is Cl^-).

As exemplified by **Ant14Me** in Scheme S2, a general synthesis of extended diarylviologen derivatives in this work starts with Suzuki-Miyaura cross-coupling[5] of two pyridin-4-yl groups onto the fluorophore core, followed by the transformation of the pryidin-4-yl groups into arylpyridinium salts through a Zincke reaction.[6]

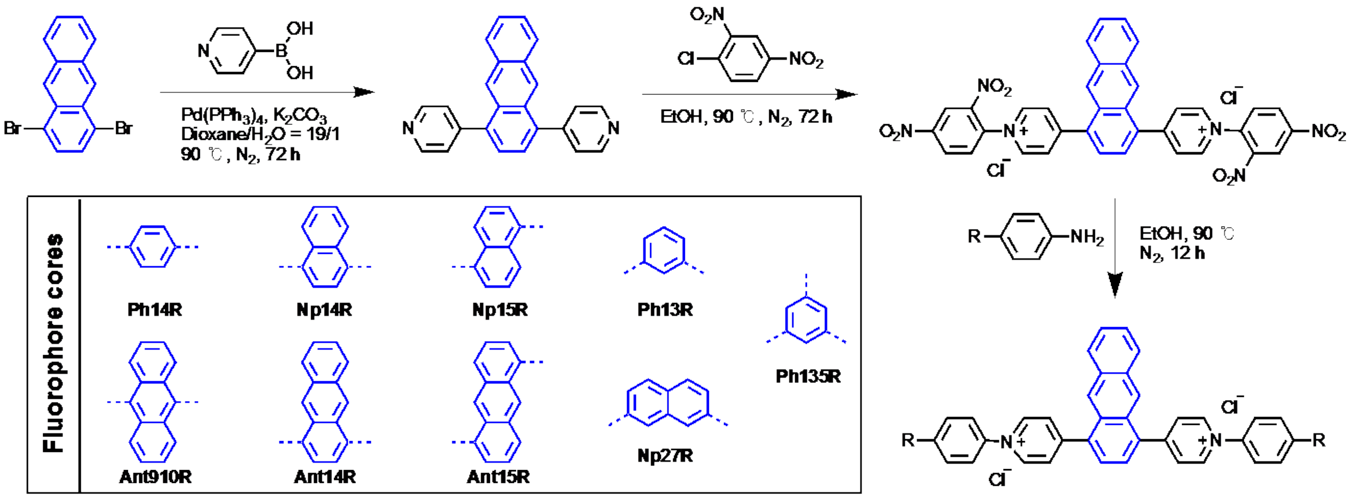

Scheme S2. Synthetic route of **Ant14Me** (when R = methyl).

All of the dibromo- and tribromo- starting materials are directly purchased from suppliers, except 1,4-dibromoanthracene which was synthesized according previously reported procedures:[7,8]

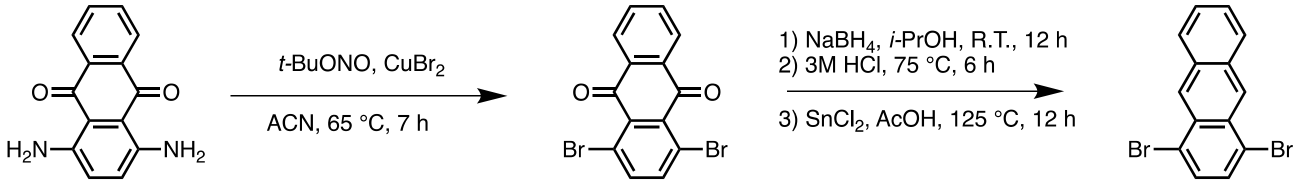

***1,4-dibromoanthracene-9,10-dione*.**[7] 1.5 g (Yield: 54%) ^{1}H NMR (500 MHz, $CDCl_3$) δ (ppm): 8.20 (ddd, J = 5.8, 3.3, 0.7 Hz, 2H), 7.81 (d, J = 0.6 Hz, 2H), 7.79 (dd, J = 5.8, 3.3 Hz, 2H). ^{13}C NMR (126 MHz, $CDCl_3$) δ (ppm): 181.77, 140.78, 134.38, 133.71, 133.62, 127.10, 122.21.

***1,4-dibromoanthracene*.**[8] 0.46 g (Yield: 56 %) ^{1}H NMR (500 MHz, $CDCl_3$) δ (ppm): 8.83 (s, 2H), 8.11 (dd, J = 6.4, 3.3 Hz, 2H), 7.62 (s, 2H), 7.58 (dd, J = 6.5, 3.2 Hz, 2H). ^{13}C NMR (126 MHz, $CDCl_3$) δ (ppm): 132.61, 130.43, 128.98, 128.35, 127.32, 126.92, 122.77.

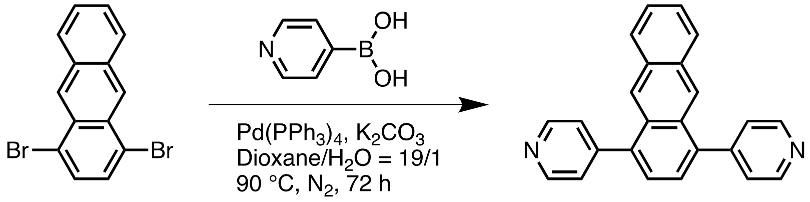

1,4-di(pyridin-4-yl)anthracene. $Pd(PPh_3)_4$ (170 mg, 0.15 mmol, 5 mol% of Br) was added into a mixture of 4-pyridinylboronic acid (398 mg, 2.91 mmol, 2.2 equiv.), 1,4-dibromoanthracene (450 mg, 1.34 mmol, 1 equiv.), and K_2CO_3 (1.3 g, 9.42 mmol, 7 equiv.) in the N_2-protected glove box. After three freeze-pump-thaw cycles, 16 mL of degassed dioxane/H_2O (19/1, v/v) were added into above mixture and stirred under nitrogen atmosphere for 72 h at 90 °C. After cooling to room temperature, the mixture was extracted with $CHCl_3$, washed with H_2O for three times, and dried over Na_2SO_4. The crude was concentrated and purified *via* a short silica gel column, firstly using ethyl acetate (EA) as eluent to remove fast-moving species followed by adding aqueous ammonia (35%) into the EA eluent (0.5% v/v) to collect the fluorescent spot. A yellow solid of 365 mg was obtained in 82% yield. ^{1}H NMR (500 MHz, $CDCl_3$) δ (ppm): 8.85 (d, *J* = 5.1 Hz, 4H), 8.83 (d, *J* = 5.4 Hz, 2H), 8.47 (s, 2H), 8.02 – 7.91 (m, 2H), 7.58 (d, *J* = 5.5 Hz, 4H), 7.53 – 7.49 (m, 2H).

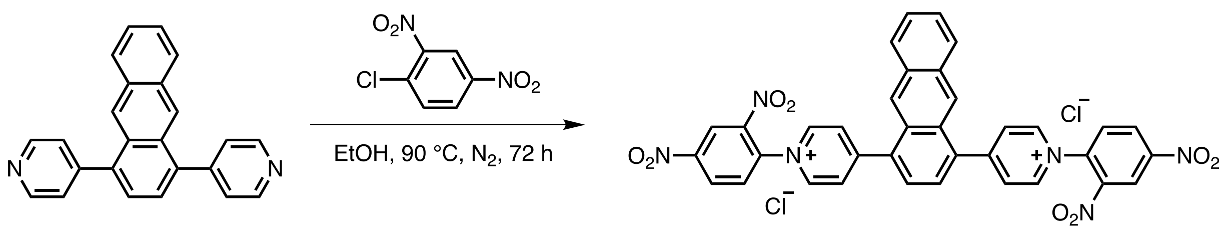

4,4'-(anthracene-1,4-diyl)bis(1-(2,4-dinitrophenyl)pyridin-1-ium) chloride (Ant14DNB). 1-Chloro-2,4-dinitrobenzene (1.48 g, 7.3 mmol, 12 equiv.) and 1,4-di(pyridin-4-yl)anthracene (200 mg, 0.60 mmol, 1 equiv.) were refluxed in ethanol (40 mL) at 90 °C for 72 h under nitrogen atmosphere (short reaction time will result in a large amount of mono-substituted product). After evaperating solvent under reduced pressure, the solid was re-dissolved in MeOH (3 mL) and was added dropwise into diethyl ether (120 mL) to precipitate products. Decantation of the supernatant after centrifuging by 8000 rpm for 10 mins at 4 °C afforded crude products (containing small amount of mono-substituted by-product) for further purification by HPLC. After drying by vacuum oven, the crude products were dissolved in ACN/H_2O mixture (30% v/v) and purified by a preparative reverse-phase HPLC using an eluent gradient of ACN/H_2O. The collected fraction was lyophilized to give 340 mg red solid in 77% yield. ^{1}H NMR (500 MHz, Methanol-*d*$_4$) δ (ppm): 9.50 (d, *J* = 7.0 Hz, 4H), 9.38 (d, *J* = 2.5 Hz, 2H), 9.03 (dd, *J* = 8.6, 2.5 Hz, 2H), 8.78 (d, *J* = 7.1 Hz, 4H), 8.77 (s, 2H), 8.46 (d, *J* = 8.6 Hz, 2H), 8.14 (dd, *J* = 6.4, 3.2 Hz, 2H), 8.05 (s, 2H), 7.69 (dd, *J* = 6.6, 3.1 Hz, 2H). ^{13}C NMR (125 MHz, Methanol-*d*$_4$) δ (ppm): 161.53, 151.36, 147.37, 144.83, 140.14, 138.55, 134.21, 132.63, 131.26, 130.32, 129.43, 129.08, 128.95, 125.90, 123.36. LCMS: *m/z* $[M-2Cl]^{2+}$ calcd. for $C_{36}H_{22}N_6O_8^{2+}$: 333.1, found: 333.4.

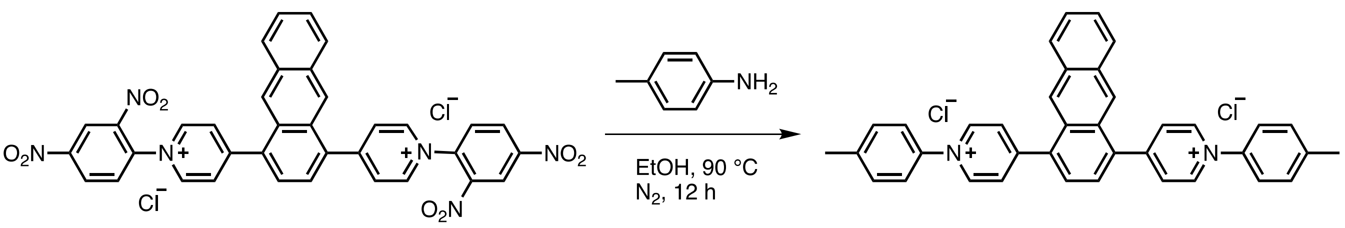

4,4'-(anthracene-1,4-diyl)bis(1-(p-tolyl)pyridin-1-ium) chloride (Ant14Me). *p*-Toluidine (30 mg, 0.28

mmol, 14 equiv.) and 4,4'-(anthracene-1,4-diyl)bis(1-(2,4-dinitrophenyl)pyridin-1-ium) chloride (15 mg, 0.02 mmol, 1 equiv.) were refluxed in ethanol (20 mL) at 90 °C for 12 h under nitrogen atmosphere. After removing solvent under reduced pressure, the solid was re-dissolved in MeOH (1 mL) and was added dropwise into diethyl ether (40 mL) to precipitate products. Decant the supernatant after centrifuging by 8000 rpm for 10 mins at 4 °and repeat above MeOH-Ether step for another two times in order to wash off the excess of *p*-toluidine. Drying in vacuum oven gave 10 mg of orange solid in 83 % yield. ^{1}H NMR (500 MHz, D_2O) δ (ppm): 9.27 (d, *J* = 6.7 Hz, 4H), 8.66 (s, 2H), 8.55 (d, *J* = 6.6 Hz, 4H), 8.10 (dd, *J* = 6.5, 3.2 Hz, 2H), 7.90 (s, 2H), 7.77 (d, *J* = 8.5 Hz, 4H), 7.68 (dd, *J* = 6.6, 3.1 Hz, 2H), 7.64 (d, *J* = 8.2 Hz, 4H), 2.54 (s, 6H). ^{13}C NMR (126 MHz, D_2O) δ (ppm): 157.55, 144.07, 142.68, 140.08, 136.63, 132.04, 130.99, 129.03, 128.12, 127.96, 127.43 (two carbon overlap in this peak), 124.52, 123.71, 20.31. ESI-MS: *m/z* $[M-2Cl]^{2+}$ calcd. for $C_{38}H_{30}N_2^{2+}$: 257.120, found: 257.121.

Products with different fluorophore cores were synthesized *via* the same procedures as **Ant14Me**. **Np14Me**, **Np14R**, and **Ph14Me** were obtained from our previous work.[9]

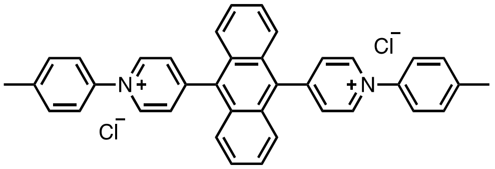

9,10-di(pyridin-4-yl)anthracene. 315 mg (Yield: 54 %) ^{1}H NMR (500 MHz, $CDCl_3$) δ (ppm): 8.89 (d, *J* = 5.7 Hz, 4H), 7.62 (dd, *J* = 6.8, 3.2 Hz, 4H), 7.45 (d, *J* = 5.7 Hz, 4H), 7.40 (dd, *J* = 6.9, 3.2 Hz, 4H).

4,4'-(anthracene-9,10-diyl)bis(1-(2,4-dinitrophenyl)pyridin-1-ium) chloride (Ant910DNB). 126 mg (Yield: 31 %) ^{1}H NMR (500 MHz, DMSO-d_6) δ (ppm): 9.63 (d, *J* = 6.3 Hz, 4H), 9.20 (d, *J* = 2.6 Hz, 2H), 9.09 (dd, *J* = 8.6, 2.6 Hz, 2H), 8.94 – 8.88 (m, 4H), 8.69 (d, *J* = 6.3 Hz, 4H), 8.59 (d, *J* = 8.6 Hz, 2H), 7.59 (m, 4H). LCMS: *m/z* $[M-2Cl]^{2+}$ calcd. for $C_{36}H_{22}N_6O_8^{2+}$: 333.1, found: 333.4.

4,4'-(anthracene-9,10-diyl)bis(1-(p-tolyl)pyridin-1-ium) chloride (Ant910Me). 15 mg (Yield: 90%) ^{1}H NMR (500 MHz, D_2O) δ (ppm): δ 9.39 (d, *J* = 7.0 Hz, 4H), 8.46 (d, *J* = 6.9 Hz, 4H), 7.83 (d, *J* = 8.6 Hz, 4H), 7.79 – 7.73 (m, 4H), 7.70 – 7.61 (m, 8H), 2.55 (s, 6H). ^{13}C NMR (126 MHz, D_2O) δ (ppm): 157.41, 144.38, 142.80, 140.21, 132.63, 131.06, 131.03, 128.15, 127.46, 125.34, 123.81, 20.32. ESI-MS: *m/z* $[M-2Cl]^{2+}$ calcd. for $C_{38}H_{30}N_2^{2+}$: 257.120, found: 257.121.

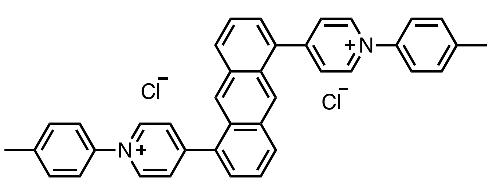

1,5-di(pyridin-4-yl)anthracene. 150 mg (Yield: 39 %) ^{1}H NMR (500 MHz, $CDCl_3$) δ (ppm): 8.82 (d, *J* = 5.9 Hz, 4H), 8.47 (s, 2H), 7.98 (d, *J* = 8.7 Hz, 2H), 7.59 – 7.50 (m, 6H), 7.44 (d, *J* = 6.6 Hz, 2H).

4,4'-(anthracene-1,5-diyl)bis(1-(2,4-dinitrophenyl)pyridin-1-ium) chloride (Ant15DNB). 160 mg (Yield: 80 %) ^{1}H NMR (500 MHz, MeOD) δ (ppm): 9.44 (d, *J* = 6.8 Hz, 4H), 9.37 (d, *J* = 2.5 Hz, 2H), 9.01 (dd, *J* = 8.6, 2.5 Hz, 2H), 8.89 (s, 2H), 8.74 (d, *J* = 6.8 Hz, 4H), 8.44 (d, *J* = 8.6 Hz, 2H), 8.39 (dd, *J* = 8.6, 1.1 Hz, 2H), 8.00 (dd, *J* = 6.9, 1.1 Hz, 2H), 7.83 (dd, *J* = 8.6, 6.9 Hz, 2H). ^{13}C NMR (126 MHz, MeOD) δ (ppm): 161.87, 151.31, 147.11, 144.85, 140.16, 134.94, 134.19, 134.09, 132.66, 131.80, 131.22, 130.10, 129.57, 127.15, 125.85, 123.34. LCMS: *m/z* $[M-2Cl]^{2+}$ calcd. for $C_{36}H_{22}N_6O_8^{2+}$: 333.1, found: 333.4.

4,4'-(anthracene-1,5-diyl)bis(1-(p-tolyl)pyridin-1-ium) chloride (Ant15Me). 20 mg (Yield: 95%) ^{1}H NMR (500 MHz, D_2O) δ (ppm): δ 9.24 (d, *J* = 6.5 Hz, 4H), 8.76 (s, 2H), 8.54 (d, *J* = 6.5 Hz, 4H), 8.30 (d, *J* = 8.6 Hz, 2H), 7.87 (d, *J* = 6.8 Hz, 2H), 7.81 – 7.74 (m, 6H), 7.64 (d, *J* = 8.2 Hz, 4H), 2.54 (s, 6H). ^{13}C NMR (126 MHz, D_2O) δ (ppm): 157.91, 143.92, 142.59, 140.08, 133.51, 132.19, 131.96, 130.97, 129.69, 128.87, 127.94, 125.73, 124.44, 123.70, 20.30. ESI-MS: *m/z* $[M-2Cl]^{2+}$ calcd. for $C_{38}H_{30}N_2^{2+}$: 257.120, found: 257.121.

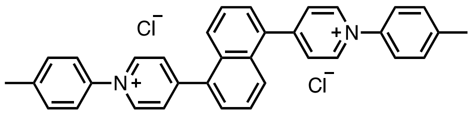

1,5-di(pyridin-4-yl)naphthalene. 360 mg (Yield: 80 %) ^{1}H NMR (500 MHz, $CDCl_3$) δ (ppm): 8.76 (d, *J* = 5.1 Hz, 4H), 7.90 (d, *J* = 8.5 Hz, 2H), 7.54 (t, *J* = 7.7 Hz, 2H), 7.46 (m, 6H).

4,4'-(naphthalene-1,5-diyl)bis(1-(2,4-dinitrophenyl)pyridin-1-ium) chloride (Np15DNB). 435 mg (Yield: 95 %) ^{1}H NMR (500 MHz, MeOD) δ (ppm): 9.45 (d, *J* = 6.6 Hz, 4H), 9.36 (d, *J* = 2.5 Hz, 2H), 9.00 (dd, *J* = 8.6, 2.5 Hz, 2H), 8.66 (d, *J* = 6.8 Hz, 4H), 8.45 (d, *J* = 8.6 Hz, 2H), 8.32 (d, *J* = 8.5 Hz, 2H), 8.03 (d, *J* = 7.4 Hz, 2H), 7.95 (dd, *J* = 8.5, 7.1 Hz, 2H). LCMS: *m/z* $[M-2Cl]^{2+}$ calcd. for $C_{32}H_{20}N_6O_8^{2+}$: 308.1, found: 308.4.

4,4'-(naphthalene-1,5-diyl)bis(1-(p-tolyl)pyridin-1-ium) chloride (Np15Me). 35 mg (Yield: 96%) ^{1}H NMR (500 MHz, D_2O) δ (ppm): δ 9.22 (d, *J* = 6.2 Hz, 4H), 8.46 (d, *J* = 6.2 Hz, 4H), 8.20 (d, *J* = 8.4 Hz, 2H), 7.92 – 7.81 (m, 4H), 7.74 (d, *J* = 8.2 Hz, 4H), 7.62 (d, *J* = 8.1 Hz, 4H), 2.53 (s, 6H). ^{13}C NMR (126 MHz, D_2O) δ (ppm): 157.94, 143.87, 142.58, 140.05, 134.47, 130.92, 130.02, 129.25, 129.06, 127.29, 127.16, 123.68, 20.27. ESI-MS: *m/z* $[M-2Cl]^{2+}$ calcd. for $C_{34}H_{28}N_2^{2+}$: 232.112, found: 232.113.

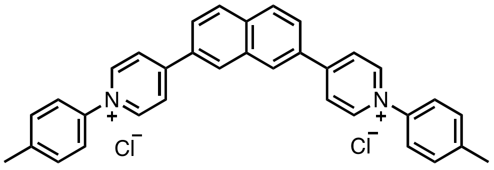

2,7-di(pyridin-4-yl)naphthalene. 280 mg (Yield: 66 %) ^{1}H NMR (500 MHz, $CDCl_3$) δ (ppm): 8.76 – 8.70 (m, 4H), 8.21 (dd, *J* = 1.6, 0.8 Hz, 2H), 8.02 (d, *J* = 8.7 Hz, 1H), 7.82 (dd, *J* = 8.6, 1.8 Hz, 2H), 7.68 – 7.63 (m, 4H). ^{13}C NMR (126 MHz, $CDCl_3$) δ (ppm): 150.59, 148.09, 136.53, 133.73, 133.51, 128.97,

127.06, 125.72, 121.99.

4,4'-(naphthalene-2,7-diyl)bis(1-(2,4-dinitrophenyl)pyridin-1-ium) chloride (Np27DNB). 460 mg (Yield: 92 %) ^{1}H NMR (500 MHz, MeOD) δ (ppm): 9.36 (d, *J* = 7.2 Hz, 4H), 9.33 (d, *J* = 2.5 Hz, 2H), 9.09 – 9.06 (m, 2H), 8.97 (dd, *J* = 8.6, 2.5 Hz, 2H), 8.92 (d, *J* = 7.1 Hz, 4H), 8.43 (dd, *J* = 8.7, 1.9 Hz, 2H), 8.38 (dd, *J* = 8.6, 4.5 Hz, 4H). ^{13}C NMR (126 MHz, MeOD) δ (ppm): 160.05, 151.21, 147.10, 144.82, 140.03, 138.30, 134.68, 133.73, 132.78, 132.67, 131.22, 131.14, 128.29, 126.26, 123.26. LCMS: *m/z* [M-2Cl]$^{2+}$ calcd. for $C_{32}H_{20}N_6O_8^{2+}$: 308.1, found: 308.4.

4,4'-(naphthalene-2,7-diyl)bis(1-(p-tolyl)pyridin-1-ium) chloride (Np27Me). 20 mg (Yield: 95%) ^{1}H NMR (500 MHz, D_2O) δ (ppm): δ 9.12 (d, *J* = 6.9 Hz, 4H), 8.79 (s, 2H), 8.62 (d, *J* = 6.6 Hz, 4H), 8.30 (d, *J* = 8.7 Hz, 2H), 8.23 (dd, *J* = 8.6, 1.9 Hz, 2H), 7.68 (d, *J* = 8.4 Hz, 4H), 7.58 (d, *J* = 8.1 Hz, 4H), 2.50 (s, 6H). ^{13}C NMR (126 MHz, D_2O) δ (ppm): 155.79, 143.58, 142.54, 139.56, 135.87, 132.65, 131.94, 130.92, 130.09, 129.67, 126.56, 124.87, 123.29, 20.22. ESI-MS: *m/z* [M-2Cl]$^{2+}$ calcd. for $C_{34}H_{28}N_2^{2+}$: 232.112, found: 232.113.

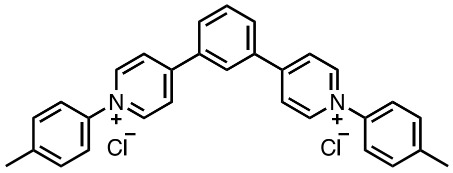

1,3-di(pyridin-4-yl)benzene. 220 mg (Yield: 56 %) ^{1}H NMR (500 MHz, $CDCl_3$) δ (ppm): 8.71 (d, *J* = 6.1 Hz, 4H), 7.87 (t, *J* = 1.8 Hz, 1H), 7.71 (dd, *J* = 7.8, 1.8 Hz, 2H), 7.63 (dd, *J* = 8.4, 7.0 Hz, 1H), 7.56 (d, *J* = 6.2 Hz, 4H).

4,4'-(1,3-phenylene)bis(1-(2,4-dinitrophenyl)pyridin-1-ium) chloride (Ph13DNB). 370 mg (Yield: 95 %) ^{1}H NMR (500 MHz, MeOD) δ (ppm): 9.41 – 9.36 (m, 4H), 9.31 (d, *J* = 2.5 Hz, 2H), 8.95 (dd, *J* = 8.7, 2.5 Hz, 2H), 8.93 – 8.89 (m, 5H), 8.52 (dd, *J* = 7.9, 1.8 Hz, 2H), 8.35 (d, *J* = 8.7 Hz, 2H), 8.06 (t, *J* = 7.9 Hz, 1H). ^{13}C NMR (126 MHz, MeOD) δ (ppm): 159.56, 151.23, 147.28, 144.76, 139.98, 136.77, 133.93, 132.79, 132.65, 131.15, 130.11, 126.58, 123.25. LCMS: *m/z* [M-2Cl]$^{2+}$ calcd. for $C_{28}H_{18}N_6O_8^{2+}$: 283.1, found: 283.2.

4,4'-(1,3-phenylene)bis(1-(p-tolyl)pyridin-1-ium) chloride (Ph13Me). 22 mg (Yield: 95%) ^{1}H NMR (500 MHz, D_2O) δ (ppm): δ 9.15 (d, *J* = 6.5 Hz, 4H), 8.59 (d, *J* = 6.7 Hz, 4H), 8.59 (s, 1H), 8.30 (dd, *J* = 7.9, 1.9 Hz, 2H), 7.96 (t, *J* = 7.8 Hz, 1H), 7.69 (d, *J* = 8.2 Hz, 4H), 7.59 (d, *J* = 8.2 Hz, 4H), 2.51 (s, 6H). ^{13}C NMR (126 MHz, D_2O) δ (ppm): 156.07, 144.15, 142.52, 139.92, 135.38, 131.59, 131.04, 130.89, 127.79, 125.36, 123.58, 20.26. ESI-MS: *m/z* [M-2Cl]$^{2+}$ calcd. for $C_{30}H_{26}N_2^{2+}$: 207.104, found: 207.105.

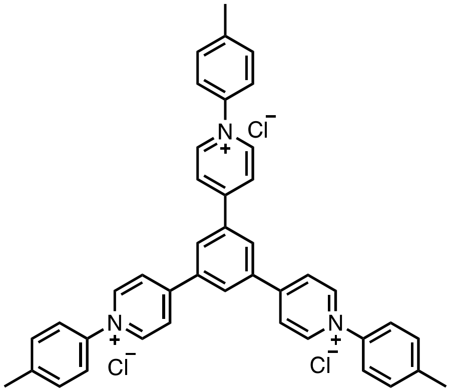

1,3,5-tri(pyridin-4-yl)benzene. 30 mg (Yield: 10 %) ^{1}H NMR (500 MHz, $CDCl_3$) δ (ppm): 8.80 – 8.72 (m, 6H), 7.92 (s, 3H), 7.64 – 7.57 (m, 6H). ^{13}C NMR (126 MHz, $CDCl_3$) δ (ppm): 150.74, 147.49, 140.55, 126.49, 121.95.

4,4',4''-(benzene-1,3,5-triyl)tris(1-(2,4-dinitrophenyl)pyridin-1-ium) chloride (Ph135DNB). 25 mg (Yield: 30 %) ^{1}H NMR (500 MHz, MeOD) δ (ppm): 9.51 (d, *J* = 7.1 Hz, 6H), 9.34 (d, *J* = 2.5 Hz, 3H), 9.22 (s, 3H), 9.10 (d, *J* = 7.1 Hz, 6H), 8.98 (dd, *J* = 8.7, 2.5 Hz, 3H), 8.37 (d, *J* = 8.7 Hz, 3H). ^{13}C NMR (126 MHz, MeOD) δ (ppm): 158.55, 151.34, 147.60, 144.75, 139.92, 138.37, 133.68, 132.58, 131.22, 127.16, 123.33. LCMS: *m/z* $[M\text{-}3Cl]^{3+}$ calcd. for $C_{39}H_{24}N_9O_{12}^{3+}$: 270.1, found: 270.2.

4,4',4''-(benzene-1,3,5-triyl)tris(1-(p-tolyl)pyridin-1-ium) chloride (Ph135Me). 10 mg (Yield: 90%) ^{1}H NMR (500 MHz, D_2O) δ (ppm): δ 9.25 (d, *J* = 6.6 Hz, 6H), 8.82 (s, 3H), 8.71 (d, *J* = 6.6 Hz, 6H), 7.72 (d, *J* = 8.3 Hz, 6H), 7.61 (d, *J* = 8.2 Hz, 6H), 2.52 (s, 9H). ^{13}C NMR (126 MHz, D_2O) δ (ppm): 155.12, 144.44, 142.67, 139.91, 136.96, 130.92, 130.88, 125.78, 123.61, 20.26. ESI-MS: *m/z* $[M\text{-}3Cl]^{3+}$ calcd. for $C_{42}H_{36}N_3^{3+}$: 194.096, found: 194.096; $[M\text{-}3Cl]^{2+}$ calcd. for $C_{42}H_{36}N_3^{2+}$: 291.145, found: 291.144.

SI-3 Diffusion coefficients determined by DOSY

The ^{1}H DOSY experiments were carried out using a modified version of the Bruker sequence ledbpgp2s involving, typically, 32 scans over 16 steps of gradient variation from 10% to 80% of the maximum gradient. Diffusion coefficients were evaluated in Dynamic Centre (a standard Bruker software) and determined by fitting the intensity decays according to the following equation:

$$I = I_o\, e^{[-D\gamma^2 g^2 \delta^2 (\Delta - \delta/3)]}$$

where I and I_o represent the signal intensities in the presence and absence of gradient pulses respectively, D is the diffusion coefficient, γ = 26753 rad/s/Gauss is the ^{1}H gyromagnetic ratio, δ = 2.4 ms is duration of the gradient pulse, Δ = 100 ms is the total diffusion time and g is the applied gradient strength. Monte Carlo simulation method was used for the error estimation of fitting parameters with a confidence level of 95%.

Table S1. Diffusion coefficient of different CB[7] and CB[8] complexes obtained from DOSY measurements in D_2O at 298 K.

Species	*Diffusion Coefficient ($10^{-10} \cdot m^2/s$)*					
	G	G_1-CB[7]$_2$	G_2-CB[8]$_2$	G_1-CB[7]$_3$	G_2-CB[8]$_3$	G_1-CB[8]$_1$
Ant910Me	3.56 ± 0.04	2.20 ± 0.01	2.04 ± 0.01	-	-	-
Ant15Me	3.59 ± 0.02	2.15 ± 0.01	1.98 ± 0.01	-	-	-
Ant14Me	3.49 ± 0.03	2.16 ± 0.01	1.95 ± 0.01	N.D.	1.85 ± 0.01	-
Np27Me	3.89 ± 0.01	2.18 ± 0.01	2.00 ± 0.01	-	-	-
Np14Me	3.85 ± 0.01	2.20 ± 0.01	1.98 ± 0.01	-	-	-
Np15Me	3.86 ± 0.02	2.18 ± 0.01	2.00 ± 0.01	-	-	-
Ph14Me	4.38 ± 0.02	2.20 ± 0.01	2.06 ± 0.01	-	-	-
Ph13Me	4.10 ± 0.02	2.25 ± 0.01	2.07 ± 0.01	-	-	-
Ph135Me	3.18 ± 0.02	N.D.	N.D.	1.89 ± 0.01	1.79 ± 0.01	-
VNMe$_2$	-	-	2.01 ± 0.01	-	-	-
dzpy	-	-	-	-	-	3.04 ± 0.01
CB[8]	-	-	-	-	-	3.11 ± 0.01
Np14H	4.05 ± 0.02	2.24 ± 0.01	2.06 ± 0.01	-	-	-
Np14NH$_2$	3.91 ± 0.03	2.26 ± 0.01	2.02 ± 0.01	-	-	-
Np14Me	3.85 ± 0.01	2.20 ± 0.01	1.98 ± 0.01	-	-	-
Np14OMe	3.83 ± 0.01	2.18 ± 0.03	1.97 ± 0.01	-	-	-
Np14SMe	3.73 ± 0.02	2.17 ± 0.01	1.99 ± 0.01	-	-	-
Np14NMe$_2$	3.53 ± 0.01	2.15 ± 0.01	2.00 ± 0.01	-	-	-

SI-4 Complexation of Ant910Me with CB[7] and CB[8]

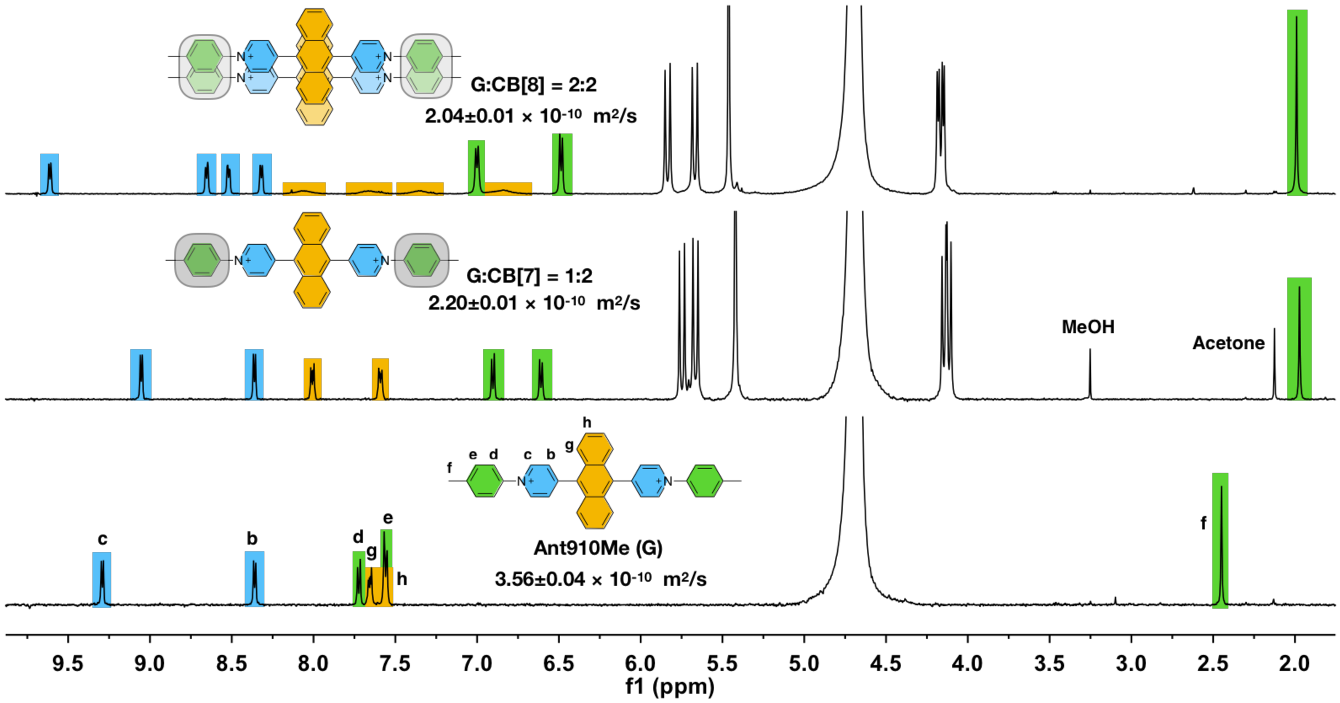

Figure S1. ^{1}H NMR spectra of **Ant910Me** (G), G_1-CB[7]$_2$, and G_2-CB[8]$_2$ complexes in D_2O at 298 K.

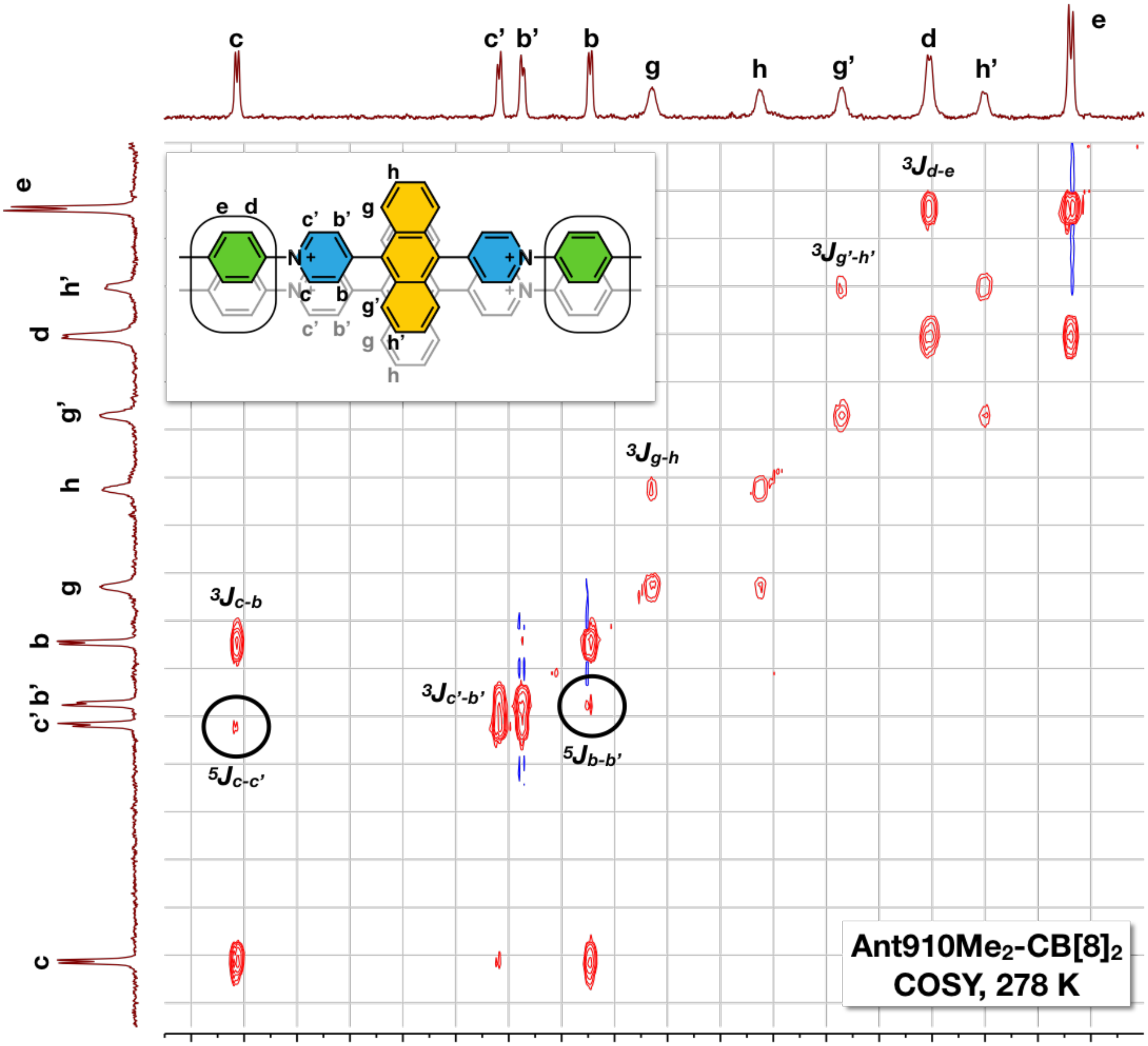

Figure S2. ^1H-^1H COSY of **Ant910Me**$_2$-CB[8]$_2$ complex in D_2O at 278 K.

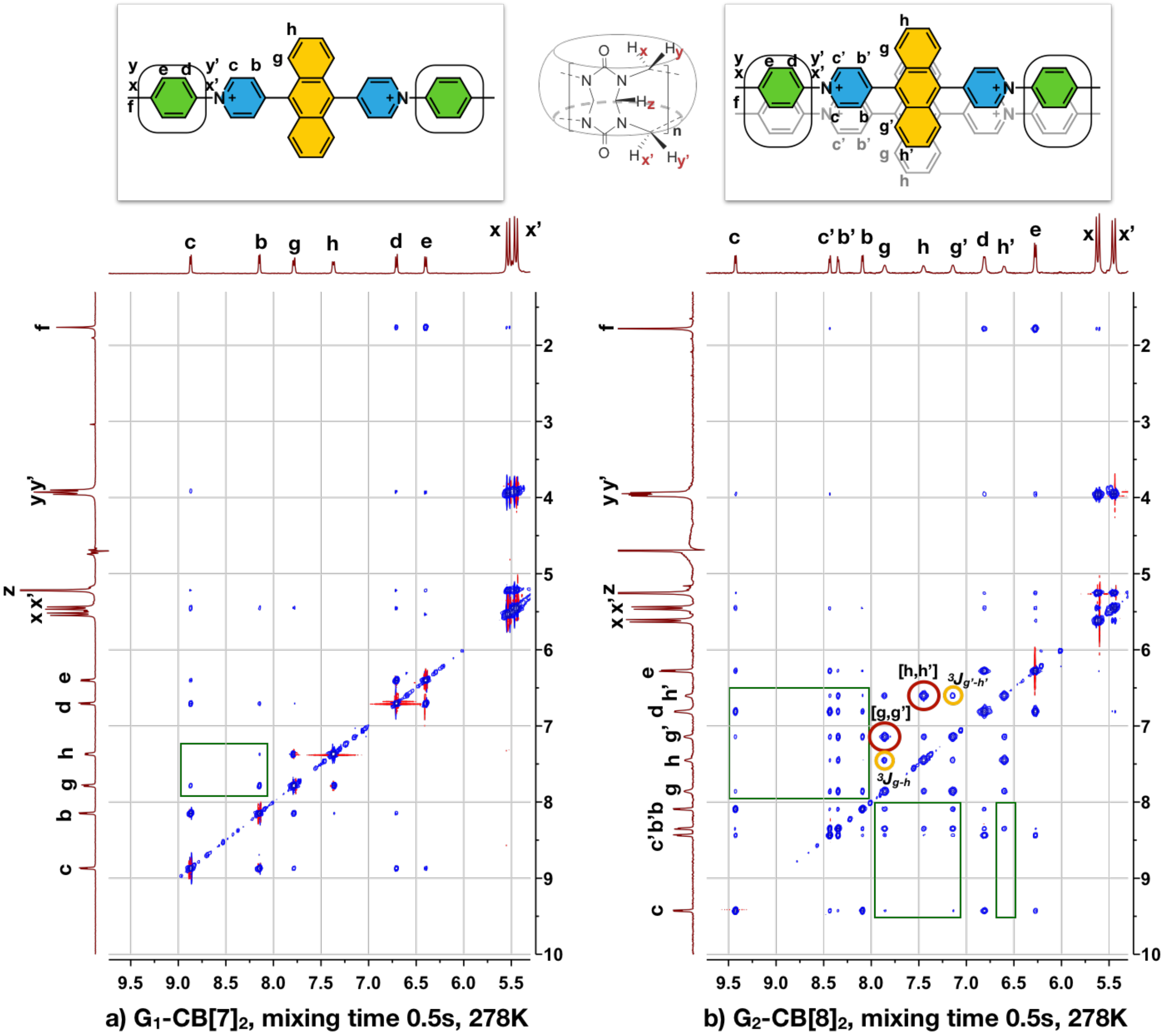

Figure S3. NOESY spectra of **Ant910Me** in its a) G_1-CB[7]$_2$ and b) G_2-CB[8]$_2$ complex with uniform guest concentration in D_2O at 278 K. Green rectangles highlight the cross-correlation between $H^{b,b',c,c'}$ and $H^{g,g',h,h'}$. Cross-correlations between H^g and $H^{g'}$ as well as those between H^h and $H^{h'}$ are more intense than correlations caused by $^3J_{H-H}$ coupling for H^g-H^h and $H^{g'}$ -$H^{h'}$, which implies the presence of a dynamic interconversion between two discrete states within the CB[8] complex.

SI-5 Complexation of Np27Me with CB[7] and CB[8]

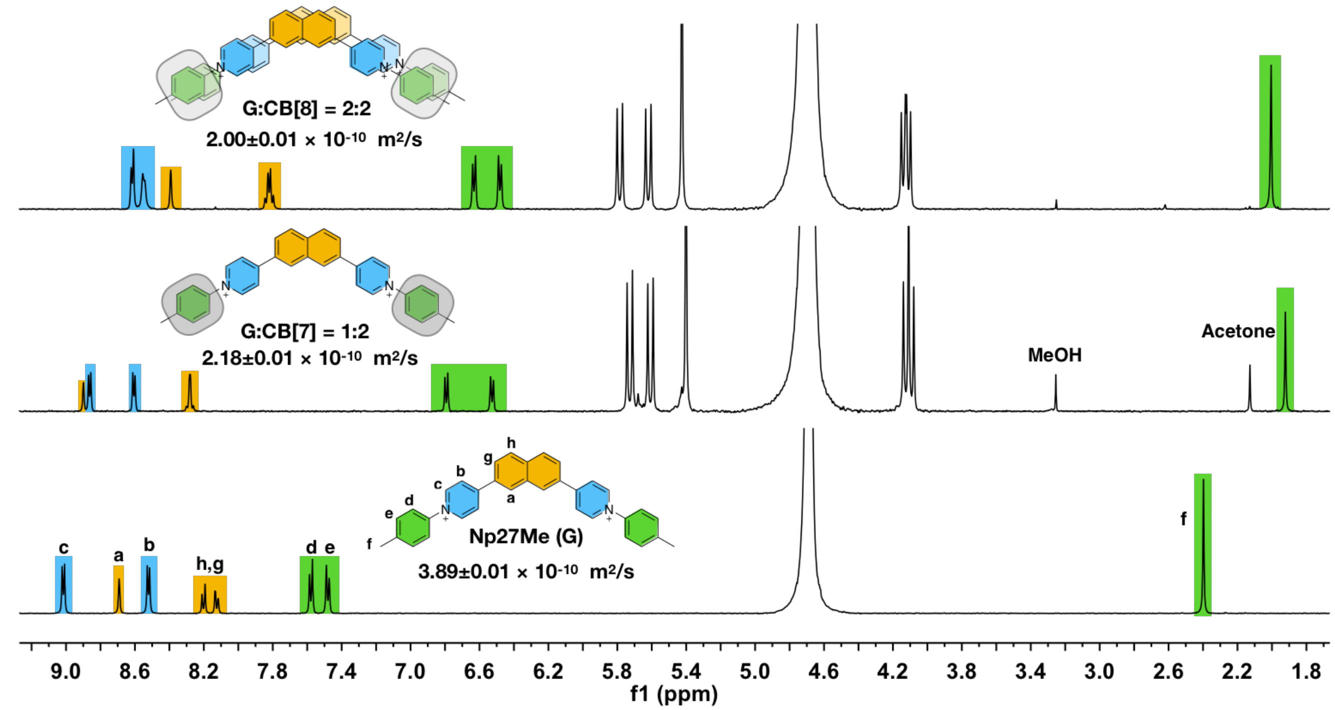

Figure S4. ^{1}H NMR spectra of **Np27Me** (G), G_1-$CB[7]_2$, and G_2-$CB[8]_2$ complexes in D_2O at 298 K.

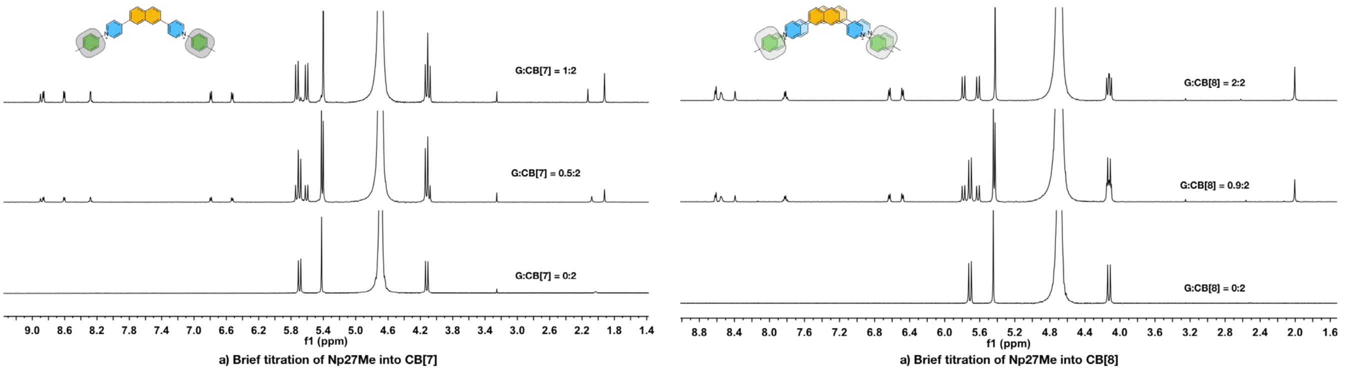

Figure S5. ^{1}H NMR titration of **Np27Me** (G) into a) CB[7] and b) CB[8] in D_2O at 298 K showing a quantitative binding manner. CB[7] and CB[8] don't bind to the fluorophore core even if they are in excess.

SI-6 Complexation of Np14Me with CB[7] and CB[8]

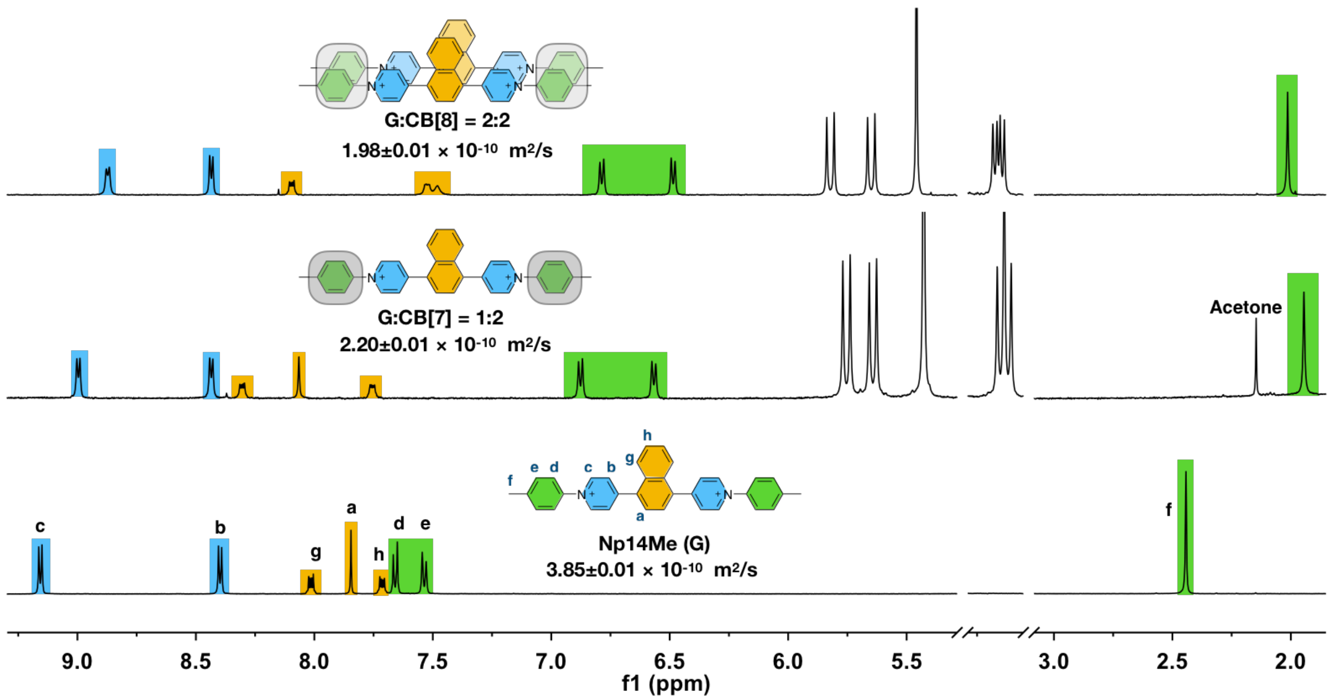

Figure S6. ^{1}H NMR spectra of **Np14Me** (G), G_1-$CB[7]_2$, and G_2-$CB[8]_2$ complexes in D_2O at 298 K.

SI-7 Complexation of Np15Me with CB[7] and CB[8]

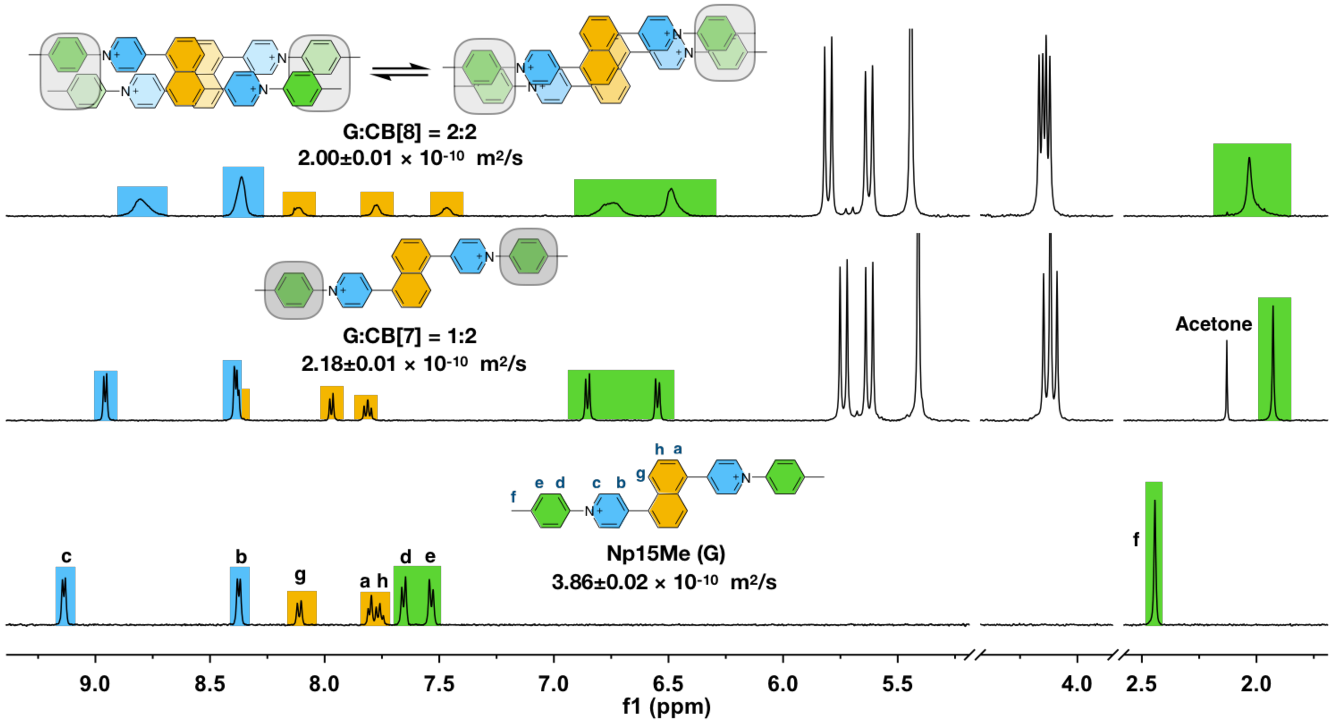

Figure S7. ^{1}H NMR spectra of **Np15Me** (G), G_1-$CB[7]_2$, and G_2-$CB[8]_2$ complexes in D_2O at 298 K.

SI-8 Complexation of Ant15Me with CB[7] and CB[8]

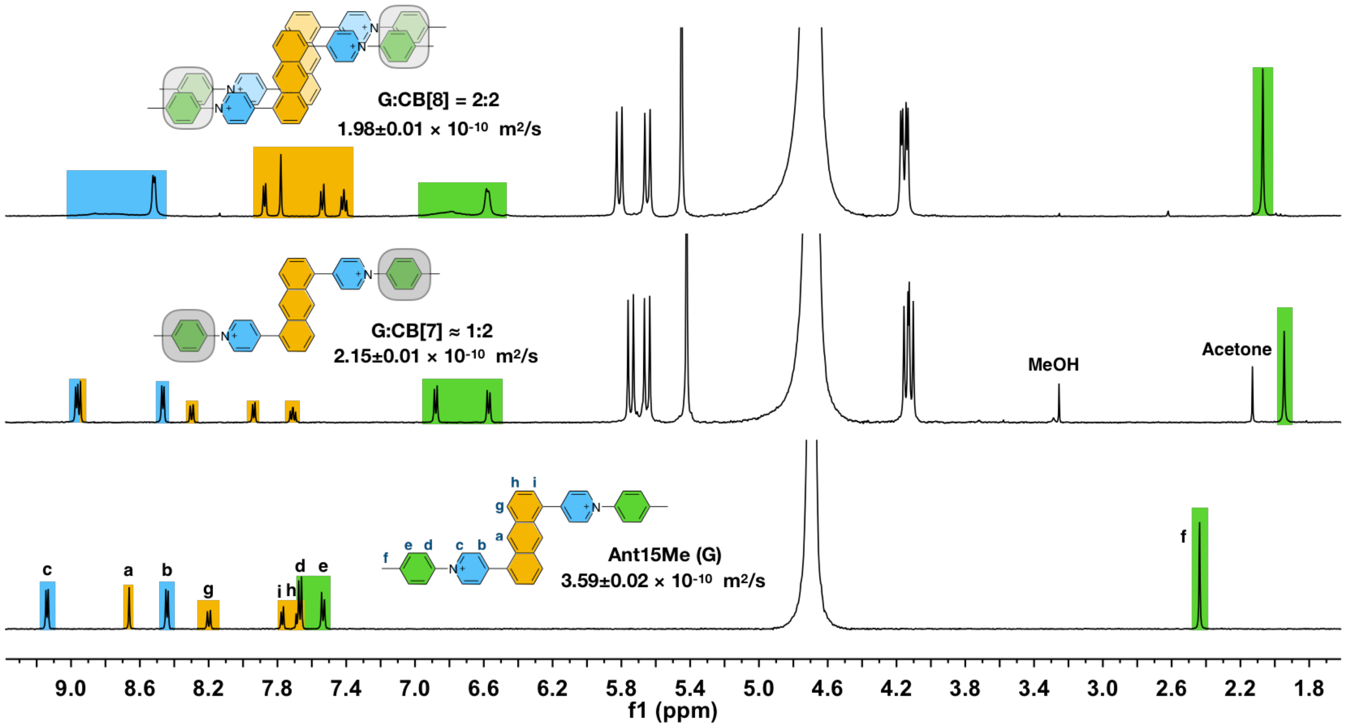

Figure S8. ^{1}H NMR spectra of **Ant15Me** (G), G_1-CB[7]$_2$, and G_2-CB[8]$_2$ complexes in D_2O at 298 K.

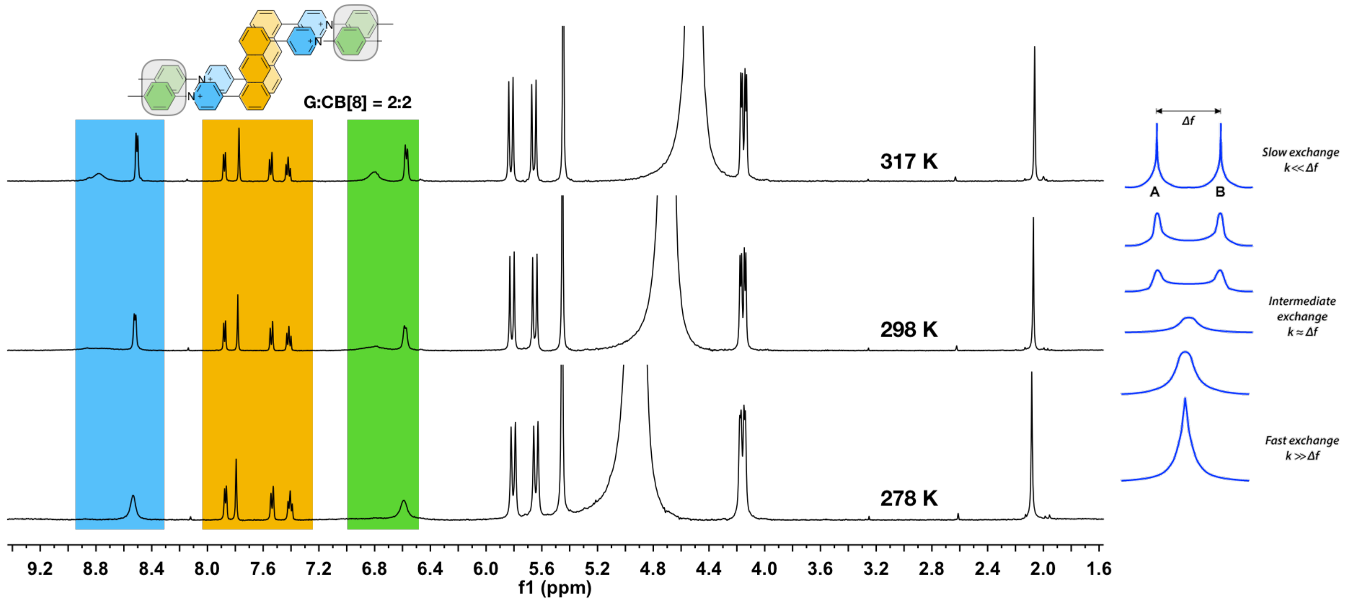

Figure S9. VT-NMR of **Ant15Me**$_2$-CB[8]$_2$ complex in D_2O from 278 K to 317 K showing a retarded rotation of tolyl pyridinium moieties in this complex.

SI-9 Complexation of Ant14Me with CB[7] and CB[8]

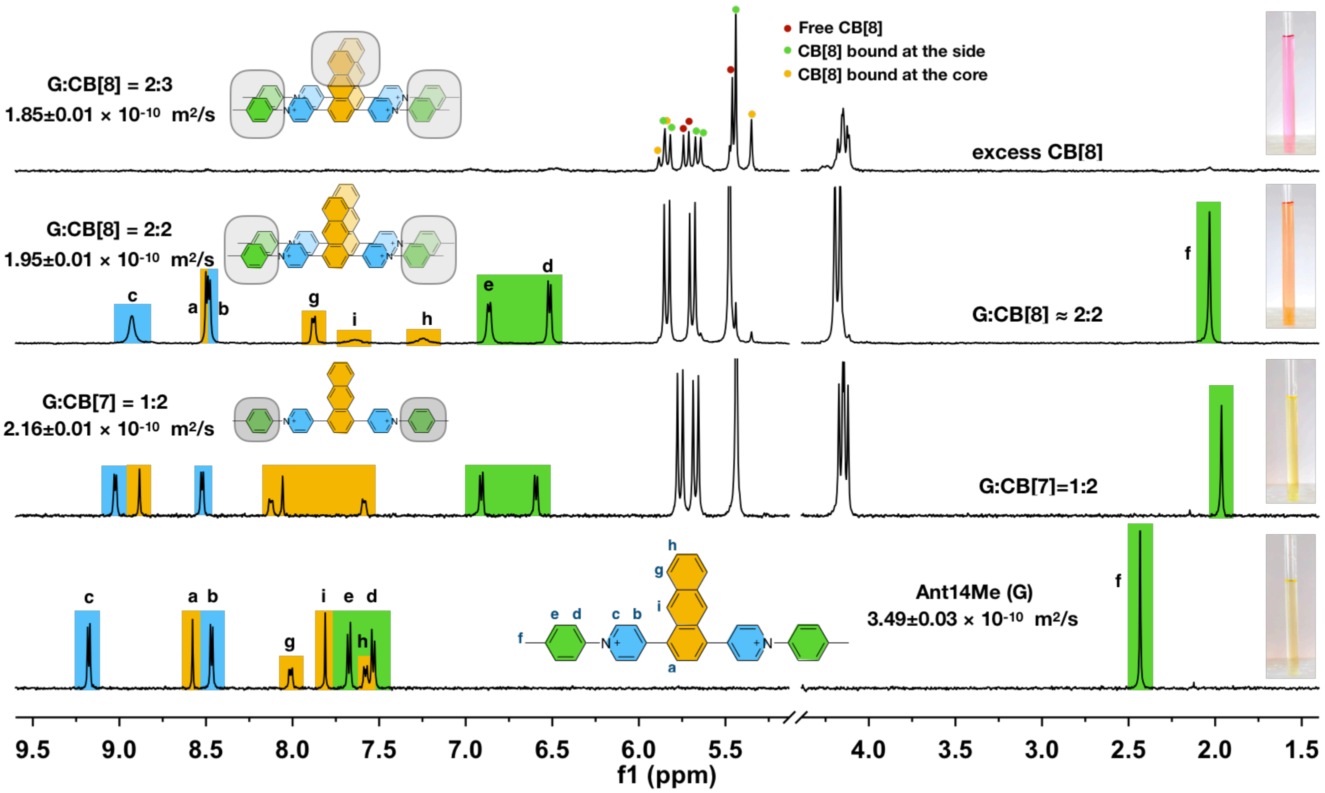

Figure S10. ^{1}H NMR spectra of **Ant14Me** (G), G_1-CB[7]$_2$, G_2-CB[8]$_2$ and G_2-CB[8]$_3$ (with excess of CB[8]) complexes in D_2O at 298 K.

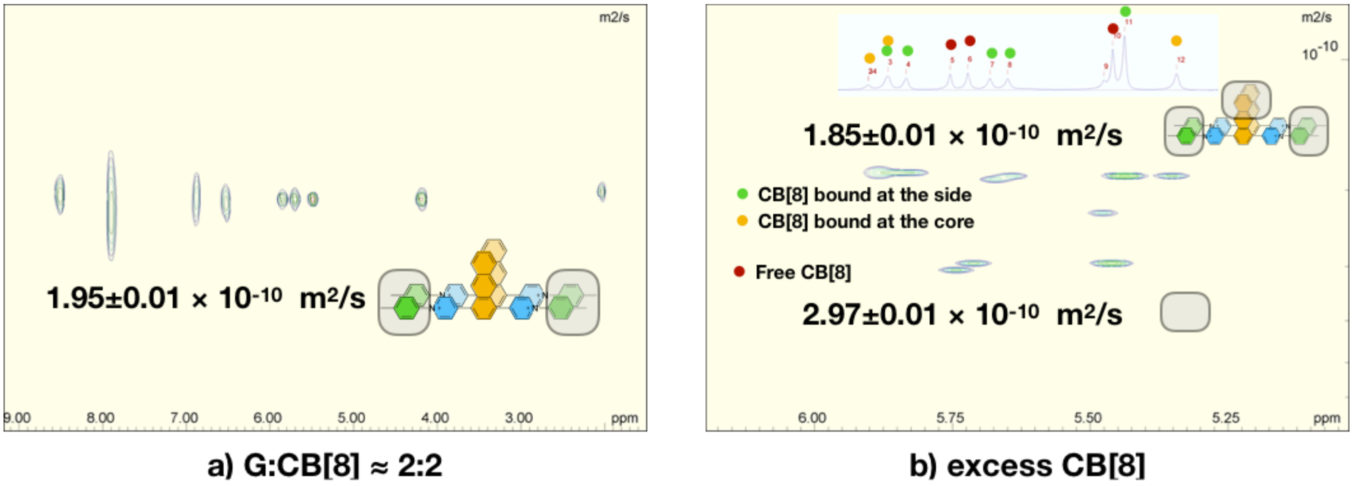

Figure S11. DOSY result of **Ant14Me** (G) in presence of a) around 1 equiv. of CB[8] or b) excess of CB[8] in D_2O at 298 K.

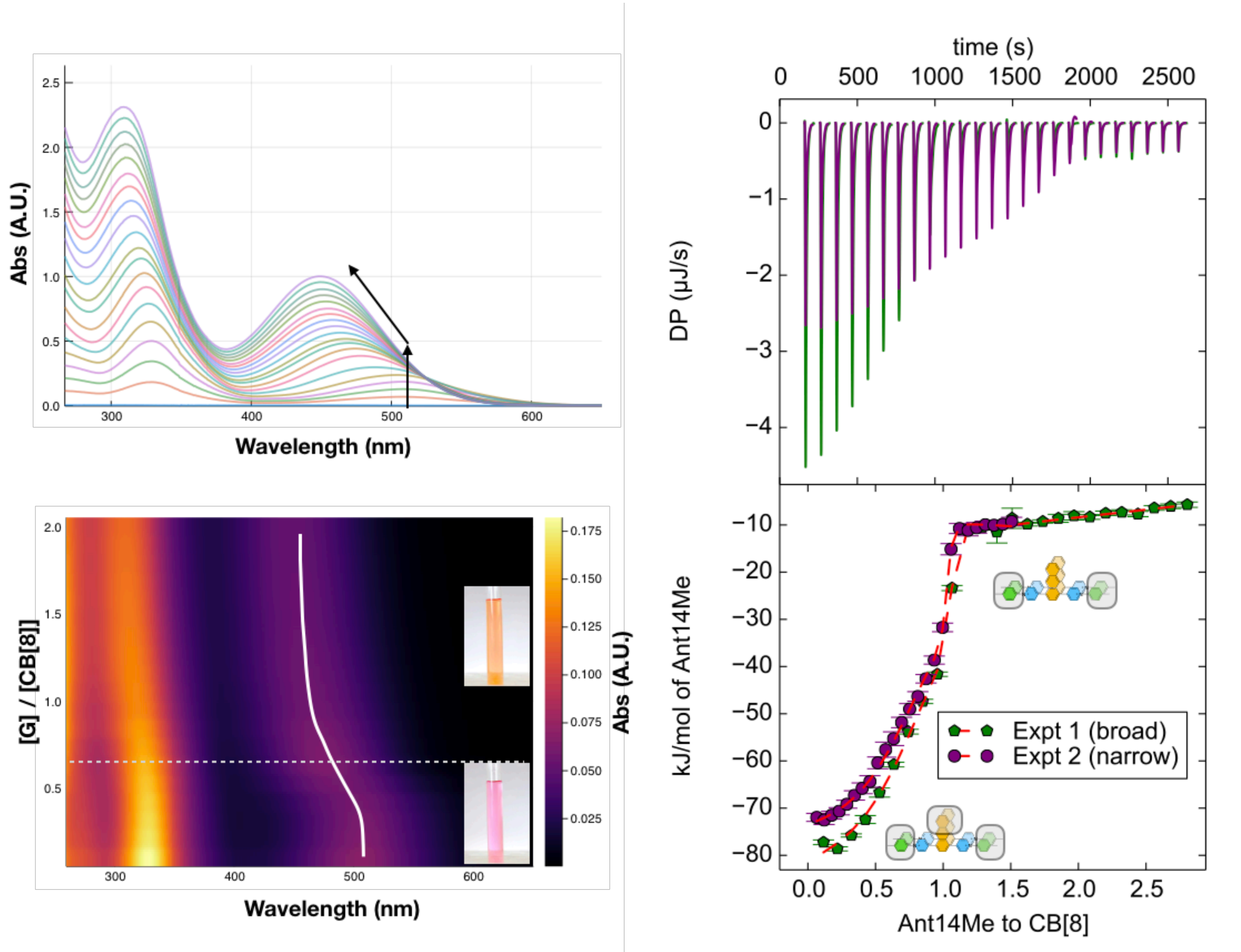

a) UV-Vis titration of Ant14Me into CB[8] **b) ITC of Ant14Me into CB[8]**

Figure S12. Titration of **Ant14Me** into CB[8] monitored by a) UV-Vis in water and b) ITC in 10 mM sodium phosphate buffer (pH=7) at 298 K. Both titrations show a first turning point at a G:CB[8] ratio of around 0.67 corresponding to the formation of G_2-CB$[8]_3$ complex. Two ITC curves with different titration windows can be perfectly fitted (global fitting) by taking account of the competitive binding of *p*-tolyl pyridinium and *1,4*-anthracenyl moieties with CB[8]. Thermodynamic data for the formation of (*p*-tolyl pyridinium)$_2$-CB[8] motif are *dG* = -11.8 kcal/mol, *dH* = -11.9 kcal/mol, *TdS* = -0.1 kcal/mol. Thermodynamic data for the formation of (*1,4*-anthracenyl)$_2$-CB[8] motif are *dG* = -6.8 kcal/mol, *dH* = -10.2 kcal/mol, *TdS* = -3.3 kcal/mol. The formation of (*1,4*-anthracenyl)$_2$-CB[8] is entropically unfavorable.

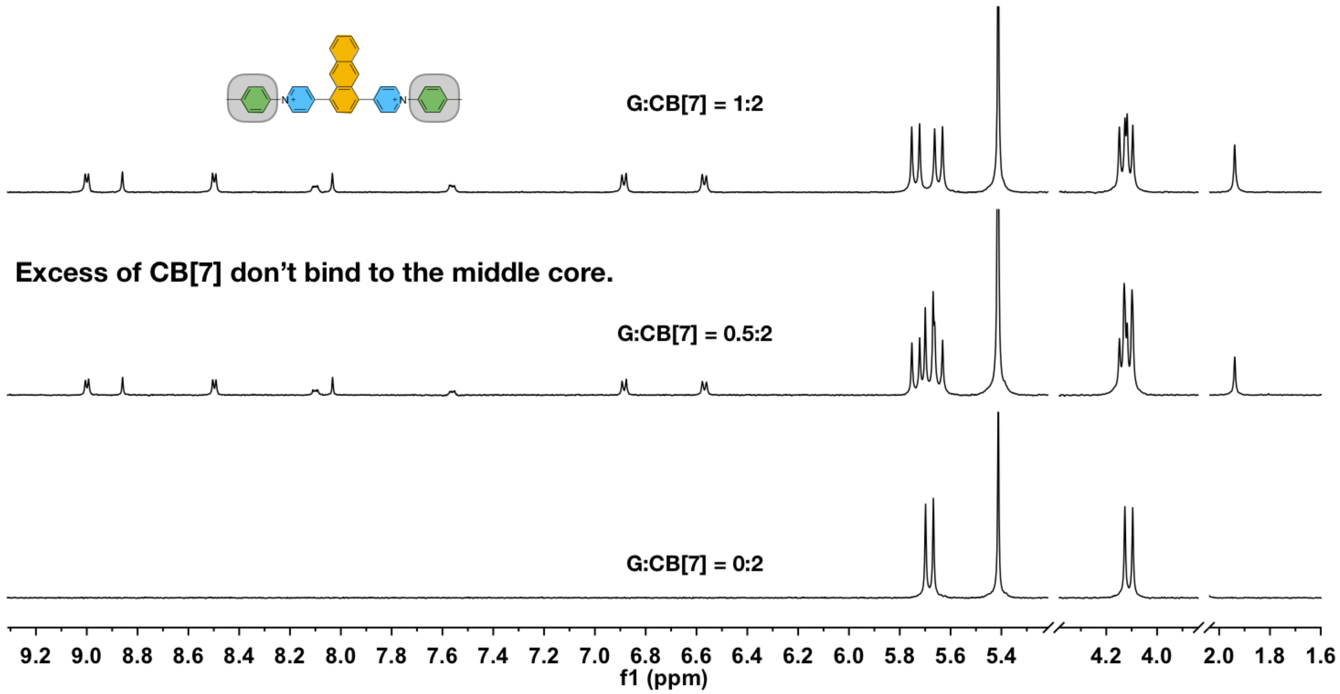

Figure S13. ^{1}H NMR titration of **Ant14Me** (G) into CB[7] in D_2O at 298 K showing a quantitative binding manner. In contrast to CB[8], excess of CB[7] does not bind to the fluorophore core.

SI-10 Complexation of Ph14Me with CB[7] and CB[8]

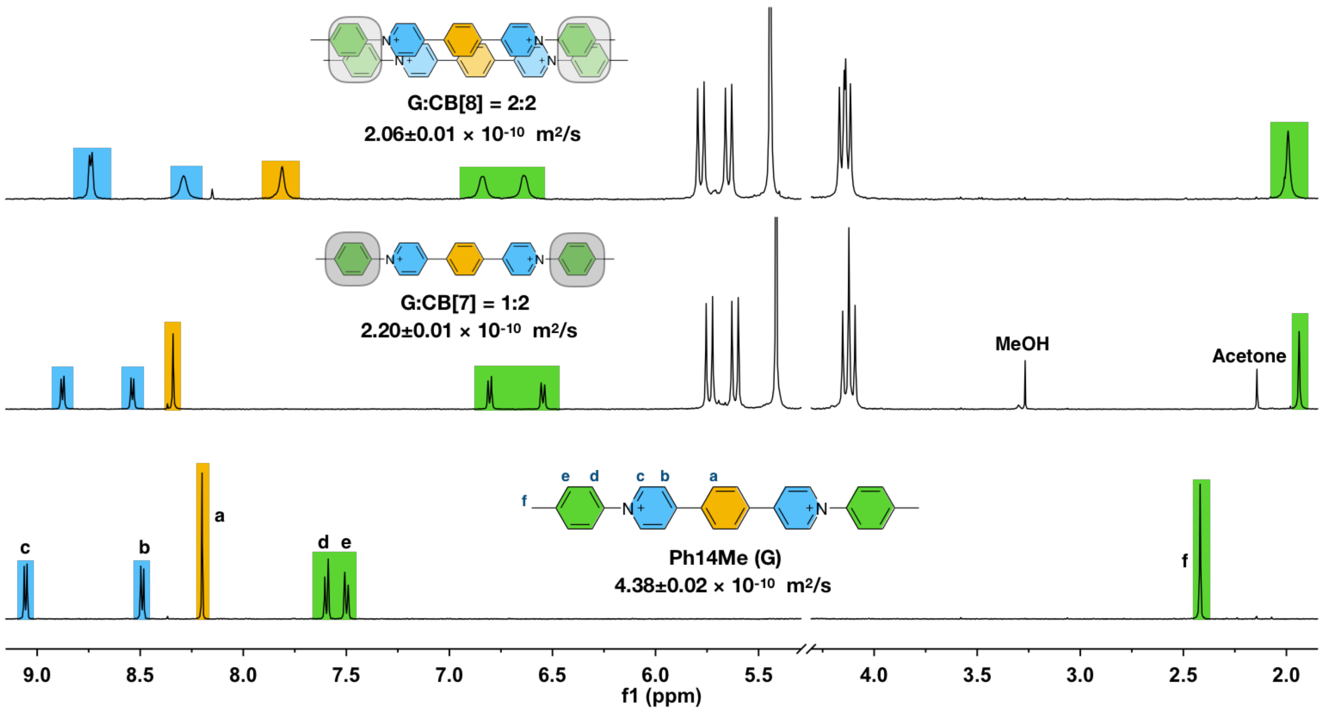

Figure S14. ^{1}H NMR spectra of **Ph14Me** (G), G_1-CB[7]$_2$, and G_2-CB[8]$_2$ complexes in D_2O at 298 K.

SI-11 Complexation of Ph13Me with CB[7] and CB[8]

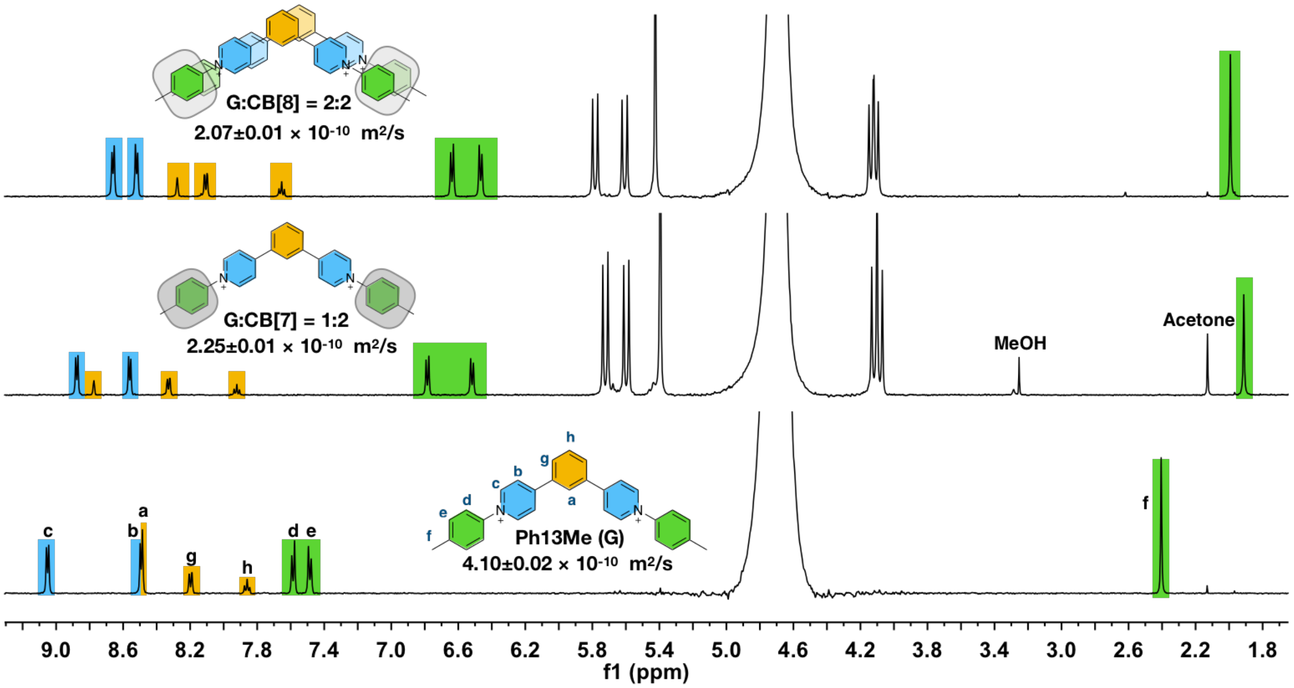

Figure S15. ^{1}H NMR spectra of **Ph13Me** (G), G_1-CB[7]$_2$, and G_2-CB[8]$_2$ complexes in D_2O at 298 K.

SI-12 Complexation of Ph135Me with CB[7] and CB[8]

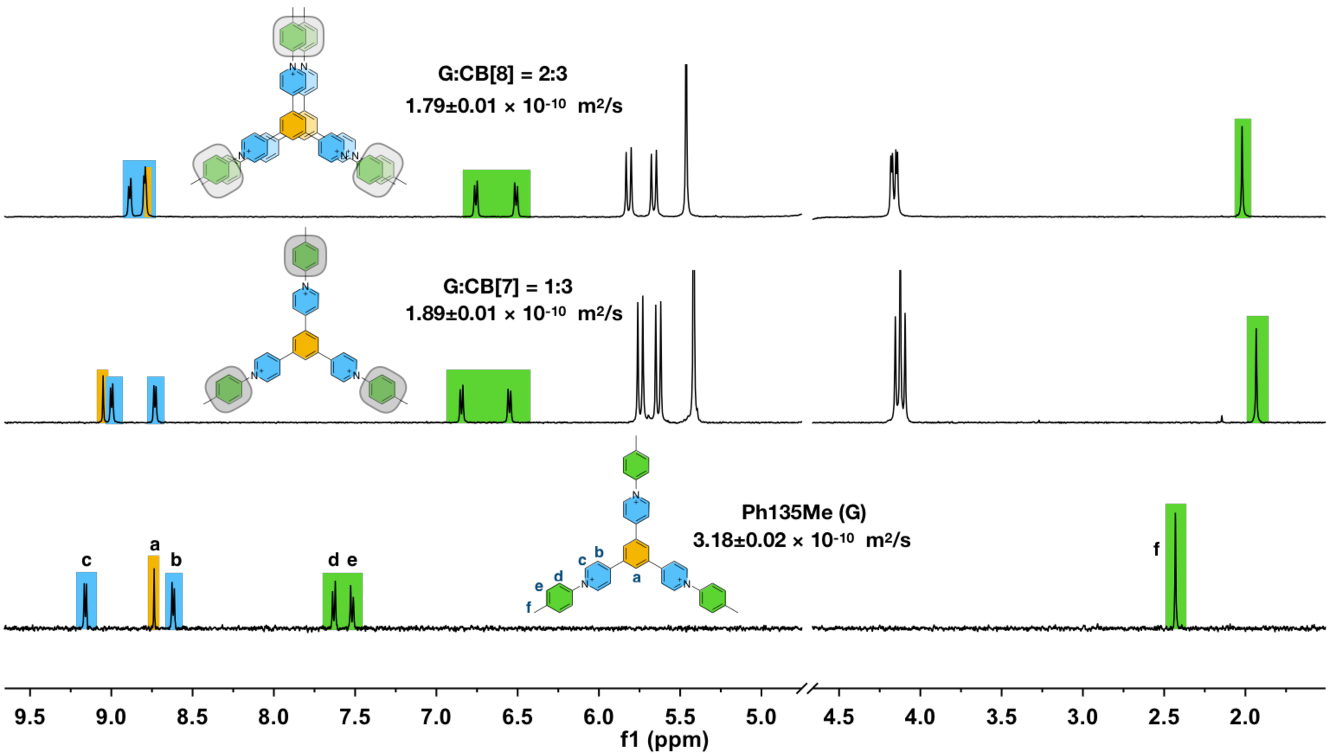

Figure S16. ^{1}H NMR spectra of **Ph135Me** (G), G_1-CB[7]$_3$, and G_2-CB[8]$_3$ complexes in D_2O at 298 K.

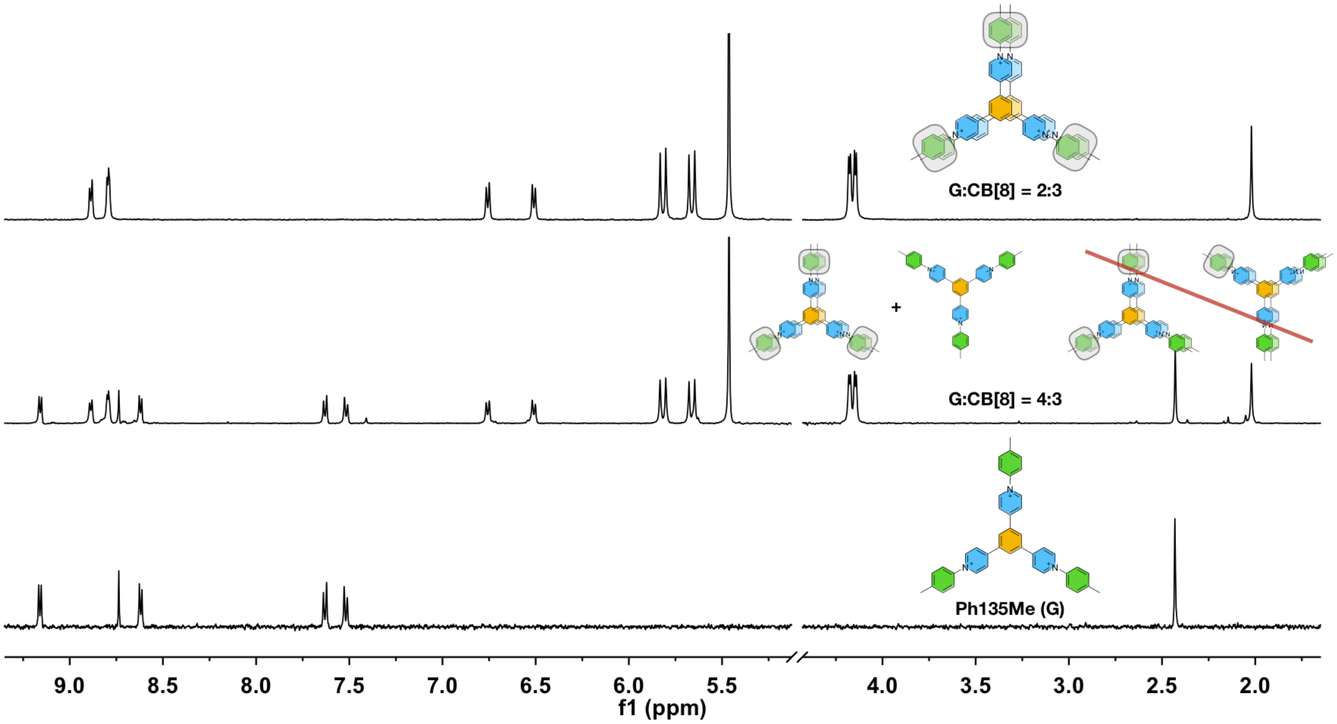

Figure S17. Excess of **Ph135Me** (G:CB[8] = 4:3) does not transform G_2-$CB[8]_3$ complexes into statistic complexes.

SI-13 VT-NMR study of the Ant910Me$_2$-CB[8]$_2$ complex

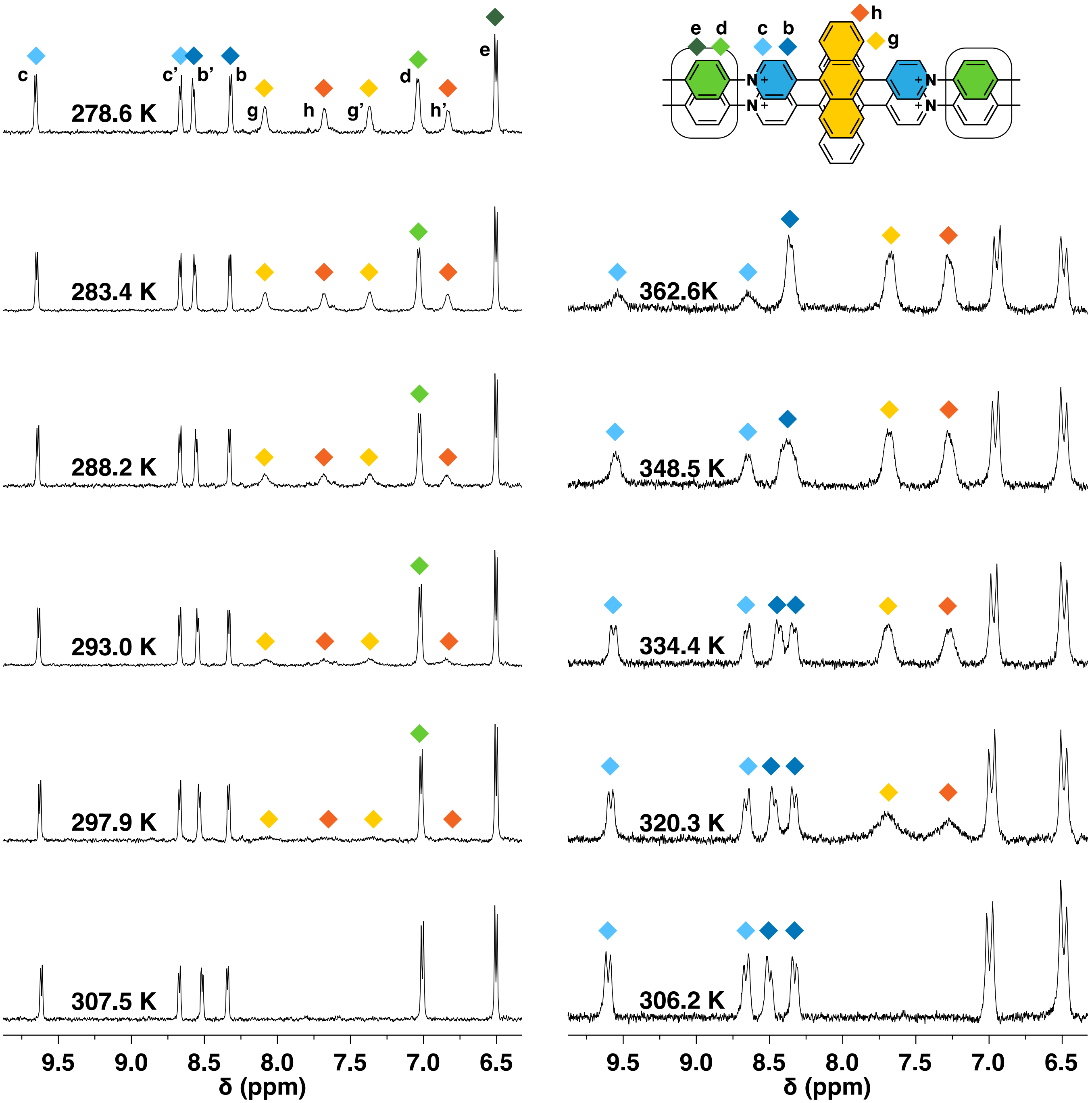

a) High-field VT-NMR (500 MHz)   b) Low-field VT-NMR (200 MHz)

Figure S18. VT-NMR of Ant910Me$_2$-CB[8]$_2$ by a) high-field (500 MHz) and b) low-field (200 MHz) NMR in D_2O. The temperature of 500 MHz and 200 MHz spectrometers is calibrated by MeOD (D, 99.8 %) and ethylene glycol (80 % in DMSO-D6), respectively.

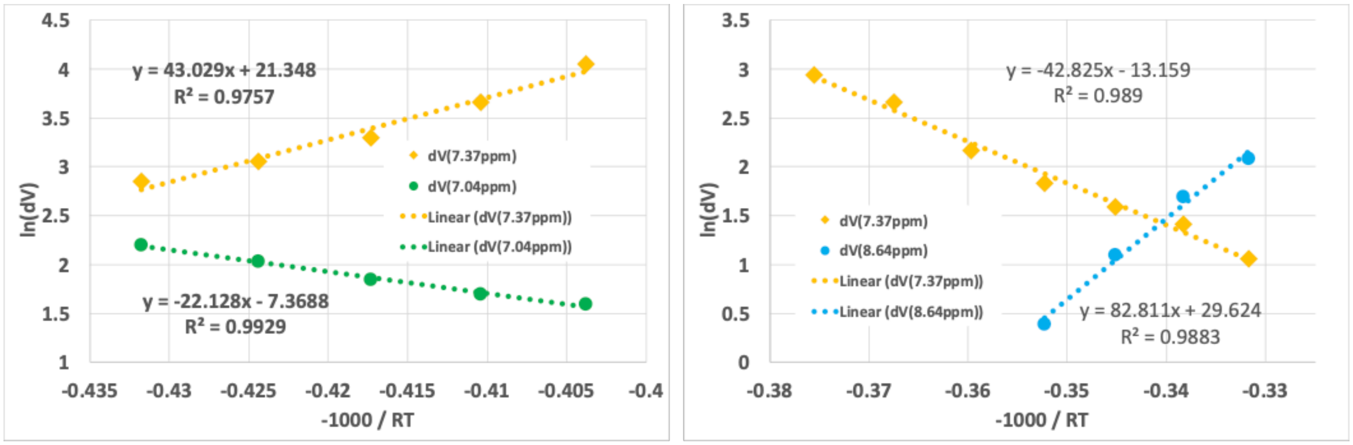

a) linewidth (dV) from 500 MHz **b) linewidth (dV) from 200 MHz**

In the slow-exchange limit: $\ln(dV) = -E_a/RT+A$

E_a(Ant) = 43 kJ/mol **E_a(Pyr) = 83 kJ/mol**

In the fast-exchange limit: $\ln(dV) = E_a/RT+B$

E_a(Tolyl) = 22 kJ/mol **E_a(Ant) = 43 kJ/mol**

Figure S19. Line-broadening analysis of VT-NMR data from a) high-field (500 MHz) and b) low-field (200 MHz) spectra (Figure. S18). The signals of anthracenyl moieties fall in the slow limit in high-field spectra and in the fast limit in low-field spectra. Therefore, a reference linewidth of 11.7 Hz was chosen for the line-broadening analysis in low-field spectra in order to unify the activation energy for anthracenyl moieties in both spectrometers.

SI-14 Transient absorption of G_1-CB[7]$_2$ and G_2-CB[8]$_2$ complex of Ant910Me

Solution samples were prepared in a 2 mm path length glass cuvette and the steady state absorbance of testing samples is shown in Figure S20.

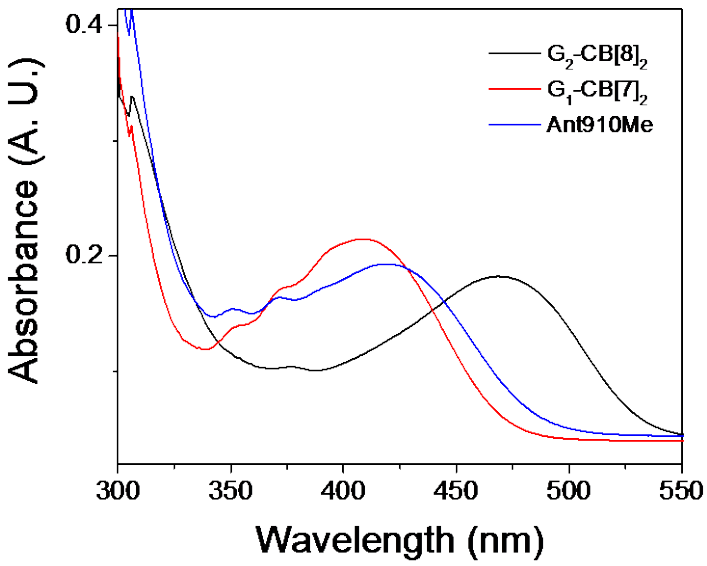

Figure S20. Steady state absorbance showing the actual OD of the fsTA and nsTA samples.

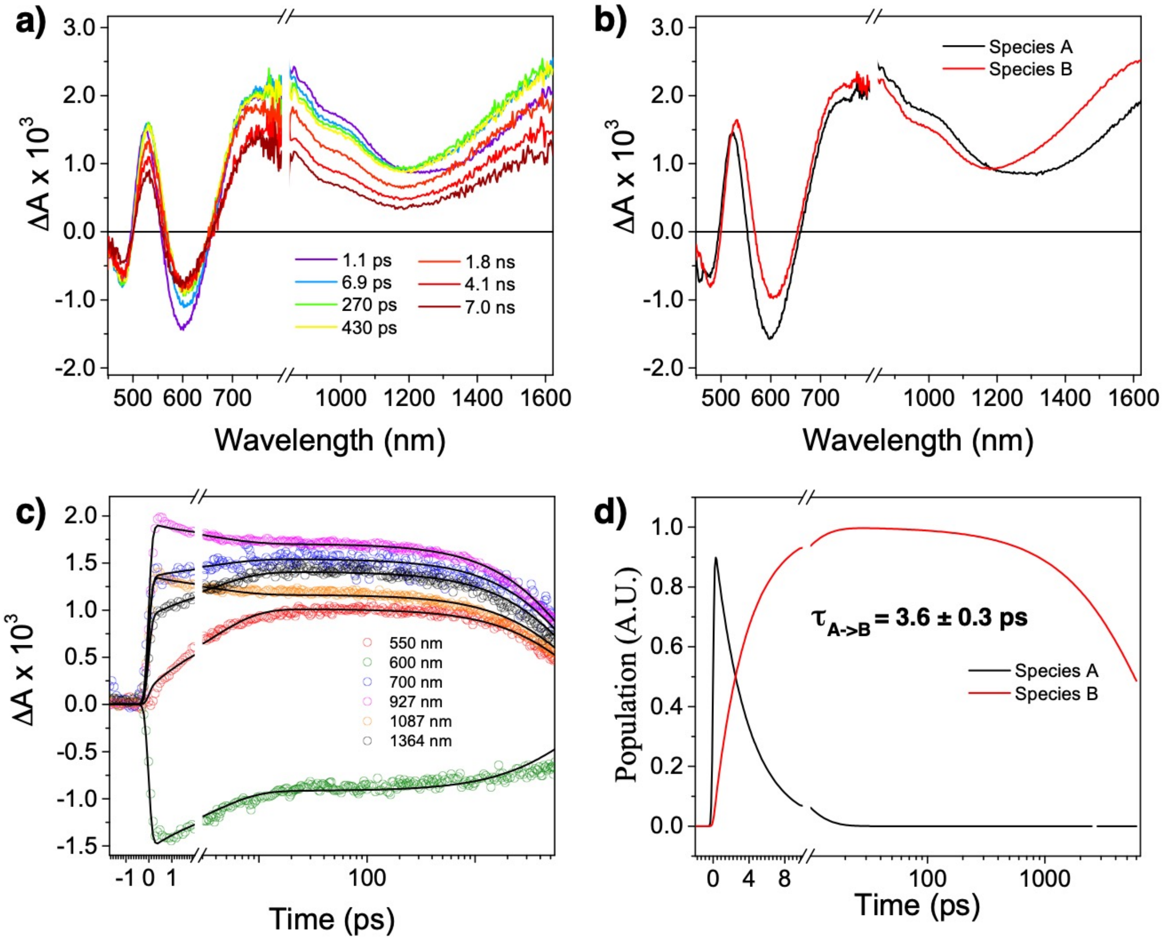

Figure S21. (a) fsTA spectra (Vis-NIR) of G_2-CB[8]$_2$ complex of **Ant910Me** in D_2O (in air) at 298 K following excitation at 414 nm (1 μJ/pulse); (b) species associated spectra from fitting the raw data using the kinetic model A → B → GS; (c) wavelength fitting, and (d) model population kinetics.

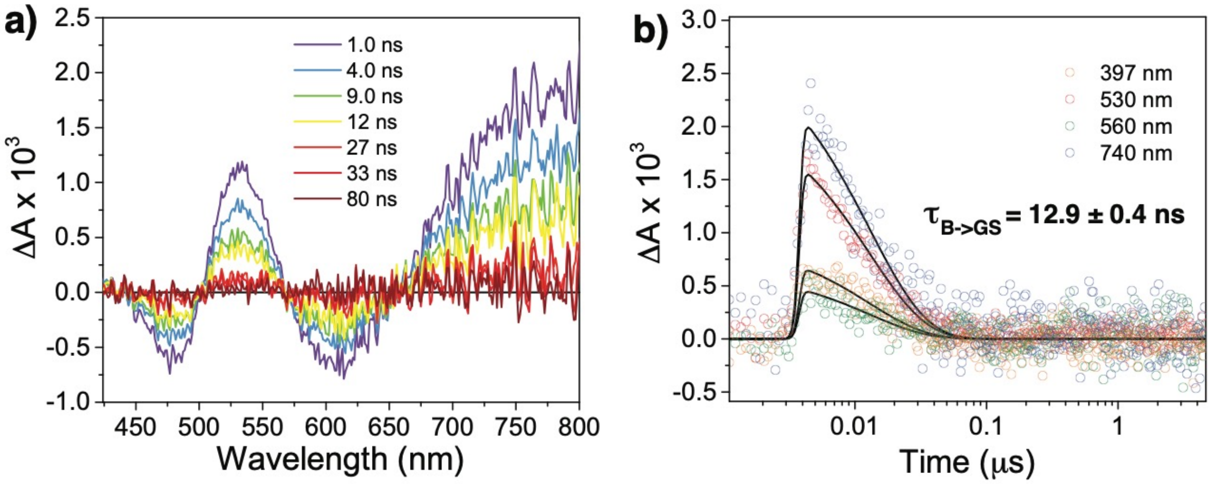

Figure S22. (a) nsTA spectra of G_2-CB[8]$_2$ complex of **Ant910Me** in D_2O (in air) at 298 K following excitation at 414 nm (1μJ/pulse) and (b) kinetic fitting at selected wavelength using mono-exponential fit.

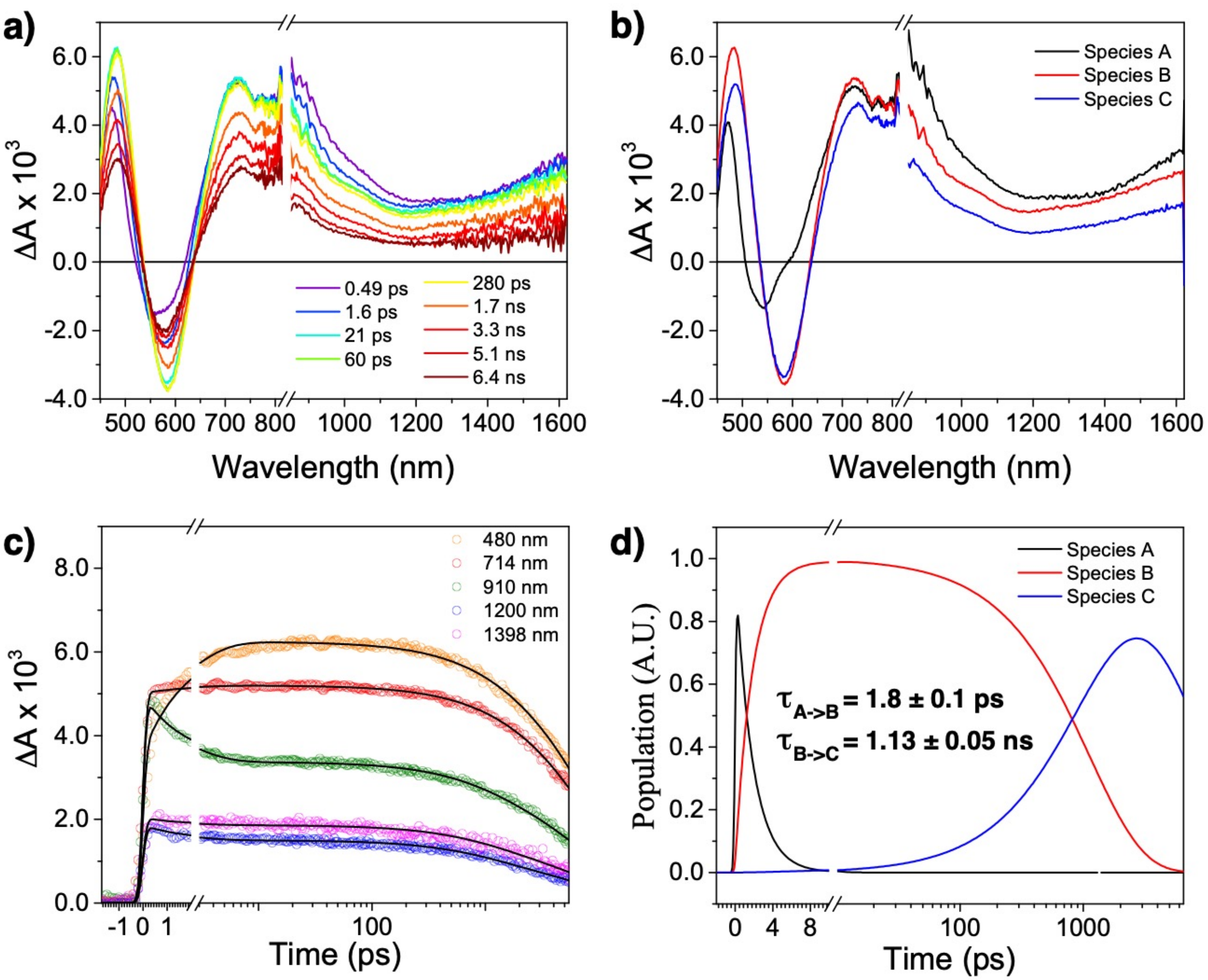

Figure S23. (a) fsTA spectra (Vis-NIR) of G_1-CB[7]$_2$ complex of **Ant910Me** in D_2O (in air) at 298 K following excitation at 414 nm (1 μJ/pulse); (b) species associated spectra from fitting the raw data using the kinetic model A → B → C → GS; (c) wavelength fitting, and (d) model population kinetics.

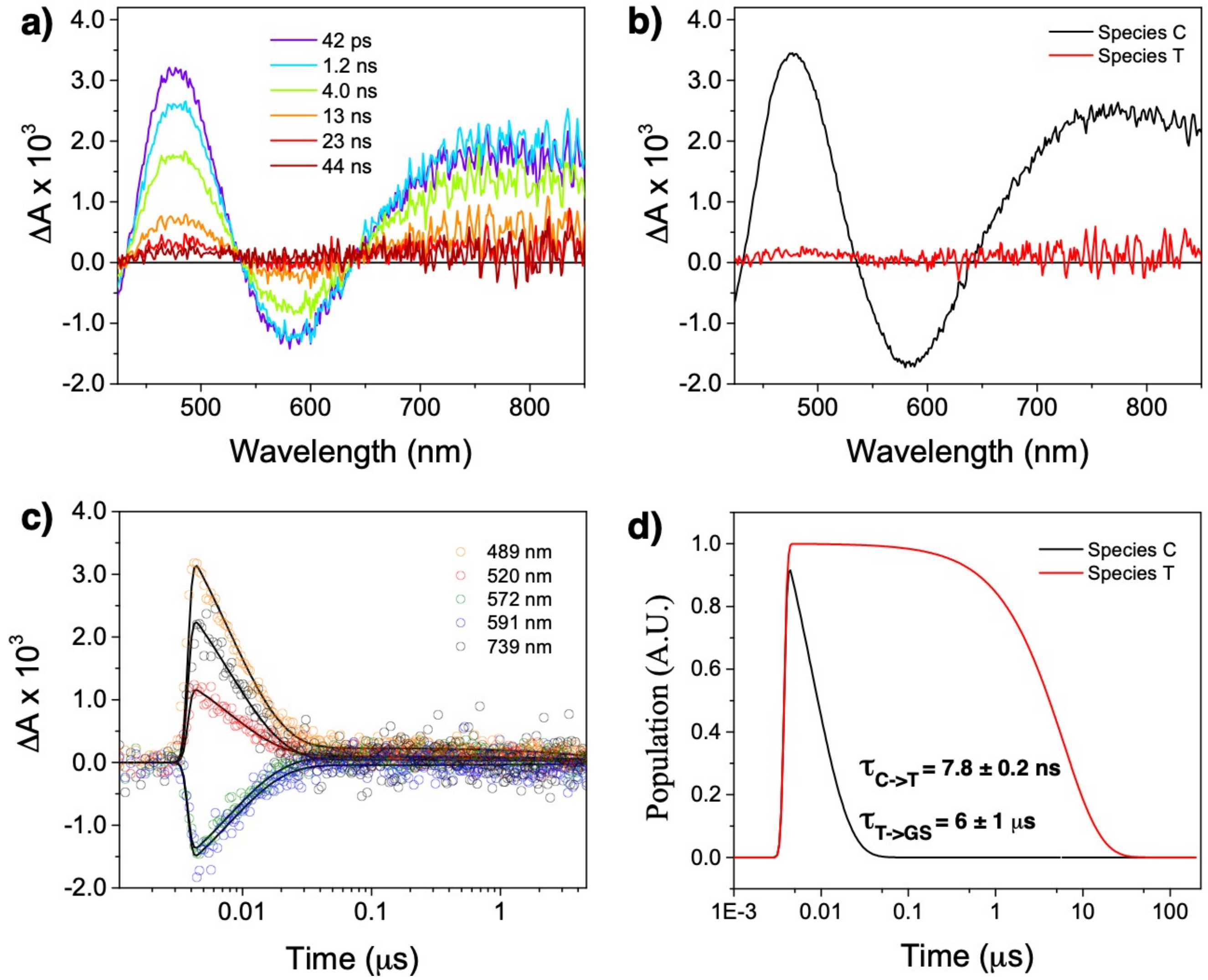

Figure S24. (a) nsTA spectra of G_1-CB[7]$_2$ complex of **Ant910Me** in D_2O (in air) at 298 K following excitation at 414 nm (1 μJ/pulse); (b) decay associated spectra from biexponentially fitting the raw data; (c) wavelength fitting, and (d) model population kinetics.

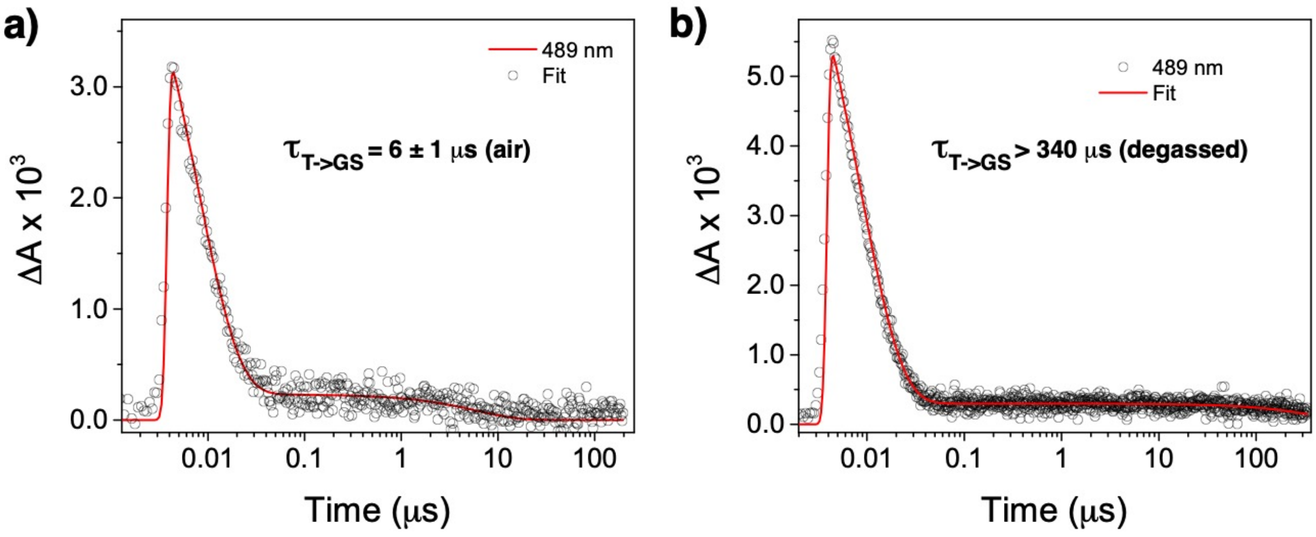

Figure S25. nsTA kinetic trace at 489 nm of G_1-CB[7]$_2$ complex of **Ant910Me** (a) in air and (b) in deoxygenated D_2O.

Table S2. Time constants from fsTA and nsTA of G_2-CB[8]$_2$ and G_1-CB[7]$_2$ complex of Ant910Me

fsTA	Complex of **Ant910Me**	fsTA τ_1 (ps)	fsTA τ_2 (ps)
Excitation 414 nm	G_2-CB[8]$_2$ (air)	3.6 ± 0.3	-
Visible + NIR probe	G_1-CB[7]$_2$ (air)	1.8 ± 0.1	1130 ± 50
Excitation 355 nm	G_2-CB[8]$_2$ (air)	3.4 ± 0.1	-
Visible + NIR probe	G_1-CB[7]$_2$ (air)	1.2 ± 0.1	1200 ± 100

nsTA	complex of **Ant910Me**	nsTA τ_1 (ns)	nsTA τ_2 (µs)
	G_2-CB[8]$_2$ (air)	12.9 ± 0.4	-
Excitation 414 nm	G_1-CB[7]$_2$ (air)	7.8 ± 0.2	6 ± 1
	G_1-CB[7]$_2$ (degassed)	8.4 ± 0.2	> 340

SI-15 MD simulation of Ant910Me$_2$-CB[8]$_2$

Molecular dynamics (MD) simulations were performed with the NAMD 2.12[10] program using the Amber generalized force-field and the RESP charge model.[11] The G_2-Q_2 (Q = CB[8], G = **Ant910Me**) complex was solvated in a TIP3P cubic water box with 4000 H_2O molecules using the Packmol software package.[12] The system was neutralized by adding 4 Cl^{-} ions to the G_2-Q_2 system.

Our MD protocol consisted of the following steps: (1) energy minimization over 10000 steps using a timestep of 1 fs; (2) equilibration over 2 ns in the NVT ensemble (T = 298.15 K) with the RMSD of heavy atoms in CB[8] and **Ant910Me** constrained to their initial position using a force constant of 1 kcal·mol^{-1}·Å^{-2}; (3) 2 ns equilibration run in the NPT ensemble (p = 1.01325 bar, T = 298.15 K); (4) 200 ns production run in the NPT ensemble, where the temperature and pressure were held constant at 298.15 K and 1.0 atm, respectively. Constant temperature was set by a Langevin thermostat with a collision frequency of 1 ps^{-1}, whereas the target pressure was reached using the Berendsen barostat. All of the bonds and angles involving hydrogen atoms were constrained by the SHAKE[13] algorithm. We used the particle mesh Ewald method[14] for the long-range electrostatics in combination with a 12 Å cutoff for the evaluation of the non-bonded interactions. Trajectories were run with a time step of 2 fs and the coordinates were printed out in every 500 steps and the last 120 ns were used for the analysis.

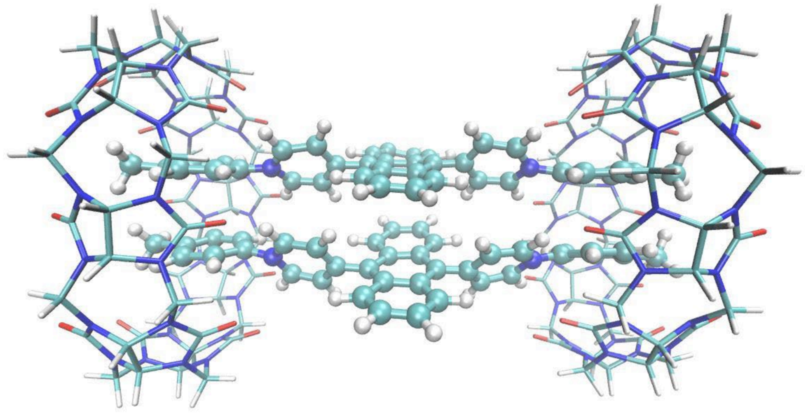

SI-16 Head-to-head and head-to-tail stacking

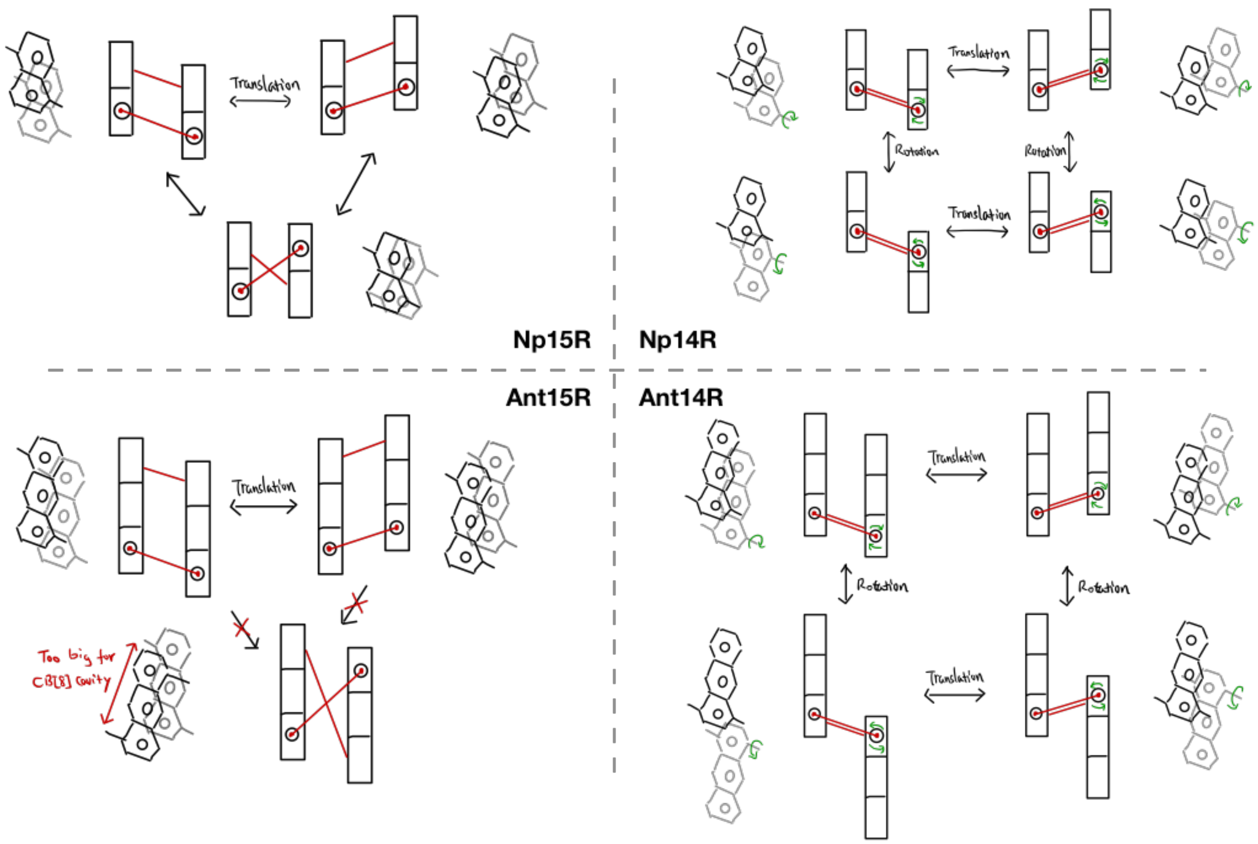

Scheme S3. Possible stacking configurations of two fluorophore cores and their transformations.

Reference

[1] Day, A.; Arnold, A. P.; Blanch, R. J.; Snushall, B. *J. Org. Chem.* **2001**, *66*, 8094–8100.
[2] Sanders, J. K. M.; Brian, K. Modern NMR Spectroscopy, a Guide for Chemists; Oxford University. Oxford. GB, 1993.
[3] Eaton, S.W.; Shoer, L.E.; Karlen, S.D.; Dyar, S.M.; Margulies, E.A.; Veldkamp, B.S.; Ramanan, C.; Hartzler, D.A.; Savikhin, S.; Marks, T.J.; Wasielewski, M.R. *J. Am. Chem. Soc.* **2013**, *135,* 14701–14712.
[4] Brautigam, C. A.; Zhao, H.; Vargas, C.; Keller, S.; Schuck, P. *Nat. Protocols* **2016**, *11*, 882–894.
[5] Ronson, T. K.; Meng, W.; Nitschke, J. R. *J. Am. Chem. Soc.* **2017**, *139,* 9698–9707.
[6] Bongard, D.; Möller, M.; Rao, S. N.; Corr, D.; Walder, L. *Helv. Chim. Acta* **2005,** *88,* 3200–3209.
[7] VanVeller, B.; Robinson, D.; Swager, T.M. *Angew. Chem. Int. Ed.* **2012**, *51*, 1182–1186.
[8] Wheeler, S.E.; McNeil, A.J.; Müller, P.; Swager, T.M.; Houk, K.N. *J. Am. Chem. Soc.* **2010**, *132,* 3304–3311.
[9] Olesińska, M. Design, synthesis and characterisation of π-extended viologen-based molecular and supramolecular systems with cucurbit[n]urils. Ph.D. Dissertation, University of Cambridge, UK, 2019.
[10] Phillips, J. C.; Braun, R.; Wang, W.; Gumbart, J.; Tajkhorshid, E.; Villa, E.; Chipot, C.; Skeel, R. D.; Kalé, L.; Schulten, K. *J. Comput. Chem.* **2005**, *26*, 1781–1802.
[11] D.A. Case, I.Y. Ben-Shalom, S.R. Brozell, D.S. Cerutti, T.E. Cheatham, III, V.W.D. Cruzeiro, T.A. Darden, R.E. Duke, D. Ghoreishi, M.K. Gilson, H. Gohlke, A.W. Goetz, D. Greene, R Harris, N. Homeyer, S. Izadi, A. Kovalenko, T. Kurtzman, T.S. Lee, S. LeGrand, P. Li, C. Lin, J. Liu, T. Luchko, R. Luo, D.J. Mermelstein, K.M. Merz, Y. Miao, G. Monard, C. Nguyen, H. Nguyen, I. Omelyan, A. Onufriev, F. Pan, R. Qi, D.R. Roe, A. Roitberg, C. Sagui, S. Schott-Verdugo, J. Shen, C.L. Simmerling, J. Smith, R. Salomon-Ferrer, J. Swails, R.C. Walker, J. Wang, H. Wei, R.M. Wolf, X. Wu, L. Xiao, D.M. York and P.A. Kollman (2018), AMBER 2018, University of California, San Francisco.
[12] Martínez, L.; Andrade, R.; Birgin, E. G.; Martínez, J. M. *J. Comput. Chem.* **2009**, *30*, 2157–2164.
[13] Andersen, H. C. *J. Comput. Phys.* **1983**, *52*, 24–34.
[14] Darden, T.; York, D.; Pedersen, L. *J. Chem. Phys.* **1993**, *98*, 10089–10092.
